# A practical synthesis of nitrone-derived C5a-functionalized isofagomines as protein stabilizers to treat Gaucher disease

**DOI:** 10.1038/s42004-024-01164-9

**Published:** 2024-04-20

**Authors:** Huang-Yi Li, Wei-An Chen, Hung-Yi Lin, Chi-Wei Tsai, Yu-Ting Chiu, Wen-Yi Yun, Ni-Chung Lee, Yin-Hsiu Chien, Wuh-Liang Hwu, Wei-Chieh Cheng

**Affiliations:** 1 Genomics Research Center, Academia Sinica, 128, Section 2, Academia Road, Taipei, 11529 Taiwan; 2 Department of Pediatrics and Medical Genetics, National Taiwan University Hospital, 8 Chung-Shan South Road, Taipei, 10041 Taiwan; 3 Center for Precision Medicine, China Medical University Hospital, 2, Yude Road, Taichung, 404327 Taiwan; 4 Department of Chemistry, National Cheng-Kung University, 1, University Road, Tainan, 701 Taiwan; 5 Department of Applied Chemistry, National Chiayi University, 300, Xuefu Road, Chiayi, 600 Taiwan; 6 Department of Medicinal and Applied Chemistry, Kaohsiung Medical University, 100, Shih-Chuan 1st Road, Kaohsiung, 807 Taiwan

**Keywords:** Natural product synthesis, Drug discovery and development, Synthetic chemistry methodology, Combinatorial libraries

## Abstract

Isofagomine (IFG) and its analogues possess promising glycosidase inhibitory activities. However, a flexible synthetic strategy toward both C5a-functionalized IFGs remains to be explored. Here we show a practical synthesis of C5a-*S* and *R* aminomethyl IFG-based derivatives via the diastereoselective addition of cyanide to cyclic nitrone **1**. Nitrone **1** was conveniently prepared on a gram scale and in high yield from inexpensive (−)-diethyl d-tartrate via a straightforward method, with a stereoselective Michael addition of a nitroolefin and a Nef reaction as key steps. A 268-membered library (134 × 2) of the C5a-functionalized derivatives was submitted to enzyme- or cell-based bio-evaluations, which resulted in the identification of a promising β-glucocerebrosidase (GCase) stabilizer demonstrating a 2.7-fold enhancement at 25 nM in p.Asn370Ser GCase activity and a 13-fold increase at 1 μM in recombinant human GCase activity in Gaucher cell lines.

## Abstract (web summary)

Isofagomine (IFG) and its analogs have promising glycosidase inhibitory activities, however, a flexible synthetic strategy toward C5a-functionalized IFGs remains to be achieved. Here, the authors show a practical synthesis of C5a-*S* and *R*-aminomethyl IFG derivatives via diastereoselective addition of cyanide to cyclic nitrone, and the synthesized derivatives are subsequently assessed for their efficacy as GCase stabilizers.

## Introduction

**Fig. 1:**
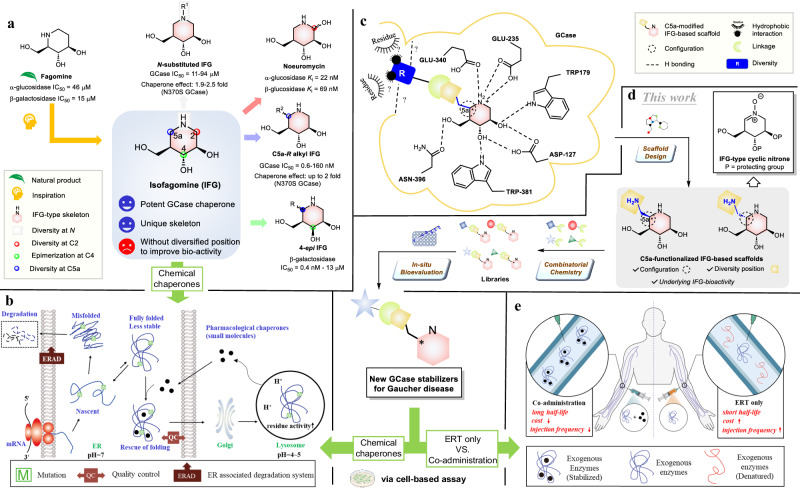
Design and general strategy to discover new GCase stabilizers for potential treatment of Gaucher disease. **a** Structures of fagomine, isofagomine (IFG), and their derivatives with unique IFG skeleton and promising bioactivities. **b** Mechanism of pharmacological chaperones. **c** Schematic diagram of interactions between GCase and IFG-based molecules to design (**d**) C5a-aminomethyl IFG-based scaffolds using cyclic nitrone **1** as the key intermediate followed by combinatorial parallel synthesis and in-situ biological evaluation to develop GCase stabilizers for chemical chaperones and (**e**) co-administration.

The use of small molecule protein stabilizers (SMPSs) capable of delivering a therapeutic benefit by specifically binding to a protein or protein-protein complex is an emerging strategy in drug development^1–3^, and SMPSs have been developed to treat nephrogenic diabetes insipidus^4,5^, cystic fibrosis^6^, and lysosomal storage diseases (LSDs)^7–9^. One example of an SMPS is isofagomine (IFG), a structural isomer of fagomine which has demonstrated the potential to treat Gaucher disease (GD), an LSD caused by the accumulation of glucosylceramide (GlcCer) due to genetic mutations in β-glucocerebrosidase (GCase, CAZy family GH30, EC. 3.2.1.45), and leading to enlargement of affected organs, bone lesions, and even central nervous system impairment (Fig. 1a)^10–14^. In addition to substrate reduction therapy using Eliglustat^15^, the current standard of care for GD is enzyme replacement therapy (ERT) with recombinant human GCase (rh-GCase)^16^, but its intrinsic instability at neutral pH and body temperature urges scientists to investigate new strategies to overcome this limitation^17^. Our initial attempts to prolong it for example by polyethylene glycol (PEG) conjugation, a commonly used approach to stabilize proteins, were unsuccessful (Supplementary Fig. 1)^18,19^.

The potential role for SMPS in GD is therefore two-fold: act as a chemical chaperone to bind and stabilize mutant GCase in the endoplasmic reticulum (ER), facilitating proper protein folding and translocation to the lysosome, thereby increasing GCase cellular activity (Fig. 1b)^20^; and/or improve the stability of rh-GCase in the presence of SMPS under physiological conditions (Fig. 1e). One SMPS, isofagomine (IFG), for GD is also a reversible competitive inhibitor against GCase. This implies the necessity of utilizing sub-inhibitory concentrations to facilitate substrate turnover, thereby augmenting GCase activity in GD patients during clinical trials^21^. Furthermore, the unique skeleton of IFG and its versatile bioactivity have prompted the development of various approaches to its synthesis, and that of its analogues, some of which have been reported to inhibit several enzymes including *N*-substituted IFG, noeuromycin (2-hydroxy IFG), and 4-*epi*-IFG^13,22–25^. The improved bioactivities demonstrated by some of these analogues imply that more flexible synthetic routes toward the preparation of IFG-inspired skeletons and their corresponding derivatives are needed to identify more potent biomolecules (Fig. 1a)^26–34^.

To develop a superior IFG-based enzyme stabilizer or chemical chaperone toward the wild-type or mutant GCase, structural modification of IFG at its C5a position is necessary based on structural perspectives of the co-crystal structure of rh-GCase with IFG (Fig. 1c)^14^. To the best of our knowledge, only one synthetic approach toward C5a-alkyl IFGs has been reported (Fig. 1a)^35^, but its synthetic flexibility is not extendable as well as the usage of a rare and expensive α-l-xylopyranoside as a starting material. For example, the synthetic strategy toward the preparation of C5a-*S* modified IFGs is not available and remains to be explored. Therefore, developing a practical synthetic pathway to prepare both C5a-*S* and *R* functionalized IFGs, enabling the discovery of next-generation bioactive compounds as SMPSs for modulating GCase activities, is a challenge in chemistry and directly needed.

We have recently harnessed natural product-inspired combinatorial chemistry (NPICC) to efficiently synthesize bicyclic alkaloid-based scaffolds and their corresponding libraries to create a unique chemical space, which allows us to identify new selective Golgi α-mannosidase II inhibitors^36^. We herein planned to adopt the NPICC approach to design and synthesize two new IFG-based scaffolds incorporating a C5a-aminomethyl moiety of different configurations from IFG-typed cyclic nitrone **1**, allowing us to rapidly increase chemical space via conjugation with a structurally diverse carboxylic acid library (Fig. 1d)^37–39^. The resulting IFG derivatives were assessed for their ability to stabilize GCase and rh-GCase.

## Results and discussion

**Fig. 2:**
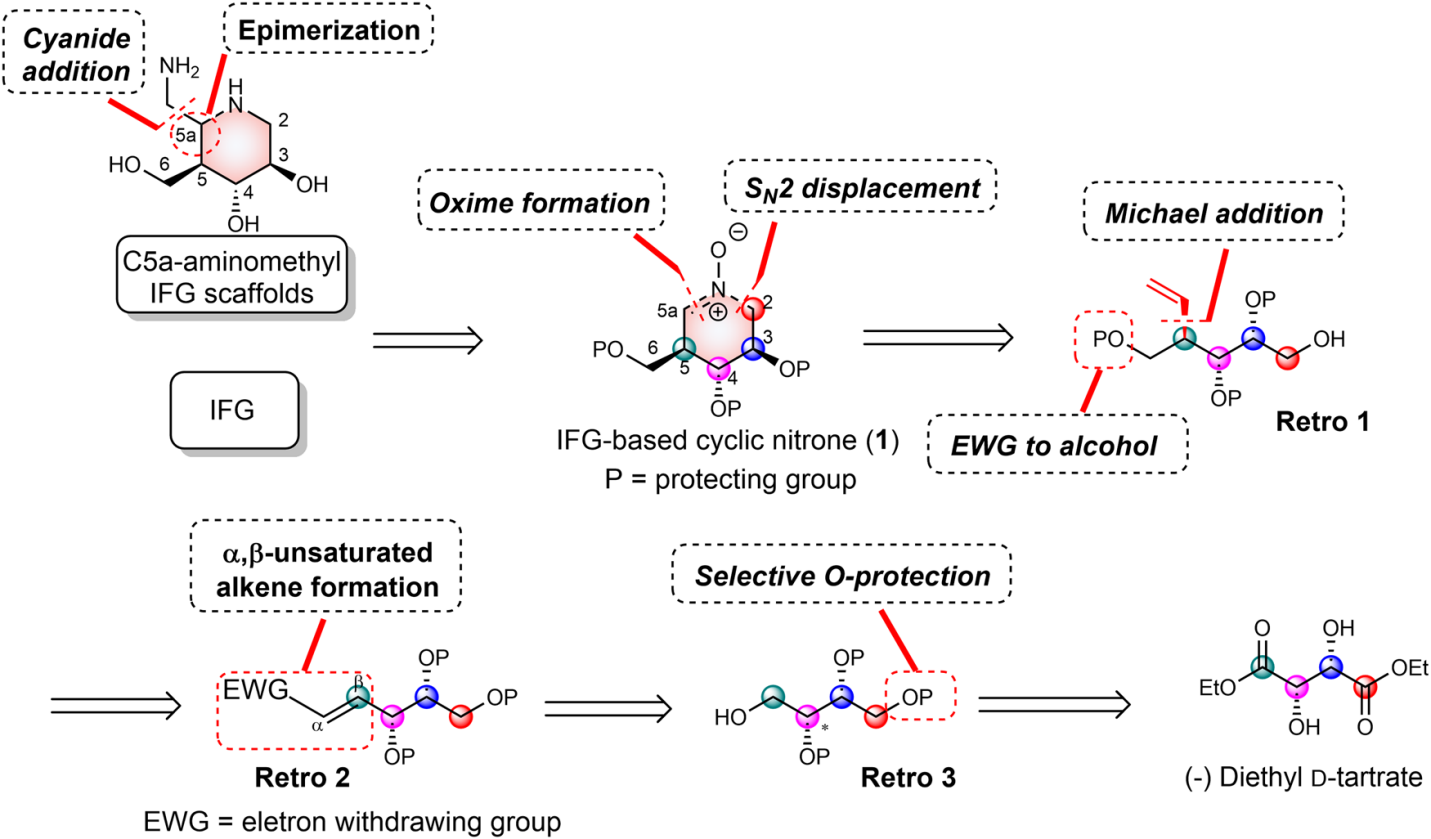
Retrosynthetic analysis of C5a-aminomethyl IFG-based scaffolds and cyclic nitrone 1. The retrosynthetic plan of desired C5a-aminomethyl IFG scaffolds from (−)-diethyl d-tartrate.

The retrosynthetic analysis of C5a-aminomethyl IFG scaffolds is depicted in Fig. 2. Installation of the C5a-aminomethyl moiety of IFG was envisioned by a selective nucleophilic addition of cyanide to cyclic nitrone **1** at the C5a position where the newly generated stereocenter could be further inverted via an oxidation-reduction sequence, potentially doubling the size of the compound library^37,40^. Via oxime formation followed by S_N_2 displacement, nitrone **1** could be derived from acyclic **Retro 1**, furnished from **Retro 2** by Michael addition to the α,β-unsaturated alkene moiety followed by functional group manipulations to convert the electron-withdrawing group (EWG) to a protected alcohol. **Retro 2** could be obtained from **Retro 3** by a series of chemical transformations including alcohol oxidation, nucleophilic addition, and elimination to construct the Michael acceptor moiety. **Retro 3** was envisioned to be accessed by (−)-diethyl d-tartrate by selective *O*-protection and ester reduction.

**Fig. 3:**
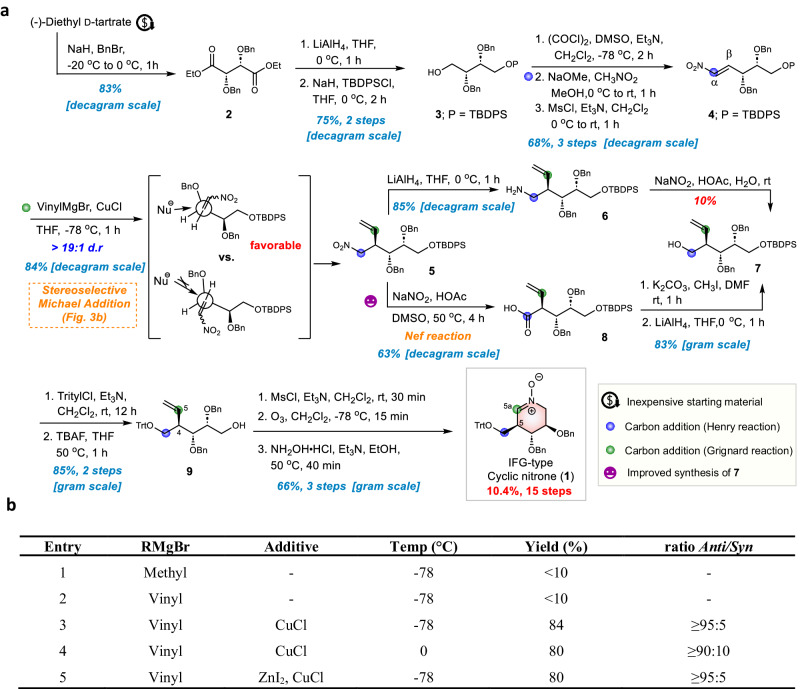
Preparation of cyclic nitrone 1 from (−)-diethyl d-tartrate. **a** Synthetic pathway of IFG-type cyclic nitrone **1**. **b** Optimization of Grignard Michael addition of **4**.

Figure 3a depicts our synthesis of **1**. Diester **2**, prepared from the inexpensive chiral material (−)-diethyl d-tartrate, was first reduced with LiAlH_4_ to give a diol, which underwent selective silyl *O*-protection with TBDPSCl to give alcohol **3** in 75% yield over two steps^41^. Compound **3** was then converted to α,β-unsaturated nitroalkene **4** via Swern oxidation, Henry reaction, and β-elimination in a yield of 68% over three steps. Grignard addition of methylMgBr and vinylMgBr to nitroolefin **4** at −78 °C did not yield the desired 1,4 adducts (Entry 1 and 2, Fig. 3b)^42^. However, the diastereoselective Michael addition of vinylic cuprates (vinylmagnesium bromide with CuCl) to nitroalkene **4** exclusively furnished the desired 1,4 adduct **5** (anti/syn ≧ 95:5) in good yield (84%) (Entry 3, Fig. 3b); the diastereoselectivity of the reaction was consistent with the Felkin-Anh model^43,44^. Neither changing the temperature from −78 °C to 0 °C nor adding ZnI_2_ improved either the selectivity or overall yield (Entries 4 and 5, Fig. 3b).

Intermediate **5** was easily reduced to amine **6** (85%) but the conversion of amine **6** to alcohol **7** was poorly yielding (<10%) even under several modified conditions (Supplementary Fig. 2). The problem was solved by converting the nitro group of **5** into the corresponding carboxylic acid to yield compound **8** (63%) via a Nef reaction (NaNO_2_ in HOAc)^45,46^. Alcohol **7** was then successfully obtained in 83% yield from acid **8** in the typical two-step sequence (esterification and reduction), and then easily advanced to **9** by protection of the primary hydroxyl with trityl chloride and deprotection of the TBDPS group. Treatment of **9** with MsCl under basic conditions (Et_3_N) followed by ozonolysis and oxime formation with hydroxylamine gave cyclic nitrone **1** (66% over three steps). A newly developed six-membered cyclic nitrone **1** with the same configuration pattern as isofagomine; **1** also bears an electrophilic nitrone moiety at C5a suitable for a library of derivatives, structurally diverse at that position, to be synthesized.

**Fig. 4:**
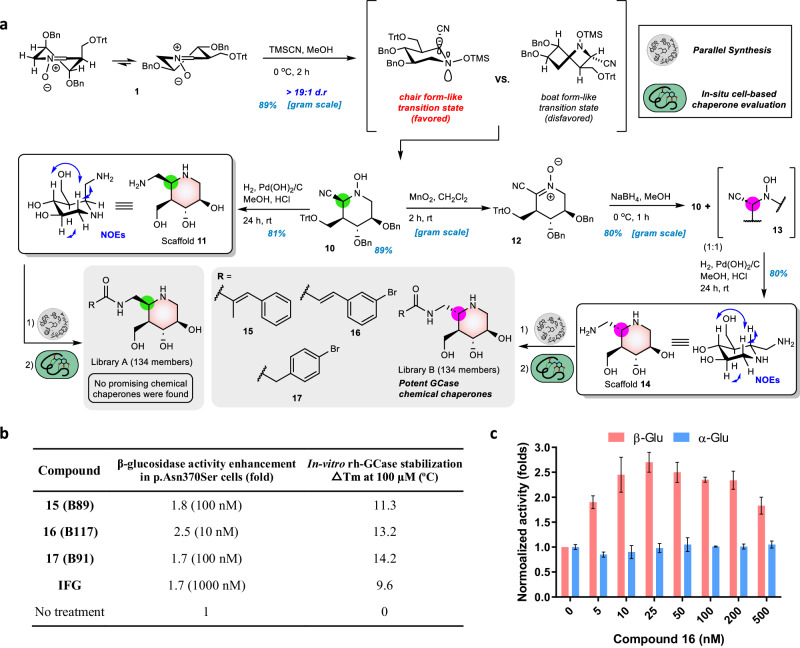
Synthesis of compound libraries and evaluation of selected compounds. **a** Scaffold **11** and scaffold **14** were synthetically prepared to generate library A and library B for in-situ cell-based chaperone screening in Gaucher fibroblasts (GM00372), respectively. Several selected hits were found, resynthesized, and further characterized as potent GCase chemical chaperones. **b** The β-glucosidase activity enhancement effects of IFG, **15,** **16**, and **17** in Gaucher fibroblasts (GM00372) and the stabilization of rh-GCase with these small molecules at 100 µM. The fold change in enzyme activity is compared to untreated cells (normalized value = 1). The maximal fold increase was observed at a given compound concentration. Data are the mean of three determinations.ΔTm (^o^C) refers to melting temperature change compared to Tm of rh-GCase in the absence of small molecules. **c** The influence of **16** on α- and β-glucosidase activities in GM00372 fibroblasts. Enzyme activity is normalized to untreated cells and assigned a relative activity of 1.

The selective nucleophilic addition of TMSCN to cyclic nitrone **1** gave adduct **10** (89%) as a single diastereomer (Fig. 4a). The newly generated chiral center of cyanide **10** can be accounted for by anti-periplanar lone pair theory, in which the silane reagent chelates to oxygen of the nitrone moiety to generate a chair-like transition state as the favored form, which is attacked by the cyanide at the axial position to yield a major C5a-*S* cyano IFG adduct^47^. After catalytic hydrogenation of **10**, the desired C5a-*S*-aminomethyl IFG **11** was obtained in 81% yield and its configuration was confirmed by 2D NOESY analysis. Notably, isofagomine could be easily obtained in 90% yield from cyclic nitrone **1** by acid-mediated hydrogenation (Pd(OH)_2_/C, H_2_, conc. HCl, MeOH). Next, C5a-epimerization of **10** was performed via an oxidation and reduction sequence; after global deprotection, the C5a-*R* aminomethyl IFG **14** —another desired scaffold—was obtained (Fig. 4a)^40,48^.

With initial scaffolds **11** and **14** in hand, corresponding libraries A and B were efficiently prepared in a parallel combinatorial approach, by individually coupling them with 134 different carboxylic acids (Fig. 4a and Supplementary Fig. 3)^49^. Each carboxylic acid was activated using HBTU (1.2 equiv.), and DIPEA (3 equiv.) in DMSO. Reactions were monitored by TLC and LC-MS, and conversion was found to be almost complete after 48 h (Supplementary Table 1). Reactions were quenched by dilution with an aqueous buffer. All the products derived from **11** and **14** were directly diluted and evaluated in an in-situ screening of rh-GCase inhibition and a cell-based chaperone screening against p.Asn370Ser/p.Leu29Alafs*18 Gaucher fibroblasts (GM00372), initially without further purification. The primary screening results showed that some hits in library A are strong inhibitors against rh-GCase (>60% inhibition at a theoretical concentration of 100 nM) (Supplementary Figs. 4a, 4b, 5a, b), but no promising hit exhibits significant chaperoning potency (>1.5 fold β-glucosidase activity enhancement) after normalization by the activity of untreated cells. In particular, **S1** bearing a 2-naphthyl carbonyl moiety (IC_50_ = 3.7 ± 0.4 nM) exhibited a 50-fold greater inhibitory potency than IFG (IC_50_ = 187.0 ± 6.0 nM) (Supplementary Fig. 4e). Taken together, these results indicate that the biological potency of substituent-modified scaffolds can be dramatically improved and that the most potent GCase inhibitors are not always the most effective Gaucher chemical chaperones.

In contrast, several compounds in library B displayed a promising chaperoning effect on β-glucosidase activity in p.Asn370Ser fibroblasts (>1.6 fold enhancement of activity at a theoretical concentration of 1 μM) (Supplementary Fig. 5c, d). Among them, compounds **15,** **16**, and **17** were resynthesized and characterized (Fig. 4a); the chaperone activity study toward p.Asn370Ser fibroblasts demonstrated **15** (1.8-fold β-glucosidase activity enhancement at 100 nM) and **17** (1.7-fold β-glucosidase activity enhancement at 100 nM) exhibited a moderate enhancement behavior, similar to the reference compound IFG (Fig. 4b and Supplementary Fig. 6)^50^. Furthermore, compound **16** showed significant chaperoning activity – a 2.5-fold enhancement of β-glucosidases activity at a concentration of 10 nM, suggesting mutant GCase stabilization (Fig. 4b and Supplementary Fig. 6). Notably, this is the first report to demonstrate that IFG derivatives bearing C5a-*R* functionalization can serve as more potent β-glucosidase chaperones than those bearing C5a-*S* functionalization, and IFG itself ^35,51^.

Next, compounds **15,** **16**, and **17** were submitted to enzyme-based characterization, including thermal shift and inhibition studies. The enzyme melting temperatures (Tm) of rh-GCase in the presence of each were separately measured in a fluorescence-based thermal denaturation assay to investigate their effect on the thermal stabilization of rh-GCase. Co-incubation of rh-GCase with **15,** **16**, and **17** at pH 7.0 resulted in a dose-dependent stabilization of rh-GCase, which displayed larger melting temperature changes (ΔTm) at 100 µM as 11.3 °C, 14.2 °C, and 13.2 °C, respectively, compared to IFG (9.6 °C). (Fig. 4b and Supplementary Fig 7). In addition, compounds **15** (IC_50_ = 30.0 ± 7.8 nM), **16** (IC_50_ = 4.0 ± 0.7 nM), and **17** (IC_50_ = 5.1 ± 1.1 nM) also showed better potency in GCase inhibition than IFG (IC_50_ = 187.0 ± 6.0 nM). Although the GCase inhibitory activities of **16** (IC_50_ = 4.0 ± 0.7 nM) and **S1** (IC_50_ = 3.7 ± 0.4 nM) were similar, their chaperoning potencies were dramatically different, suggesting these two properties to be differentially affected by the substituents on IFG-based scaffolds.

Based on its promising chaperoning activity and excellent ΔTm, we next investigated the inhibitory selectivity of **16** toward α-glucosidases and β-glucosidases in p.Asn370Ser fibroblasts. As shown in Fig. 4c, **16** satisfactorily enhanced p.Asn370Ser β-glucosidase activity up to 2.7-fold at 25 nM without impairing cellular α-glucosidase activity over a wide concentration range (0–500 nM), suggesting it is worthy of further investigations as a potential treatment of GD via the selective stabilization of GCase.

**Fig. 5:**
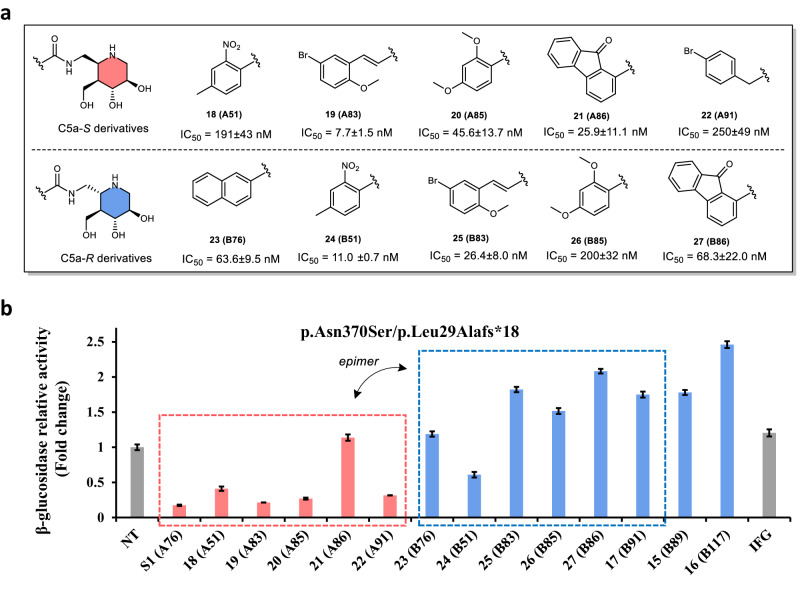
Structures of resynthesized IFG derivatives and bioevaluation results. **a** Chemical structure of the resynthesized IFG derivatives **18**–**27** and their GCase inhibitory activities. **b** The influence of **15**–**27,** **S1**, and IFG (100 nM) toward cellular β-glucosidase activity in p.Asn370Ser/p.Leu29Alafs*18 Gaucher fibroblasts (GM00372). Enzyme activity is normalized to untreated cells, assigned a relative activity of 1. Mean values ± SD are shown for triplicate experiments. NT refers to cells without treatment of molecules.

To confirm that the C5a-*R* functionalized IFG derivatives exhibit superior chaperoning activity compared to C5a-*S* derivatives, we further resynthesized compounds **18–27**, including the potent inhibitors, the effective chaperones and their corresponding epimers based on the in-situ screening data (Fig. 5a). The ability of IFG derivatives to improve β-glucosidases activity was then assessed in p.Asn370Ser fibroblasts (Fig. 5b). Notably, most IFG derivatives **15–17** and **25–27** bearing the C5a-*R* configuration showed promising enhancement effects (>1.5-fold) on β-glucosidase activities at 100 nM compared to compounds **S1** and **18–22** with the C5a-*S* configuration and IFG at the same concentration. This observation supports that the C5a-*R* functionalized derivatives possess superior chaperoning activity than the C5a-*S* derivatives and IFG at nanomolar concentration. In addition, there was no clear correlation observed between their inhibitory and chaperoning activity (Fig. 5a, b). Both findings are consistent with the results obtained from the enzyme and cell-based in-situ screening of libraries A and B.

**Fig. 6:**
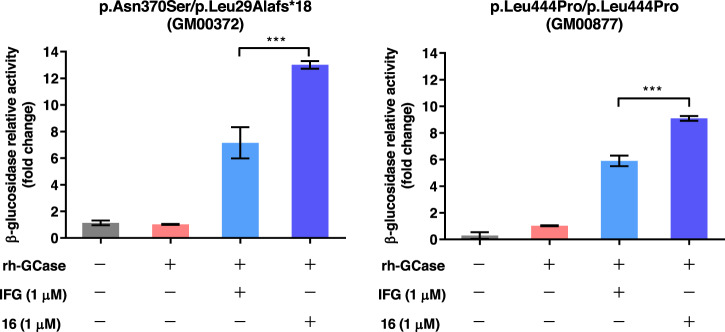
Enhancement effects of compound 16 on β-glucosidase in Gaucher fibroblasts. Co-administration of rh-GCase (0.17 μM) with small molecule **16** (1 μM) significantly increased β-glucosidase activity in Gaucher fibroblasts (GM00372 and GM00877). Enzyme activity is normalized to cells treated only with rh-GCase, assigned a relative activity of 1. Mean values ± SD are shown for triplicate experiments. ***p < 0.005 (One-way ANOVA with Tukey’s post-hoc).

Finally, to investigate whether the most effective chemical chaperone **16** could enhance the ERT efficacy, we incubated Gaucher fibroblasts (GM00372 and GM00877) with rh-GCase in the presence and absence of the iminosugar IFG or **16** for 24 h. Co-administration of rh-GCase (0.17 μM) with **16** (1 μM) resulted in a 13-fold activity enhancement in Gaucher p.Asn370Ser fibroblasts compared to rh-GCase alone, and a 9.1-fold activity enhancement in p.Leu444Pro Gaucher fibroblasts with the common point mutation correlating with neuronopathic GD (Fig. 6)^52^. These results were significantly better than the enzyme activity enhancement by co-administration of rh-GCase with IFG (7-fold and 6-fold activity enhancement in p.Asn370Ser and p.Leu444Pro fibroblasts, respectively), proving that **16** can stabilize endogenous mutant GCase and exogenous rh-GCase more effectively than IFG in our cell-based assays.

## Conclusion

A flexible synthesis of a library of C5a-functionalized IFGs has been developed, work which resulted in the identification of compound **16** as a potent SMPS of GCase, and the key finding that C5a-*R* substituted IFG derivatives are more potent chaperones of GCase than C5a-*S* substituted. The library was based on two cyclic-nitrone-**1**-derived C5a-aminomethyl IFGs, which served as key scaffolds for diversification in a combinatorial approach. Nitrone **1** was synthesized from inexpensive (−)-diethyl d-tartrate in high-yield (10.4% over 15 steps on the multigram scale) using stereoselective Michael addition and Nef reaction as the key steps. Compound **16** showed remarkable enhancements of GCase activity in p.Asn370Ser and p.Leu444Pro Gaucher fibroblasts co-treated with rh-GCase, compared with rh-GCase alone. This systematic strategy for rapid preparation and C5a-modification of IFG scaffolds as well as further bioevaluation is promising and feasible to develop next-generation protein stabilizers for GD.

## Methods

### Preparation of library A and B^38,49,53^

In each 200 μL well of a microtiter plate, a mixture of diisopropyl ethylamine (5 μL, 180 mM solution in DMSO), HBTU (5 μL, 72 mM solution in DMSO), a carboxylic acid (10 μL, 30 mM solution in DMSO) and a scaffold **11** or **14** (10 μL, 30 mM solution in DMSO) was shaken at rt for 48 h and analyzed by ESI-MS and TLC to verify the consumption of scaffold **11** or **14** and the presence of the desired products. The resulting crude products were directly screened in the enzyme- or cell-based assay without further purification.

### In-situ screening of rh-GCase inhibition study^54^

The inhibition assays were performed in a total volume of 30 μL in 384-well microtiter plates. The reaction mixture containing the individual products of library A or library B (100 nM, theoretical concentration), 4-methyllumbelliferyl β-d-glucoside (0.3 mM) as substrate, and rh-GCase (0.5 mU) in citrate-phosphate buffer (0.1 M, pH 5.2) were incubated at room temperature for 30 min. The reaction was terminated by the addition of sodium glycine buffer (40 uL, 0.5 M, pH 10.5). Enzyme activity was measured by the release of 4-methylumbelliferone with an excitation wavelength of 355 nm and an emission wavelength of 460 nm (SpectraMax M5, Molecular Devices).

### Cell culture

Fibroblasts derived from Gaucher patients (GM00372 and GM00877) were purchased from Coriell Institute (Camden, NJ). Cells were maintained at 37 °C with 5% CO_2_ in Minimal Essential Medium (MEM, Gibco 41500-34) supplemented with 15% fetal bovine serum (FBS, Gibco 1887826), 1% l-glutamine (Gibco A29168-01), 1% sodium pyruvate (Gibco 11360-070) and 1% non-essential amino acids (Gibco 11140050).

### Intact cell α- and β-glucosidases activity study^55^

GM00372 cells were seeded into 96-well culture plates. After 24 h of attachment, the medium was replaced with a fresh medium containing individual products of library A or B (1 μM, theoretical concentration), or resynthesized compounds including IFG, **15,** **16**, or **17** in a dose-dependent manner. The final concentration of DMSO was <0.5% which had no cytotoxicity or glucosidase inhibition. The cells were incubated for 5 days and the medium for DMSO-treated cells or compound-treated cells was renewed every 3 days. The enzyme activity assay was performed after removing the medium supplemented with compounds. Compounds were evaluated at all concentrations in triplicate. The monolayers were washed with Dulbecco’s PBS solution (Gibco pH 7.4). PBS (16 μL) and acetate buffer (0.2 M, 16 μL, pH 4.0) were added to each well. The reaction was started by the addition of 4-methylumbelliferyl β-d-glucoside (4 mM, 25 μL, Sigma) to each well, followed by incubation at 37 °C for 2 h. The reaction was stopped by lysing the cells with glycine buffer (0.2 M, 200 μL, pH 10.8). The fluorescence was also measured by the release of 4-methylumbelliferone with an excitation wavelength of 355 nm and an emission wavelength of 460 nm (SpectraMax M5, Molecular Devices). The fluorescence of untreated cells was compared with those treated with compounds. Among the resynthesized compounds, compound **16** was further subjected to the same experiments described above thoroughly at the concentration of 0–500 nM. Likewise, 4-methylumbelliferyl α-d-glucoside (4 mM, 25 μL, Sigma) was used to evaluate the cellular α-glucosidase activity in GM00372 cells treated with or without compound **16** (0–500 nM).

### Thermal stability shift assay^56^

The stability of rh-GCase was assessed using a modified fluorescence thermal stability assay on a Rotor-Gene system in a neutral pH buffer (phosphate buffer, pH 7.0). Briefly, rh-GCase (4 μg) was combined with SYPRO Orange and various concentrations of IFG, **15,** **16**, and **17** in a final reaction volume of 20 λ. A thermal gradient was applied to the plate at a rate of 1 °C/minute, during which time the fluorescence of SYPRO Orange was continuously monitored. The fluorescence intensity at each temperature was normalized to the maximum fluorescence after complete thermal denaturation.

### rh-GCase activity in Gaucher patient fibroblasts

Gaucher patient fibroblasts (GM00372 and GM00877) (2 × 10^4^ cells) were seeded in 48-well culture plates and incubated overnight. Cells were treated with IFG or **16** in the presence of 0.17 μM of rh-GCase. After 24 h, the medium was refreshed without rh-GCase and SMPSs, then the cells were incubated for another 24 h. Cells were harvested by trypsinization and the cell pellets were washed twice with PBS and lysed with lysis buffer containing 0.1% Triton X-100, 50 mM Tris-HCl, and 150 mM NaCl. GCase activity assay was performed by mixing lysate (10 μl) and substrate (90 μL of 4 mM 4-methylumbelliferyl β-d-glucopyranoside) in 0.1 M NaOAc and incubating at 37 °C for 1 h. Reactions were terminated by adding 0.2 N glycine-NaOH, pH 10.7. The fluorescence was detected at excitation/emission wavelengths 355/460 nm.

### Statistical analysis

All statistical analysis was conducted using a student’s *t*-test with GraphPad Prism 6 (GraphPad Software, Inc. La Jolla, CA). Data were presented as mean values ± standard deviations. *p* values < 0.05 were deemed statistically significant.

### Reporting summary

Further information on research design is available in the Nature Portfolio Reporting Summary linked to this article.

### Supplementary information

Supplementary Information

Description of Additional Supplementary Files

Supplementary Data 1

reporting summary

## Data Availability

Supplementary methods for the detailed experimental procedures and characterizations of new compounds are available in Supplementary Information. ^1^H and ^13^C NMR spectra can be found in the Supplementary Data 1. All other data are available from the corresponding author upon reasonable request.
