# Supplementary material for: A practical synthesis of nitrone-derived C5a-functionalized isofagomines as protein stabilizers to treat Gaucher disease
Source: Commun Chem. 2024 Apr 20;7:91. doi: 10.1038/s42004-024-01164-9 (PMC11032326; doi:10.1038/s42004-024-01164-9)
Supplement: Supplementary file 1 — Supplementary Information [file 42004_2024_1164_MOESM1_ESM.pdf]

Supplementary Information

A Practical Synthesis of Nitrone-derived C5a-Functionalized Isofagomines as Protein Stabilizers to Treat Gaucher Disease

Huang-Yi Li[1#], Wei-An Chen[1#], Hung-Yi Lin[1#], Chi-Wei Tsai[1], Yu-Ting Chiu[1], Wen-Yi Yun[1], Ni-Chung Lee[2], Yin-Hsiu Chien[2], Wuh-Liang Hwu[2,3] and Wei-Chieh Cheng[1,4,5,6*]

[1] Genomics Research Center, Academia Sinica, 128, Section 2, Academia Road, Taipei, 11529, Taiwan

[2] Department of Pediatrics and Medical Genetics, National Taiwan University Hospital, 8 Chung-Shan South Road, Taipei 10041, Taiwan

[3] Center for Precision Medicine, China Medical University Hospital, 2, Yude Road, Taichung 404327, Taiwan

[4] Department of Chemistry, National Cheng-Kung University, 1, University Road, Tainan 701, Taiwan

[5] Department of Applied Chemistry, National Chiayi University, 300, Xuefu Road, Chiayi 600, Taiwan

[6] Department of Medicinal and Applied Chemistry, Kaohsiung Medical University, 100, Shih-Chuan 1st Road, Kaohsiung 807, Taiwan

[#] These authors contributed equally

[*] Corresponding author (email: wcheng@gate.sinica.edu.tw).

A

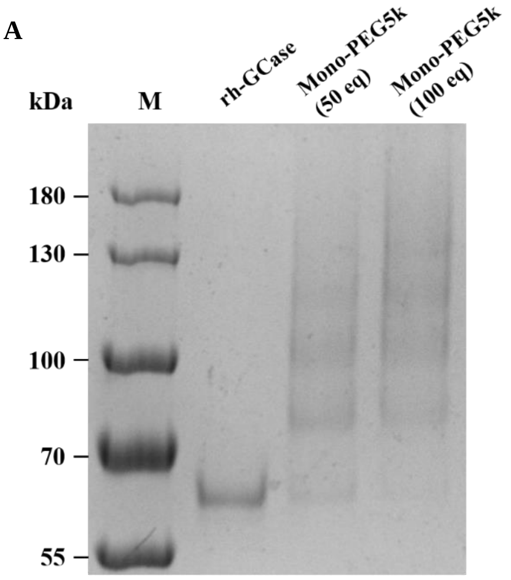

B

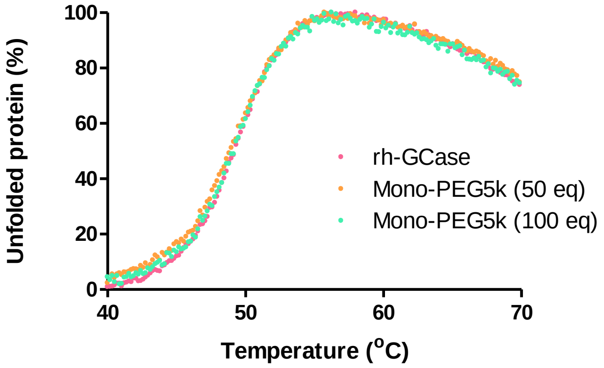

Sample ID	Tm (ºC)
rh-GCase	49.1 ± 0.1
Mono-PEG5k (50 eq)	51.7 ± 0.3
Mono-PEG5k (100 eq)	50.3 ± 0.4

Supplementary Figure 1. Characterization of rh-GCase modified by MeO-PEG-NHS (Mw 5000 Da) with different molar ratios of reagent to the enzyme. (A) SDS-PAGE analysis of the pegylated rh-GCases indicated the acceptable effectiveness of the modification reaction, but reacting rh-GCase with longer MeO-PEG-NHS reagents resulted the incomplete pegylation (data not shown). (B) The purified pegylated rh-GCases were evaluated by a fluorescence-based thermal shift assay, revealing that the thermal stability of rh-GCase was barely improved by PEGylation with MeO-PEG-NHS (Mw 5000 Da).

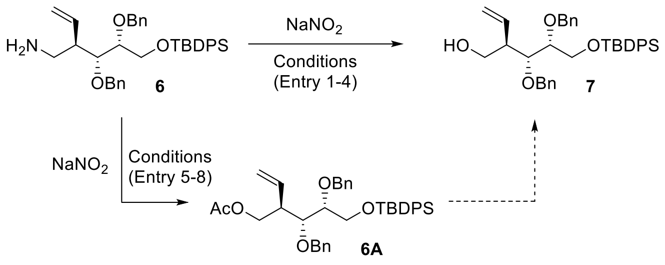

Entry	Conditions	Temp (°C)	Isolated yield (%)[a]
1	$NaNO_2$, HOAc/H_2O	0	10
2	$NaNO_2$, HOAc/H_2O,	rt	10
3	$NaNO_2$, HCl/H_2O	0	messy
4	$NaNO_2$, HCl/H_2O	rt	messy
5	TFAA, $NaNO_2$, HOAc, Ac_2O	0	10
6	TFAA, $NaNO_2$, HOAc, Ac_2O	rt	15
7	TFAA, $NaNO_2$, HCl, Ac_2O	0	messy
8	TFAA, $NaNO_2$, HCl, Ac_2O	rt	messy

Supplementary Figure 2. Attempted synthesis of 7 from 6.

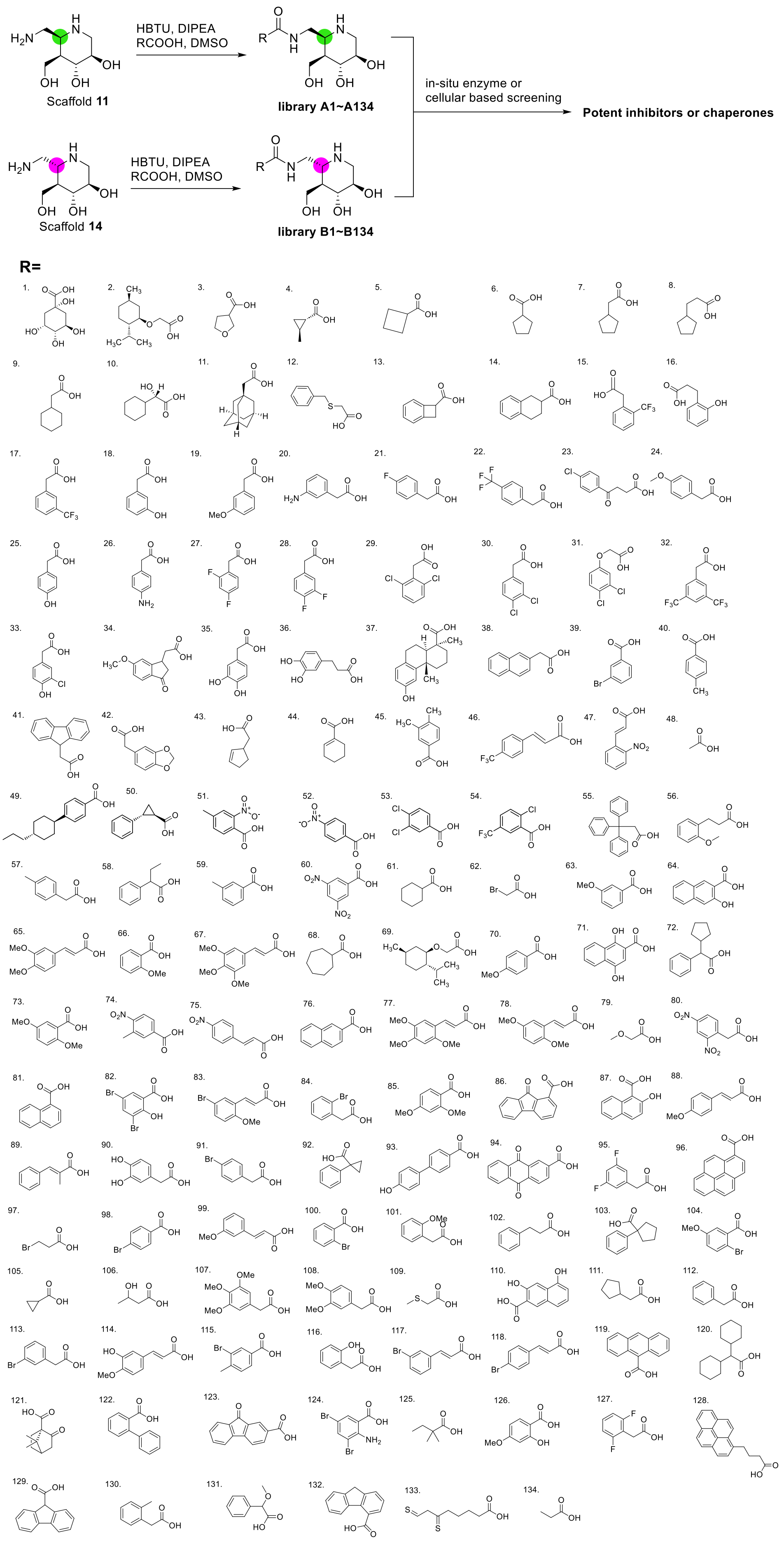

Supplementary Figure 3. Preparation of libraries A and B for *in-situ* screening and carboxylic acid libraries for diversification.

Supplementary Table 1. HRMS data of library A (A1–A134) and library B (A1–A134).

Compounds	Molecular Formula of the desired product	Theoretical m/z	Observed m/z
**A1**	$[C_{14}H_{26}N_2O_8+H]^+$	351.1762	351.1754
**A2**	$[C_{14}H_{36}N_2O_5+H]^+$	373.2697	373.27
**A3**	$[C_{12}H_{22}N_2O_5+H]^+$	275.1601	275.1603
**A4**	$[C_{12}H_{22}N_2O_4+H]^+$	259.1652	259.1647
**A5**	$[C_{12}H_{22}N_2O_4+H]^+$	259.1652	259.1652
**A6**	$[C_{13}H_{24}N_2O_4+H]^+$	273.1809	273.1813
**A7**	$[C_{14}H_{26}N_2O_4+H]^+$	287.1965	287.1964
**A8**	$[C_{15}H_{28}N_2O_4+H]^+$	301.2122	301.2126
**A9**	$[C_{15}H_{28}N_2O_4+H]^+$	301.2122	301.2126
**A10**	$[C_{15}H_{28}N_2O_5+H]^+$	317.2071	317.2068
**A11**	$[C_{19}H_{32}N_2O_4+H]^+$	353.2435	353.2435
**A12**	$[C_{16}H_{24}N_2O_4S+H]^+$	341.153	341.1527
**A13**	$[C_{16}H_{22}N_2O_4+H]^+$	307.1652	307.1653
**A14**	$[C_{18}H_{26}N_2O_4+H]^+$	335.1965	335.1969
**A15**	$[C_{16}H_{21}F_3N_2O_4+H]^+$	363.1526	363.1524
**A16**	$[C_{16}H_{24}N_2O_5+H]^+$	325.1758	325.1754
**A17**	$[C_{16}H_{21}F_3N_2O_4+H]^+$	363.1526	363.1527
**A18**	$[C_{15}H_{22}N_2O_5+H]^+$	311.1601	311.1599
**A19**	$[C_{16}H_{24}N_2O_5+H]^+$	325.1758	325.1754
**A20**	$[C_{15}H_{23}N_3O_4+H]^+$	310.1761	310.1764
**A21**	$[C_{15}H_{21}FN_2O_4+H]^+$	313.1558	351.1754
**A22**	$[C_{16}H_{21}F_3N_2O_4+H]^+$	363.1526	363.1525
**A23**	$[C_{17}H_{23}ClN_2O_5+H]^+$	371.1368	371.1371
**A24**	$[C_{16}H_{24}N_2O_5+H]^+$	325.1758	325.1758
**A25**	$[C_{15}H_{22}N_2O_5+H]^+$	311.1601	311.1598
**A26**	$[C_{15}H_{23}N_3O_4+H]^+$	310.1761	310.1763
**A27**	$[C_{15}H_{20}F_2N_2O_4+H]^+$	331.1464	331.1469
**A28**	$[C_{15}H_{20}F_2N_2O_4+H]^+$	331.1464	331.1465
**A29**	$[C_{15}H_{20}Cl_2N_2O_4+H]^+$	363.0873	363.0869
**A30**	$[C_{15}H_{20}Cl_2N_2O_4+H]^+$	363.0873	363.0874
**A31**	$[C_{15}H_{20}Cl_2N_2O_5+H]^+$	379.0822	379.0821
**A32**	$[C_{17}H_{20}F_6N_2O_4+H]^+$	431.1400	431.1406
**A33**	$[C_{15}H_{21}ClN_2O_5+H]^+$	345.1212	345.1217
**A34**	$[C_{19}H_{26}N_2O_6+H]^+$	379.1864	379.1859
**A35**	$[C_{15}H_{22}N_2O_6+H]^+$	327.1551	327.1554
**A36**	$[C_{16}H_{24}N_2O_6+H]^+$	341.1707	341.1705
**A37**	$[C_{24}H_{36}N_2O_5+H]^+$	433.2697	433.2697
**A38**	$[C_{19}H_{24}N_2O_4+H]^+$	345.1809	345.1809
**A39**	$[C_{14}H_{19}BrN_2O4_{+}H]^+$	359.0601	359.0597

**A40**	$[C_{15}H_{22}N_2O_4+H]^+$	295.1652	295.1655
**A41**	$[C_{22}H_{26}N_2O_4+H]^+$	383.1965	383.1959
**A42**	$[C_{16}H_{22}N_2O_6+H]^+$	339.1551	339.1552
**A43**	$[C_{14}H_{24}N_2O_4+H]^+$	285.1809	285.1809
**A44**	$[C_{14}H_{24}N_2O_4+H]^+$	285.1809	285.1812
**A45**	$[C_{16}H_{24}N_2O_4+H]^+$	309.1809	309.1813
**A46**	$[C_{17}H_{21}F_3N_2O_4+H]^+$	375.1526	375.1526
**A47**	$[C_{16}H_{21}N_3O_6+H]^+$	352.1503	352.1499
**A48**	$[C_9H_{18}N_2O_4+H]^+$	219.1339	219.1339
**A49**	$[C_{23}H_{36}N_2O_4+H]^+$	405.2748	405.2755
**A50**	$[C_{17}H_{24}N_2O_4+H]^+$	321.1809	321.1810
**A51**	$[C_{15}H_{21}N_3O_6+H]^+$	340.1503	340.1500
**A52**	$[C_{14}H_{19}N_3O_6+H]^+$	326.1347	326.1352
**A53**	$[C_{14}H_{18}Cl_2N_2O_4+H]^+$	349.0716	349.0716
**A54**	$[C_{15}H_{18}ClF_3N_2O_4+H]^+$	383.0980	383.0977
**A55**	$[C_{28}H_{32}N_2O_4+H]^+$	461.2435	461.2434
**A56**	$[C_{17}H_{26}N_2O_5+H]^+$	339.1914	339.1910
**A57**	$[C_{16}H_{24}N_2O_4+H]^+$	309.1809	309.1805
**A58**	$[C_{17}H_{26}N_2O_4+H]^+$	323.1965	323.1965
**A59**	$[C_{15}H_{22}N_2O_4+H]^+$	295.1652	295.1650
**A60**	$[C_{14}H_{18}N_4O_8+H]^+$	371.1197	371.1200
**A61**	$[C_{14}H_{26}N_2O_4+H]^+$	287.1965	287.1970
**A62**	$[C_9H_{17}BrN_2O_4+H]^+$	297.0444	297.0438
**A63**	$[C_{15}H_{22}N_2O_5+H]^+$	311.1601	311.1597
**A64**	$[C_{18}H_{22}N_2O_5+H]^+$	347.1601	347.1595
**A65**	$[C_{18}H_{26}N_2O_6+H]^+$	367.1864	367.1866
**A66**	$[C_{15}H_{22}N_2O_5+H]^+$	311.1601	311.1600
**A67**	$[C_{19}H_{28}N_2O_7+H]^+$	397.1969	397.1969
**A68**	$[C_{15}H_{28}N_2O_4+H]^+$	301.2122	301.2121
**A69**	$[C_{19}H_{36}N_2O_5+H]^+$	373.2697	373.2691
**A70**	$[C_{15}H_{22}N_2O_5+H]^+$	311.1601	311.1605
**A71**	$[C_{18}H_{22}N_2O_6+H]^+$	363.1551	363.1547
**A72**	$[C_{20}H_{30}N_2O_4+H]^+$	363.2278	363.2277
**A73**	$[C_{16}H_{24}N_2O_6+H]^+$	341.1707	341.1715
**A74**	$[C_{15}H_{21}N_3O_6+H]^+$	340.1503	340.1495
**A75**	$[C_{16}H_{21}N_3O_6+H]^+$	352.1503	352.1506
**A76**	$[C_{18}H_{22}N_2O_4+H]^+$	331.1652	331.1673
**A77**	$[C_{19}H_{28}N_2O_7+H]^+$	397.1969	397.1975
**A78**	$[C_{18}H_{26}N_2O_6+H]^+$	367.1864	367.1864
**A79**	$[C_{10}H_{20}N_2O_5+H]^+$	249.1445	249.1439
**A80**	$[C_{15}H_{20}N_4O_8+H]^+$	385.1354	385.1355

**A81**	$[C_{18}H_{22}N_2O_4+H]^+$	331.1652	331.1650
**A82**	$[C_{14}H_{18}Br_2N_2O_5+H]^+$	452.9655	452.9655
**A83**	$[C_{17}H_{23}BrN_2O_5+H]^+$	415.0863	415.0867
**A84**	$[C_{15}H_{21}BrN_2O_4+H]^+$	373.0757	373.0750
**A85**	$[C_{16}H_{24}N_2O_6+H]^+$	341.1707	341.1704
**A86**	$[C_{21}H_{22}N_2O_5+H]^+$	383.1601	383.1597
**A87**	$[C_{18}H_{22}N_2O_5+H]^+$	347.1601	347.1604
**A88**	$[C_{17}H_{24}N_2O_5+H]^+$	337.1758	337.1764
**A89**	$[C_{17}H_{24}N_2O_4+H]^+$	321.1809	321.1803
**A90**	$[C_{15}H_{22}N_2O_6+H]^+$	327.1551	327.1551
**A91**	$[C_{15}H_{21}BrN_2O_4+H]^+$	373.0757	373.0755
**A92**	$[C_{17}H_{24}N_2O_4+H]^+$	321.1809	321.1812
**A93**	$[C_{20}H_{24}N_2O_5+H]^+$	373.1758	373.1766
**A94**	$[C_{22}H_{22}N_2O_6+H]^+$	411.1551	411.1550
**A95**	$[C_{15}H_{20}F_2N_2O_4+H]^+$	331.1464	331.1459
**A96**	$[C_{24}H_{24}N_2O_4+H]^+$	405.1809	405.1805
**A97**	$[C_{10}H_{19}BrN_2O_4+H]^+$	311.0601	311.0601
**A98**	$[C_{14}H_{19}BrN_2O_4+H]^+$	359.0601	359.0600
**A99**	$[C_{17}H_{24}N_2O_5+H]^+$	337.1758	337.1764
**A100**	$[C_{14}H_{19}BrN_2O_4+H]^+$	359.0601	359.0599
**A101**	$[C_{16}H_{24}N_2O_5+H]^+$	325.1758	325.1753
**A102**	$[C_{16}H_{24}N_2O_4+H]^+$	309.1809	309.1809
**A103**	$[C_{19}H_{28}N_2O_4+H]^+$	349.2122	349.2129
**A104**	$[C_{15}H_{21}BrN_2O_5+H]^+$	389.0707	389.0711
**A105**	$[C_{11}H_{20}N_2O_4+H]^+$	245.1496	245.1500
**A106**	$[C_{11}H_{22}N_2O_5+H]^+$	263.1601	263.1597
**A107**	$[C_{18}H_{28}N_2O_7+H]^+$	385.1969	385.1974
**A108**	$[C_{17}H_{26}N_2O_6+H]^+$	355.1864	355.1868
**A109**	$[C_{10}H_{20}N_2O_4S+H]^+$	265.1217	265.1217
**A110**	$[C_{18}H_{22}N_2O_6+H]^+$	363.1551	363.1560
**A111**	$[C_{14}H_{26}N_2O_4+H]^+$	287.1965	287.1959
**A112**	$[C_{15}H_{22}N_2O_4+H]^+$	295.1652	295.1652
**A113**	$[C_{15}H_{21}BrN_2O_4+H]^+$	373.0757	373.0754
**A114**	$[C_{17}H_{24}N_2O_6+H]^+$	353.1707	353.1709
**A115**	$[C_{15}H_{21}BrN_2O_4+H]^+$	373.0757	373.0755
**A116**	$[C_{15}H_{22}N_2O_5+H]^+$	311.1601	311.1601
**A117**	$[C_{16}H_{21}BrN_2O_4+H]^+$	385.0757	385.0765
**A118**	$[C_{16}H_{21}BrN_2O_4+H]^+$	385.0757	385.0764
**A119**	$[C_{22}H_{24}N_2O_4+H]^+$	381.1809	381.1809
**A120**	$[C_{21}H_{38}N_2O_4+H]^+$	383.2904	383.2902
**A121**	$[C_{17}H_{28}N_2O_5+H]^+$	341.2071	341.2077

**A122**	$[C_{20}H_{24}N_2O_4+H]^+$	357.1809	357.1816
**A123**	$[C_{21}H_{22}N_2O_5+H]^+$	383.1601	383.1599
**A124**	$[C_{14}H_{19}Br_2N_3O_4+H]^+$	451.9815	451.9820
**A125**	$[C_{13}H_{26}N_2O_4+H]^+$	275.1965	275.1966
**A126**	$[C_{15}H_{22}N_2O_6+H]^+$	327.1551	327.1547
**A127**	$[C_{15}H_{20}F_2N_2O_4+H]^+$	331.1464	331.1461
**A128**	$[C_{27}H_{30}N_2O_4+H]^+$	447.2278	447.2267
**A129**	$[C_{21}H_{24}N_2O_4+H]^+$	369.1809	369.1800
**A130**	$[C_{16}H_{24}N_2O_4+H]^+$	309.1809	309.1809
**A131**	$[C_{16}H_{24}N_2O_5+H]^+$	325.1758	325.1769
**A132**	$[C_{21}H_{24}N_2O_4+H]^+$	369.1809	369.1816
**A133**	$[C_{15}H_{26}N_2O_4S_2+H]^+$	363.1407	363.1398
**A134**	$[C_{10}H_{20}N_2O_4+H]^+$	233.1496	233.1505
**B1**	$[C_{14}H_{26}N_2O_8+H]^+$	351.1762	351.1756
**B2**	$[C_{14}H_{36}N_2O_5+H]^+$	373.2697	373.2703
**B3**	$[C_{12}H_{22}N_2O_5+H]^+$	275.1601	275.1599
**B4**	$[C_{12}H_{22}N_2O_4+H]^+$	259.1652	259.1655
**B5**	$[C_{12}H_{22}N_2O_4+H]^+$	259.1652	259.1647
**B6**	$[C_{13}H_{24}N_2O_4+H]^+$	273.1809	273.1811
**B7**	$[C_{14}H_{26}N_2O_4+H]^+$	287.1965	287.1960
**B8**	$[C_{15}H_{28}N_2O_4+H]^+$	301.2122	301.2127
**B9**	$[C_{15}H_{28}N_2O_4+H]^+$	301.2122	301.2122
**B10**	$[C_{15}H_{28}N_2O_5+H]^+$	317.2071	317.2070
**B11**	$[C_{19}H_{32}N_2O_4+H]^+$	353.2435	353.2429
**B12**	$[C_{16}H_{24}N_2O_4S+H]^+$	341.1530	341.1532
**B13**	$[C_{16}H_{22}N_2O_4+H]^+$	307.1652	307.1650
**B14**	$[C_{18}H_{26}N_2O_4+H]^+$	335.1965	335.1966
**B15**	$[C_{16}H_{21}F_3N_2O_4+H]^+$	363.1526	363.1524
**B16**	$[C_{16}H_{24}N_2O_5+H]^+$	325.1758	325.1756
**B17**	$[C_{16}H_{21}F_3N_2O_4+H]^+$	363.1526	363.1523
**B18**	$[C_{15}H_{22}N_2O_5+H]^+$	311.1601	311.1603
**B19**	$[C_{16}H_{24}N_2O_5+H]^+$	325.1758	325.1757
**B20**	$[C_{15}H_{23}N_3O_4+H]^+$	310.1761	310.1755
**B21**	$[C_{15}H_{21}FN_2O_4+H]^+$	313.1558	351.1755
**B22**	$[C_{16}H_{21}F_3N_2O_4+H]^+$	363.1526	363.1530
**B23**	$[C_{17}H_{23}ClN_2O_5+H]^+$	371.1368	371.1366
**B24**	$[C_{16}H_{24}N_2O_5+H]^+$	325.1758	325.1752
**B25**	$[C_{15}H_{22}N_2O_5+H]^+$	311.1601	311.1600
**B26**	$[C_{15}H_{23}N_3O_4+H]^+$	310.1761	310.1765
**B27**	$[C_{15}H_{20}F_2N_2O_4+H]^+$	331.1464	331.1464
**B28**	$[C_{15}H_{20}F_2N_2O_4+H]^+$	331.1464	331.1465

**B29**	$[C_{15}H_{20}Cl_2N_2O_4+H]^+$	363.0873	363.0872
**B30**	$[C_{15}H_{20}Cl_2N_2O_4+H]^+$	363.0873	363.0876
**B31**	$[C_{15}H_{20}Cl_2N_2O_5+H]^+$	379.0822	379.0817
**B32**	$[C_{17}H_{20}F_6N_2O_4+H]^+$	431.1400	431.1403
**B33**	$[C_{15}H_{21}ClN_2O_5+H]^+$	345.1212	345.1211
**B34**	$[C_{19}H_{26}N_2O_6+H]^+$	379.1864	379.1859
**B35**	$[C_{15}H_{22}N_2O_6+H]^+$	327.1551	327.1549
**B36**	$[C_{16}H_{24}N_2O_6+H]^+$	341.1707	341.1702
**B37**	$[C_{24}H_{36}N_2O_5+H]^+$	433.2697	433.2701
**B38**	$[C_{19}H_{24}N_2O_4+H]^+$	345.1809	345.1815
**B39**	$[C_{14}H_{19}BrN_2O4_{+}H]^+$	359.0601	359.0602
**B40**	$[C_{15}H_{22}N_2O_4+H]^+$	295.1652	295.1657
**B41**	$[C_{22}H_{26}N_2O_4+H]^+$	383.1965	383.1962
**B42**	$[C_{16}H_{22}N_2O_6+H]^+$	339.1551	339.1551
**B43**	$[C_{14}H_{24}N_2O_4+H]^+$	285.1809	285.1815
**B44**	$[C_{14}H_{24}N_2O_4+H]^+$	285.1809	285.1811
**B45**	$[C_{16}H_{24}N_2O_4+H]^+$	309.1809	309.1804
**B46**	$[C_{17}H_{21}F_3N_2O_4+H]^+$	375.1526	375.1525
**B47**	$[C_{16}H_{21}N_3O_6+H]^+$	352.1503	352.1508
**B48**	$[C_9H_{18}N_2O_4+H]^+$	219.1339	219.1336
**B49**	$[C_{23}H_{36}N_2O_4+H]^+$	405.2748	405.2746
**B50**	$[C_{17}H_{24}N_2O_4+H]^+$	321.1809	321.1809
**B51**	$[C_{15}H_{21}N_3O_6+H]^+$	340.1503	340.1501
**B52**	$[C_{14}H_{19}N_3O_6+H]^+$	326.1347	326.1350
**B53**	$[C_{14}H_{18}Cl_2N_2O_4+H]^+$	349.0716	349.072
**B54**	$[C_{15}H_{18}ClF_3N_2O_4+H]^+$	383.0980	383.0986
**B55**	$[C_{28}H_{32}N_2O_4+H]^+$	461.2435	461.2430
**B56**	$[C_{17}H_{26}N_2O_5+H]^+$	339.1914	339.1909
**B57**	$[C_{16}H_{24}N_2O_4+H]^+$	309.1809	309.1801
**B58**	$[C_{17}H_{26}N_2O_4+H]^+$	323.1965	323.1972
**B59**	$[C_{15}H_{22}N_2O_4+H]^+$	295.1652	295.1649
**B60**	$[C_{14}H_{18}N_4O_8+H]^+$	371.1197	371.1197
**B61**	$[C_{14}H_{26}N_2O_4+H]^+$	287.1965	287.1966
**B62**	$[C_9H_{17}BrN_2O_4+H]^+$	297.0444	297.044
**B63**	$[C_{15}H_{22}N_2O_5+H]^+$	311.1601	311.1605
**B64**	$[C_{18}H_{22}N_2O_5+H]^+$	347.1601	347.1604
**B65**	$[C_{18}H_{26}N_2O_6+H]^+$	367.1864	367.1867
**B66**	$[C_{15}H_{22}N_2O_5+H]^+$	311.1601	311.1595
**B67**	$[C_{19}H_{28}N_2O_7+H]^+$	397.1969	397.1962
**B68**	$[C_{15}H_{28}N_2O_4+H]^+$	301.2122	301.2120
**B69**	$[C_{19}H_{36}N_2O_5+H]^+$	373.2697	373.2696

**B70**	$[C_{15}H_{22}N_2O_5+H]^+$	311.1601	311.1601
**B71**	$[C_{18}H_{22}N_2O_6+H]^+$	363.1551	363.1556
**B72**	$[C_{20}H_{30}N_2O_4+H]^+$	363.2278	363.2274
**B73**	$[C_{16}H_{24}N_2O_6+H]^+$	341.1707	341.1710
**B74**	$[C_{15}H_{21}N_3O_6+H]^+$	340.1503	340.1498
**B75**	$[C_{16}H_{21}N_3O_6+H]^+$	352.1503	352.1507
**B76**	$[C_{18}H_{22}N_2O_4+H]^+$	331.1652	331.1663
**B77**	$[C_{19}H_{28}N_2O_7+H]^+$	397.1969	397.1967
**B78**	$[C_{18}H_{26}N_2O_6+H]^+$	367.1864	367.1866
**B79**	$[C_{10}H_{20}N_2O_5+H]^+$	249.1445	249.1436
**B80**	$[C_{15}H_{20}N_4O_8+H]^+$	385.1354	385.1349
**B81**	$[C_{18}H_{22}N_2O_4+H]^+$	331.1652	331.1652
**B82**	$[C_{14}H_{18}Br_2N_2O_5+H]^+$	452.9655	452.9653
**B83**	$[C_{17}H_{23}BrN_2O_5+H]^+$	415.0863	415.0865
**B84**	$[C_{15}H_{21}BrN_2O_4+H]^+$	373.0757	373.0757
**B85**	$[C_{16}H_{24}N_2O_6+H]^+$	341.1707	341.1708
**B86**	$[C_{21}H_{22}N_2O_5+H]^+$	383.1601	383.1609
**B87**	$[C_{18}H_{22}N_2O_5+H]^+$	347.1601	347.1599
**B88**	$[C_{17}H_{24}N_2O_5+H]^+$	337.1758	337.1760
**B89**	$[C_{17}H_{24}N_2O_4+H]^+$	321.1809	321.1812
**B90**	$[C_{15}H_{22}N_2O_6+H]^+$	327.1551	327.1555
**B91**	$[C_{15}H_{21}BrN_2O_4+H]^+$	373.0757	373.0734
**B92**	$[C_{17}H_{24}N_2O_4+H]^+$	321.1809	321.1818
**B93**	$[C_{20}H_{24}N_2O_5+H]^+$	373.1758	373.1755
**B94**	$[C_{22}H_{22}N_2O_6+H]^+$	411.1551	411.1545
**B95**	$[C_{15}H_{20}F_2N_2O_4+H]^+$	331.1464	331.1464
**B96**	$[C_{24}H_{24}N_2O_4+H]^+$	405.1809	405.1808
**B97**	$[C_{10}H_{19}BrN_2O_4+H]^+$	311.0601	311.0600
**B98**	$[C_{14}H_{19}BrN_2O_4+H]^+$	359.0601	359.0595
**B99**	$[C_{17}H_{24}N_2O_5+H]^+$	337.1758	337.1765
**B100**	$[C_{14}H_{19}BrN_2O_4+H]^+$	359.0601	359.059
**B101**	$[C_{16}H_{24}N_2O_5+H]^+$	325.1758	325.1757
**B102**	$[C_{16}H_{24}N_2O_4+H]^+$	309.1809	309.1805
**B103**	$[C_{19}H_{28}N_2O_4+H]^+$	349.2122	349.2130
**B104**	$[C_{15}H_{21}BrN_2O_5+H]^+$	389.0707	389.0714
**B105**	$[C_{11}H_{20}N_2O_4+H]^+$	245.1496	245.1501
**B106**	$[C_{11}H_{22}N_2O_5+H]^+$	263.1601	263.1607
**B107**	$[C_{18}H_{28}N_2O_7+H]^+$	385.1969	385.1975
**B108**	$[C_{17}H_{26}N_2O_6+H]^+$	355.1864	355.1864
**B109**	$[C_{10}H_{20}N_2O_4S+H]^+$	265.1217	265.1209
**B110**	$[C_{18}H_{22}N_2O_6+H]^+$	363.1551	363.1555

**B111**	$[C_{14}H_{26}N_2O_4+H]^+$	287.1965	287.1969
**B112**	$[C_{15}H_{22}N_2O_4+H]^+$	295.1652	295.1660
**B113**	$[C_{15}H_{21}BrN_2O_4+H]^+$	373.0757	373.0750
**B114**	$[C_{17}H_{24}N_2O_6+H]^+$	353.1707	353.1715
**B115**	$[C_{15}H_{21}BrN_2O_4+H]^+$	373.0757	373.0767
**B116**	$[C_{15}H_{22}N_2O_5+H]^+$	311.1601	311.159
**B117**	$[C_{16}H_{21}BrN_2O_4+H]^+$	385.0757	385.0765
**B118**	$[C_{16}H_{21}BrN_2O_4+H]^+$	385.0757	385.0761
**B119**	$[C_{22}H_{24}N_2O_4+H]^+$	381.1809	381.1811
**B120**	$[C_{21}H_{38}N_2O_4+H]^+$	383.2904	383.2898
**B121**	$[C_{17}H_{28}N_2O_5+H]^+$	341.2071	341.2071
**B122**	$[C_{20}H_{24}N_2O_4+H]^+$	357.1809	357.1815
**B123**	$[C_{21}H_{22}N_2O_5+H]^+$	383.1601	383.1595
**B124**	$[C_{14}H_{19}Br_2N_3O_4+H]^+$	451.9815	451.9811
**B125**	$[C_{13}H_{26}N_2O_4+H]^+$	275.1965	275.1962
**B126**	$[C_{15}H_{22}N_2O_6+H]^+$	327.1551	327.1548
**B127**	$[C_{15}H_{20}F_2N_2O_4+H]^+$	331.1464	331.1460
**B128**	$[C_{27}H_{30}N_2O_4+H]^+$	447.2278	447.2277
**B129**	$[C_{21}H_{24}N_2O_4+H]^+$	369.1809	369.1805
**B130**	$[C_{16}H_{24}N_2O_4+H]^+$	309.1809	309.1809
**B131**	$[C_{16}H_{24}N_2O_5+H]^+$	325.1758	325.1750
**B132**	$[C_{21}H_{24}N_2O_4+H]^+$	369.1809	369.1817
**B133**	$[C_{15}H_{26}N_2O_4S_2+H]^+$	363.1407	363.1403
**B134**	$[C_{10}H_{20}N_2O_4+H]^+$	233.1496	233.1499

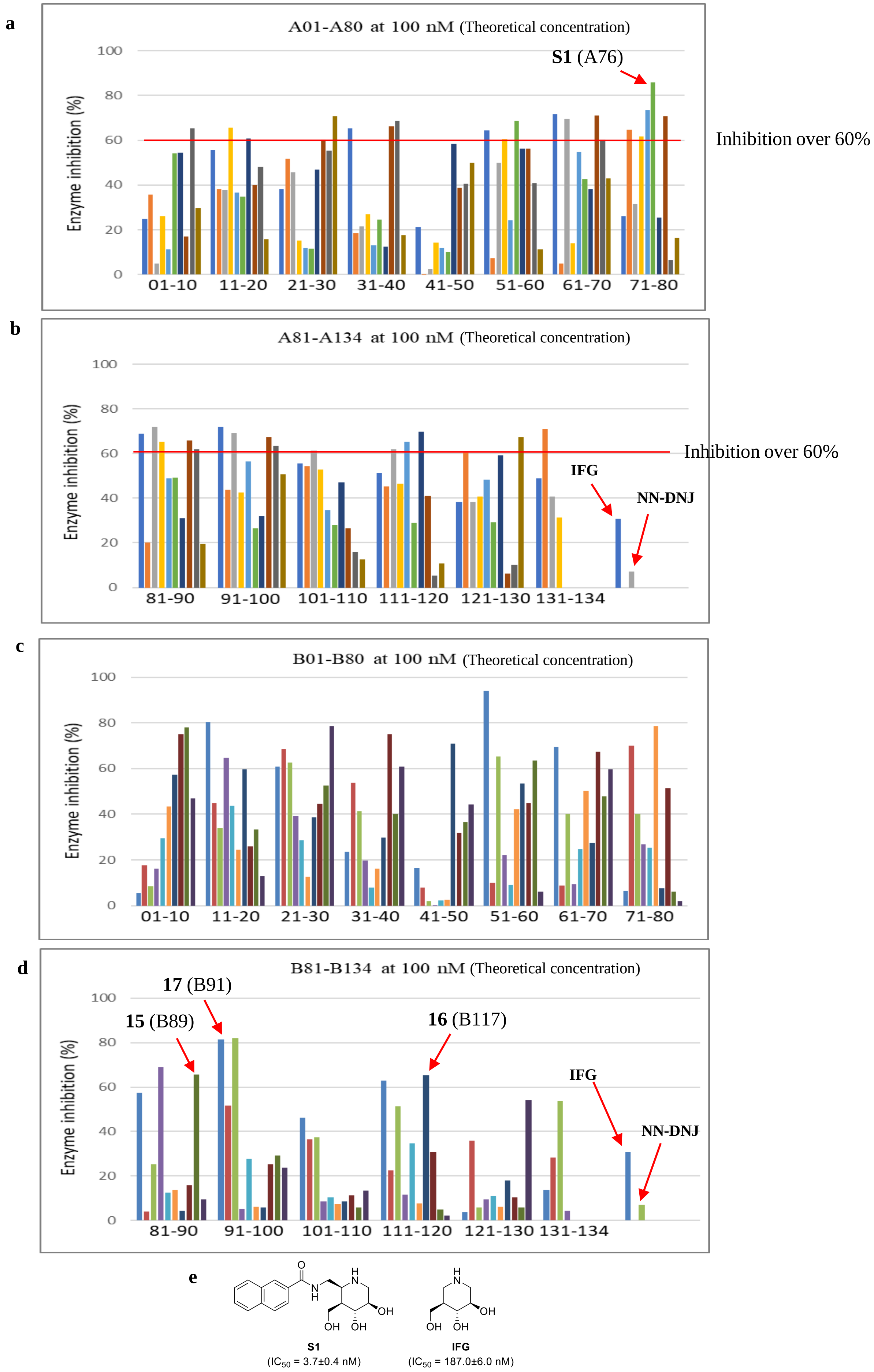

Supplementary Figure 4. Inhibition percentages of (**a**) A1-A80, (**b**) A81-A134, (**c**) B1-B80, and (**d**)

B81-B134 against rh-GCase at a theoretical concentration of 100 nM (*in-situ* enzyme-based screening). (**e**) IC_{50} values of resynthesized compounds **S1**, and IFG against rh-GCase. IFG and NN-DNJ were also tested as reference compounds at a concentration of 100 nM.

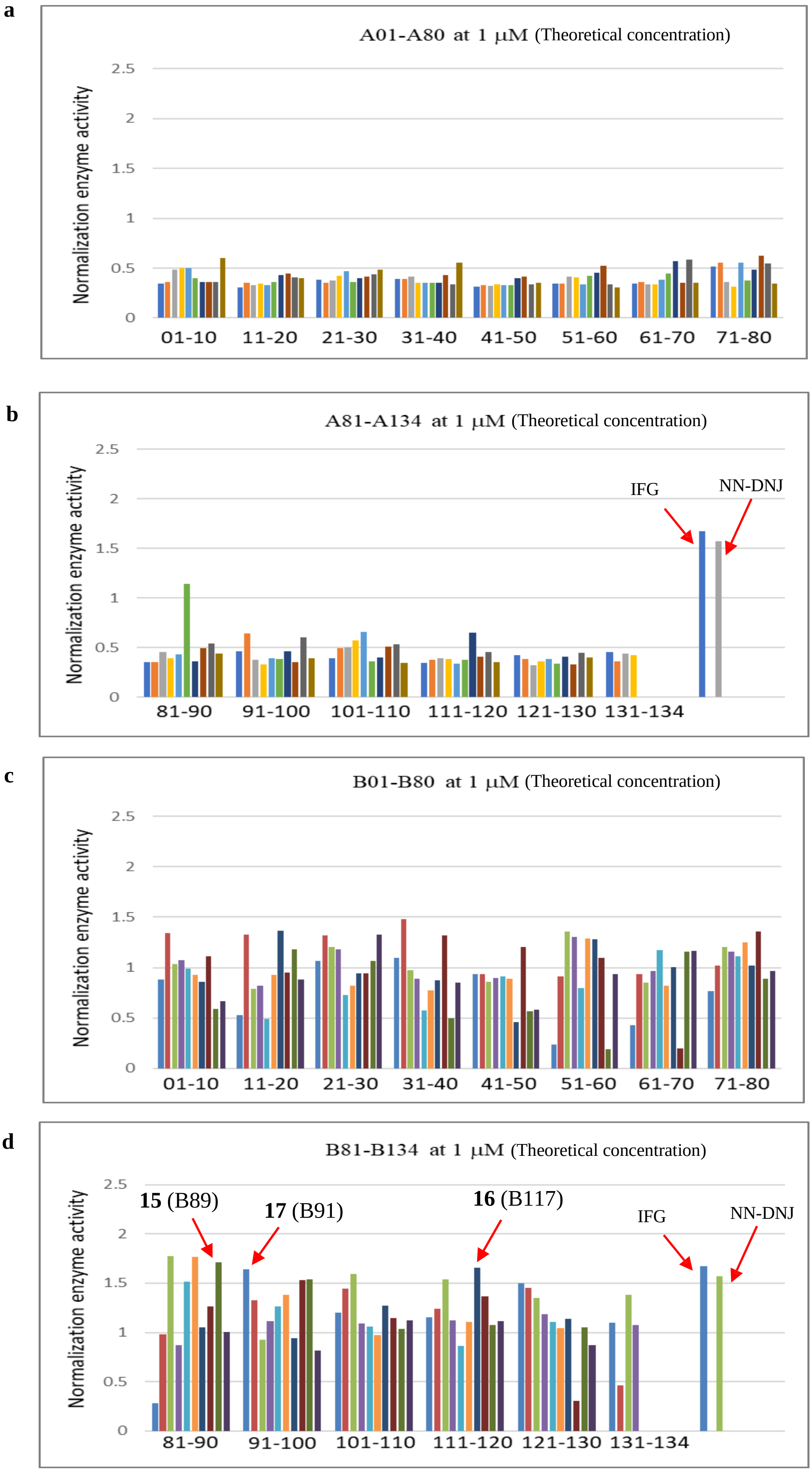

Supplementary Figure 5. The influence of (**a**) A1-A80, (**b**) A81-A134, (**c**) B1-B80, and (**d**) A81-A134 at a theoretical concentration of 1 μM on cellular β-glucosidase activities in N370S Gaucher fibroblasts (GM00372) (*in-situ* cell-based chaperone screening). Enzyme activity is normalized to untreated cells, assigned a relative activity of 1. IFG and NN-DNJ were also tested as

reference compounds at a concentration of 1 µM.

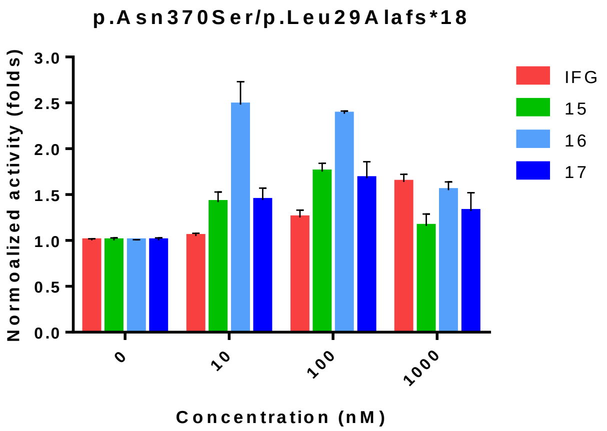

Supplementary Figure 6. The influence of **15**, **16**, **17**, and IFG (0–1000 nM) on cellular β-glucosidase activities in p.Asn370Ser/p.Leu29Alafs*18 Gaucher fibroblasts (GM00372). Enzyme activity is normalized to untreated cells, assigned a relative activity of 1. Mean values ± SD are shown for triplicate experiments.

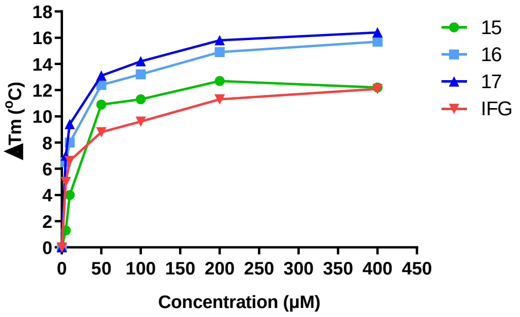

Supplementary Figure 7. Thermal shift study of IFG, 15, 16, and 17 toward rh-GCase at different concentrations (from 0-400 µM).

Supplementary Methods

Abbreviations

(1) IFG: Isofagomine, (2) GCase: β-glucocerebrosidase, (3) CC: column chromatography, (4) rt: room temperature, (5) *NN*-DNJ: *N*-(*n*-Nonyl)-1-deoxynojirimycin, (6) SD: standard deviation

General information

All chemicals were obtained from commercial suppliers and used without further purification. NMR spectra were recorded on dilute solutions in $CDCl_3$, CD_3OD, and D_2O on Bruker AVANCE 600 and AMX 400 spectrometers at ambient temperature. Chemical shifts are reported in units of parts per million (ppm, δ), and coupling constants (J) are given in Hz. The splitting patterns are reported as s (singlet), br s (broad singlet), d (doublet), t (triplet), pt (pseudo-triplet), q (quartet), pq (pseudo-quartet), m (multiplet), dd (doublet of doublets), and td (triplet of doublets). Ozonolysis was performed by an ozone generator (Fischer Tech. OZ 502/10). High-resolution ESI mass spectra were recorded on a Bruker Daltonics spectrometer. CC refers to column chromatography. Concentration refers to rotary evaporation. 4-Methylumbelliferyl β-D-glucopyranoside was obtained commercially from Sigma-Aldrich, and GCase was Human β-glucocerebrosidase (Ceredase) from Genzyme. Fibroblasts derived from Gaucher patients (GM00372 and GM00877) were purchased from Coriell Institute (Camden, NJ).

Experimental data

Diethyl (2*S*,3*S*)-2,3-bis(benzyloxy)succinate (2)

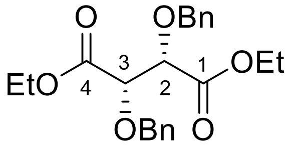

A mixture of BnBr (34.8 mL, 290 mmol) and NaH (11.6 g, 290 mmol) in DMF (400 mL) was stirred at -20 °C for 30 min. Subsequently, (–)-diethyl D-tartrate (30 g, 145 mmol) in DMF (100 mL) was added dropwise to the mixture and stirred for 15 min and then warmed to 0 °C for 1 h. The reaction was quenched by water and extracted with ether. The organic layers were washed with brine, dried over $MgSO_4$, concentrated, and purified by CC to give **2** as a colorless oil (46.3 g, 120 mmol, 83%). TLC (Hexanes/EtOAc = 5/1, v/v) R_f = 0.5; [α]D25 = -108.4° (c = 0.1 in CH_2Cl_2); ^{1}HNMR (600 MHz, $CDCl_3$) δ 7.28–7.23 (m, 10H, Ph-H), 4.85 (d, J = 12.0 Hz, 2H, Ph*CH*$_2$), 4.45 (d, J = 12.0 Hz, 2H, Ph*CH*$_2$), 4.40 (s, 2H, H-2 and H-3), 4.20–4.15 (m, 2H, Me*CH*$_2$), 4.09–4.03 (m, 2H, Me*CH*$_2$), 1.16 (t, J = 7.3 Hz, 6H, CH_3); ^{13}C NMR (150 MHz, $CDCl_3$) δ 168.8 (C-2 and C-5), 136.8 (Ph-C), 127.99 (Ph-C), 127.95 (Ph-C), 127.6 (Ph-C), 78.3 (C-2 and C-3), 72.9 (Ph*CH*$_2$), 60.9 (Me*CH*$_2$), 13.8 (CH_3); HRMS: calculated for $[C_{22}H_{26}O_6+Na]^+$ 409.1622, found

409.1656.

((2*R*,3*R*)-2,3-Bis(benzyloxy)-4-((*tert*-butyldiphenylsilyl)oxy)butan-1-ol) (3)

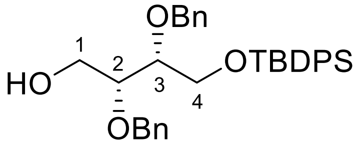

Compound **2** (28.8 g, 74.5 mmol) in THF was added slowly to a suspension of $LiAlH_4$ (5.37 g, 141.6 mmol) in dry THF (99 mL) at 0 °C for 1 h. The reaction was quenched by water and 4N $KOH_{(aq)}$ at 0 °C. The mixture was filtered through Celite and the filtrate was dried over $MgSO_4$ and concentrated to give the crude compound as a colorless oil (19.7 g, 65.2 mmol); TLC (Hexanes/EtOAc = 1/2, v/v) R_f = 0.5. A mixture of NaH (2.6 g, 65.2 mmol) and crude compound (19.7 g, 65.2 mmol) in THF (99 mL) was stirred at 0 ^{o}C for 30 min, then the solution of TBDPSCl (17 mL, 65.2 mmol) in THF (33 mL) was added dropwise to the mixture for 1 h. The reaction was quenched with water and extracted with EtOAc. The organic layers were washed with brine, dried over $MgSO_4$, concentrated and purified by CC to give **3** as white solid (30 g, 55.5 mmol, 75%); TLC (Hexanes/EtOAc = 5/1, v/v) R_f = 0.4; [α]D25 = -8.2^o (*c* = 0.1 in CH_2Cl_2); ^{1}H NMR (600 MHz, $CDCl_3$) δ 7.80–7.67 (m, 4H, Ph-H), 7.51–7.33 (m, 16H, Ph-H), 4.74–4.59 (m, 4H, Ph*CH_2*), 4.01–3.94 (m, 2H, H-4), 3.88–3.82 (m, 2H, H-1), 3.79–3.74 (m, 2H, H-2 and H-3), 1.17 (s, 9H, CH_3); ^{13}C NMR (150 MHz, $CDCl_3$) δ 138.2 (Ph-C), 133.5 (Ph-C), 133.0 (Ph-C), 129.7 (Ph-C), 128.3 (Ph-C), 128.2 (Ph-C), 127.8 (Ph-C), 127.7 (Ph-C), 127.6 (Ph-C), 79.8 (C-3), 79.0 (C-2), 72.8 (Ph*CH_2*), 72.7 (Ph*CH_2*), 62.8 (C-4), 61.6 (C-1), 26.7 (Si*C*$(CH_3)_3$), 19.1 (CH_3); HRMS: calculated for $[C_{34}H_{40}O_4Si+Na]^+$ 563.2588, found 563.2573.

(((2*R*,3*R*,*E*)-2,3-Bis(benzyloxy)-5-nitropent-4-en-1-yl)oxy)(*tert*-butyl)diphenylsilane (4)

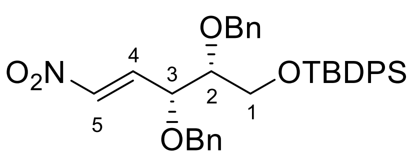

Compound **3** (40.7 g, 75 mmol) in CH_2Cl_2 (180 mL) was added dropwise to a mixture of DMSO (16.0 mL, 225 mmol) and oxalyl chloride (12.9 mL, 150 mol) in CH_2Cl_2 (90 mL) at -78 °C. After stirring for 30 min, Et_3N (52 mL, 375 mmol) was added to the mixture and warmed to room temperature. The mixture was quenched by water and extracted with CH_2Cl_2 and the organic layers were washed with brine, dried over $MgSO_4$, and concentrated to obtain a yellow oil; TLC (Hexanes/EtOAc = 5/1, v/v) R_f = 0.5; The yellow oil was added to a mixture of sodium methoxide

(27 mL, 150 mmol) and nitromethane (9.0 mL, 165 mmol) in dry methanol (180 mL) at 0 °C and warmed to room temperature for 1 h. The reaction was quenched with water and extracted with EtOAc. The organic layers were dried over $MgSO_4$, concentrated, and directly reacted with methanesulfonyl chloride (12.0 mL, 0.15 mol) in the presence of Et_3N (41.7 mL, 0.3 mol) at 0 °C and returned to rt for 1 h. The reaction was quenched with water and extracted with EtOAc. The organic layers were dried over $MgSO_4$, concentrated, and purified by CC to give **4** as a yellow oil (29.6 g, 0.051 mol, 3 steps 68%); TLC (Hexane/EtOAc = 7/1, v/v) R_f = 0.65; R_f = 0.3; [α]D25 = -3.2° (c = 0.1 in CH_2Cl_2); ^1H NMR (600 MHz, $CDCl_3$) δ 7.80–7.76 (m, 4H, Ph-H), 7.53–7.32 (m, 17H, Ph-H and H-5), 7.21 (dd, J = 1.7, 13.3 Hz, 1H, H-4), 4.75–4.55 (m, 4H, PhCH_2), 4.47 (m, 1H, H-3), 3.99 (dd, J = 4.9, 11.0 Hz, 1H, H-1), 3.88 (dd, J = 5.2, 11.0 Hz, 1H, H-1), 3.70 (pq, J = 5.2 Hz, 1H, H-2), 1.17 (s, 9H, CH_3); ^{13}C NMR (150 MHz, $CDCl_3$) δ 140.4 (C-4), 140.0 (C-5), 137.6 (Ph-C), 137.0 (Ph-C), 135.6 (Ph-C), 135.5 (Ph-C), 133.0 (Ph-C), 132.9 (Ph-C), 129.9 (Ph-C), 129.8 (Ph-C), 128.5 (Ph-C), 128.4 (Ph-C), 128.1 (Ph-C), 128.0 (Ph-C), 127.9 (Ph-C), 127.8 (Ph-C), 80.0 (C-2), 75.4 (C-3), 73.0 (PhCH_2), 72.7 (PhCH_2), 62.5 (C-1), 26.8 (Si$C(CH_3)_3$), 19.1 (CH_3); HRMS: calculated for $[C_{35}H_{39}NO_5Si+Na]^+$ 604.2490, found 604.2466.

(((2*R*,3*R*,4*R*)-2,3-Bis(benzyloxy)-4-(nitromethyl)hex-5-en-1-yl)oxy)(*tert*-butyl)diphenylsilane (5)

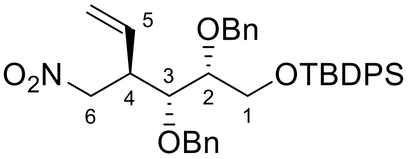

0.7M Vinylmagnesium bromide in THF (146 mL, 102 mmol) was added dropwise to a mixture of **4** (29.6 g, 51 mmol) in the presence of CuCl (1.51 g, 15.3 mmol) in THF (130 mL) at -78 °C for 1 h. The reaction was quenched by sat. $NH_4Cl_{(aq)}$ and filtrated through Celite. The filtrate was extracted with ether and the organic layers were dried over $MgSO_4$, concentrated and purified by CC to give **5** as a yellow oil (26.4 g, 43 mmol, 84%); TLC (Toluene) R_f = 0.65; R_f = 0.3; [α]D25 = -21.3° (c = 0.1 in CH_2Cl_2); ^1H NMR (600 MHz, $CDCl_3$) δ 7.72–7.67 (m, 4H, Ph-H), 7.47–7.21 (m, 16H, Ph-H), 5.67–5.61 (m, 1H, H-5), 5.11 (d, J = 10.5 Hz, 1H, CH=CH_2), 5.00 (d, J = 17.2 Hz, 1H, CH=CH_2), 4.76 (d, J = 11.4 Hz, 1H, PhCH_2), 4.60 (dd, J = 4.1, 12.3 Hz, 1H, H-6), 4.55 (m, , 2H, PhCH_2), 4.37 (d, J = 11.7 Hz, 1H, PhCH_2), 4.30 (dd, J = 10.0, 12.3 Hz, 1H, H-6), 3.88 (d, J = 5.7 Hz, 2H, H-1), 3.83 (dd, J = 3.4, 7.3 Hz, 1H, H-3), 3.57 (td, J = 3.4, 6.3 Hz, 1H, H-2), 3.29–3.30 (m, 1H, H-4), 1.09 (s, 9H, CH_3); ^{13}C NMR (150 MHz, $CDCl_3$) δ 137.9 (Ph-C), 137.7 (Ph-C), 135.52 (Ph-C), 135,49 (C-5), 134.4 (Ph-C), 133.0 (Ph-C), 132.9 (Ph-C), 129.9 (Ph-C), 129.8 (Ph-C), 128.4 (Ph-C), 128.3 (Ph-C), 128.03 (Ph-C), 127.95 (Ph-C), 127.9 (Ph-C), 127.8 (Ph-C), 127.7

(Ph-C), 127.6 (Ph-C), 119.5 (CH=*CH2*), 79.6 (C-2), 78.8 (C-3), 77.2 (C-6), 74.7 (Ph*CH2*), 72.7 (Ph*CH2*), 62.0 (C-1), 44.6 (C-4), 26.8 (Si*C*(CH_3)$_3$), 19.1 (CH_3); HRMS: calculated for $[C_{37}H_{43}NO_5Si+Na]^+$ 632.2803, found 632.2813.

(2*R*,3*R*,4*R*)-3,4-Bis(benzyloxy)-5-((*tert*-butyldiphenylsilyl)oxy)-2-vinylpentan-1-amine (6)

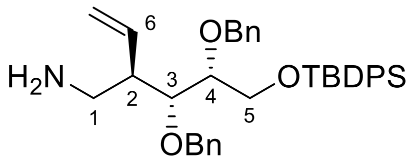

Compound **5** (10 g, 16.4 mmol) was added slowly to a suspension of $LiAlH_4$ (1.25 g, 32.8 mmol) in dry THF (80 mL) at 0 °C for 1 h. The reaction was quenched by water and 4N $KOH_{(aq)}$ at 0 °C. The mixture was filtered through Celite and the filtrate was dried over $MgSO_4$, concentrated and purified by CC to give compound **6** (8.1 g, 14.0 mmol, 85%); TLC (EtOAc/MeOH = 5/1, v/v) R_f = 0.3; [α]D25 = -15.3° (*c* = 0.1 in CH_2Cl_2); ^{1}H NMR (600 MHz, $CDCl_3$) δ 7.63–7.62 (m, 4H, Ph-H), 7.42–7.31 (m, 10H, Ph-H), 7.26–7.19 (m, 6H, Ph-H), 5.54 (m, 1H, H-6), 5.10 (dd, *J* = 1.6, 10.3 Hz, 1H, CH=*CH2*), 4.94 (dd, *J* = 1.6, 17.2 Hz, 1H, CH=*CH2*), 4.65 (d, *J* = 11.3 Hz, 1H, Ph*CH2*), 4.54 (d, *J* = 11.3 Hz, 1H, Ph*CH2*), 4.52 (d, *J* = 11.4 Hz, 1H, Ph*CH2*), 4.37 (d, *J* = 11.4 Hz, 1H, Ph*CH2*), 3.84–3.78 (m, 2H, H-5), 3.64 (dd, *J* = 3.2, 8.1 Hz, 1H, H-3), 3.57–3.55 (m, 1H, H-4), 2.95 (dd, *J* = 3.6, 12.4 Hz, 1H, H-1), 2.55–2.52 (m, 1H, H-1), 2.49–2.47 (m, 1H, H-2), 1.04 (s, 9H, CH_3); ^{13}C NMR (150 MHz, $CDCl_3$) δ 138.6 (Ph-C), 138.4 (Ph-C), 138.0 (Ph-C), 135.63 (Ph-C), 135.60 (C-6), 133.3 (Ph-C), 133.2 (Ph-C), 129.8 (Ph-C), 129.7 (Ph-C), 128.3 (Ph-C), 128.2 (Ph-C), 128.1 (Ph-C), 127.9 (Ph-C), 127.7 (Ph-C), 127.6 (Ph-C), 127.4 (Ph-C), 118.1 (CH=*CH2*), 80.2 (C-4), 80.1 (C-3), 74.4 (Ph*CH2*), 73.0 (Ph*CH2*), 62.8 (C-5), 49.8 (C-1), 42.4 (C-2), 26.9 (Si*C*(CH_3)$_3$), 19.2 (CH_3); HRMS: calculated for $[C_{37}H_{45}NO_3Si+H]^+$ 580.3241, found 580.3245.

(2*S*,3*R*,4*R*)-3,4-Bis(benzyloxy)-5-((*tert*-butyldiphenylsilyl)oxy)-2-vinylpentanoic acid (8)

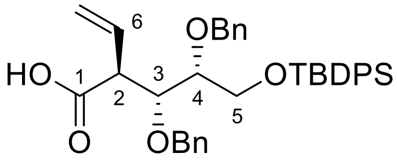

Compound **5** (10 g, 16.4 mmol) was reacted with sodium nitrite (4.5 g, 65.6 mmol) and acetic acid (11.8 mL, 197 mmol) in DMSO (50 mL). The mixture was stirred at 50 °C for 4 h and quenched by water. The mixture was extracted with ether and the organic layers were dried over $MgSO_4$, concentrated and purified by CC to give **8** as a yellow oil (6.2 g, 10.4 mmol, 63%); TLC

(Hexanes/EtOAc = 3/1, v/v) R_f = 0.3; [α]D25 = -17.3° (*c* = 0.1 in CH_2Cl_2); ^{1}H NMR (600 MHz, $CDCl_3$) *δ* 7.73–7.71 (m, 4H, Ph-H), 7.51–7.25 (m, 16H, Ph-H), 5.83–5.77 (m, 1H, H-6), 5.21 (d, *J* = 10.3 Hz, 1H, CH=*CH₂*), 5.19 (d, *J* = 17.2 Hz, 1H, CH=*CH₂*), 4.73 (dd, *J* = 11.0, 13.6 Hz, 2H, Ph*CH₂*), 4.58 (d, *J* = 11.8 Hz, 1H, Ph*CH₂*), 4.39 (d, *J* = 11.8 Hz, 1H, Ph*CH₂*), 4.20 (dd, *J* = 2.8, 8.9 Hz, 1H, H-3), 3.92 (d, *J* = 6.2 Hz, 2H, H-5), 3.67–3.62 (m, 2H, H-2 and H-4), 1.15 (s, 9H, CH_3); ^{13}C NMR (150 MHz, $CDCl_3$) *δ* 172.5 (C-1), 138.1 (Ph-C), 137.9 (Ph-C), 135.54 (Ph-C), 135.51 (Ph-C), 133.1 (Ph-C), 133.0 (Ph-C), 132.5 (C-6), 129.8 (Ph-C), 129.7 (Ph-C), 128.4 (Ph-C), 128.2 (Ph-C), 128.1 (Ph-C), 128.0 (Ph-C), 127.9 (Ph-C), 127.7 (Ph-C), 127.6 (Ph-C), 127.5 (Ph-C), 126.9 (Ph-C), 119.7 (CH=*CH₂*), 79.5 (C-4), 78.7 (C-3), 74.9 (Ph*CH₂*), 72.6 (Ph*CH₂*), 65.0 (C-5), 52.8 (C-2), 26.8 (Si*C*(CH_3)₃), 19.1 (CH_3); HRMS: calculated for $[C_{37}H_{42}O_5Si+Na]^+$ 617.2694, found 617.2655.

(2*R*,3*R*,4*R*)-3,4-Bis(benzyloxy)-5-((*tert*-butyldiphenylsilyl)oxy)-2-vinylpentan-1-ol (7)

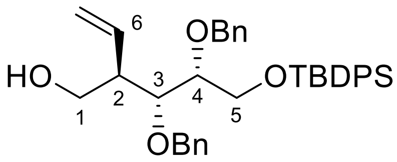

Compound **8** (6.2 g, 10.4 mmol) was reacted with potassium carbonate (4.3 g, 31.2 mmol) and iodomethane (84 μL, 13.5 mmol) in DMF (50 mL). The mixture was stirred for 1 h at rt and quenched with water. The mixture was extracted with ether and the organic layers were dried over $MgSO_4$ and concentrated to get crude residue. $LiAlH_4$ (0.79 g, 20.8 mmol) was added slowly to a solution of crude residue in dry THF (50 mL) at 0 °C and stirred at 0 °C for 1 h. The reaction was quenched by water and 4N $KOH_{(aq)}$ at 0 °C. The reaction mixture was filtered through Celite and the filtrate was dried over $MgSO_4$, concentrated and purified by CC to give **7** as a yellow oil (4.97 g, 8.6 mmol, 83%); TLC (Hexanes/EtOAc = 5/1, v/v) R_f = 0.5; [α]D25 = +9.9° (*c* = 0.1 in CH_2Cl_2); ^{1}H NMR (600 MHz, $CDCl_3$) *δ* 7.66–7.64 (m, 4H, Ph-H), 7.43–7.21 (m, 16H, Ph-H), 5.70–5.64 (m, 1H, H-6), 5.09 (d, *J* = 10.3 Hz, 1H, CH=*CH₂*), 5.05 (d, *J* = 17.4 Hz, 1H, CH=*CH₂*), 4.73 (d, *J* = 11.2 Hz, 1H, Ph*CH₂*), 4.58 (d, *J* = 11.2 Hz, 1H, Ph*CH₂*), 4.54 (d, *J* = 11.7 Hz, 1H, Ph*CH₂*), 4.39 (d, *J* = 11.7 Hz, 1H, Ph*CH₂*), 3.86–3.80 (m, 3H, H-3 and H-5), 3.71 (dd, *J* = 5.4, 11.0 Hz, 1H, H-1), 3.64–3.58 (m, 2H, H-1 and H-4), 2.69–2.64 (m, 1H, H-2), 1.06 (s, 9H, CH_3); ^{13}C NMR (150 MHz, $CDCl_3$) *δ* 138.4 (Ph-C), 138.1 (Ph-C), 137.1 (C-6), 135.7 (Ph-C), 135.5 (Ph-C), 133.2 (Ph-C), 133.16 (Ph-C), 129.9 (Ph-C), 129.7 (Ph-C), 128.5 (Ph-C), 128.3 (Ph-C), 128.28 (Ph-C), 128.1 (Ph-C), 128.09 (Ph-C), 128.0 (Ph-C), 127.9 (Ph-C), 127.7 (Ph-C), 127.4 (Ph-C), 117.9 (CH=*CH₂*), 80.7 (C-4), 80.3 (C-3), 75.1 (Ph*CH₂*), 73.0 (Ph*CH₂*), 63.8 (C-1), 62.7 (C-5), 47.4 (C-

2), 27.0 (Si*C*(CH_3)$_3$), 19.2 (CH_3); HRMS: calculated for $[C_{37}H_{44}O_4Si+Na]^+$ 603.2901, found 603.2956.

(2*R*,3*R*,4*R*)-2,3-Bis(benzyloxy)-4-((trityloxy)methyl)hex-5-en-1-ol (9)

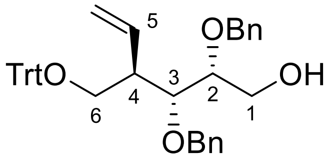

Compound **7** (4.97 g, 8.6 mmol) was treated with triphenylmethyl chloride (6.0 g, 21.5 mmol) in CH_2Cl_2 (50 mL) in the presence of triethylamine (6.0 mL, 43 mmol) and DMAP (cat.) at room temperature for 12 h. The reaction was quenched by water and extracted with CH_2Cl_2. The organic layers were dried over $MgSO_4$, concentrated, and directly reacted with tetrabutylammonium fluoride (17.2 mL, 17.2 mmol) in THF at 50 °C for 1 h. The reaction was quenched with water and extracted with EtOAc. The organic layers were dried over $MgSO_4$, concentrated, and purified by CC to give **9** as a colorless oil (4.27 g, 7.3 mmol, 2 steps, 85%); TLC (Hexanes/EtOAc = 5/1, v/v) R_f = 0.45; [α]D25 = -5.9^o (*c* = 0.1 in CH_2Cl_2); ^{1}H NMR (600 MHz, $CDCl_3$) *δ* 7.57–7.55 (m, 6H, Ph-H), 7.43–7.30 (m, 17H, Ph-H), 7.23–7.21 (m, 2H, Ph-H), 6.15–6.09 (m, 1H, H-5), 5.28–5.24 (m, 2H, CH=*CH*$_2$), 4.66 (m, 2H, Ph*CH*$_2$), 4.61 (d, *J* = 11.3 Hz, 1H, Ph*CH*$_2$), 4.50 (d, *J* = 11.3 Hz, 1H, Ph*CH*$_2$), 3.92 (dd, *J* = 4.1, 7.2 Hz, 1H, H-3), 3.84–3.82 (m, 1H, H-2), 3.79–3.76 (m, 2H, H-1), 3.54 (dd, *J* = 5.4, 9.0 Hz, 1H, H-6), 3.47 (dd, *J* = 5.4, 9.0 Hz, 1H, H-6), 2.97–2.94 (m, 1H, H-4); ^{13}C NMR (150 MHz, $CDCl_3$) *δ* 144.1 (Ph-C), 138.6 (Ph-C), 138.4 (Ph-C), 138.2 (C-5), 128.9 (Ph-C), 128.4 (Ph-C), 128.3 (Ph-C), 128.1 (Ph-C), 128.0 (Ph-C), 127.8 (Ph-C), 127.7 (Ph-C), 127.6 (Ph-C), 127.1 (Ph-C), 117.1 (CH=*CH*$_2$), 86.8 (*C*(Ph)$_3$), 80.1 (C-2), 80.0 (C-3), 73.8 (Ph*CH*$_2$), 72.9 (Ph*CH*$_2$), 63.8 (C-6), 62.0 (C-1), 46.6 (C-4); HRMS: calculated for $[C_{40}H_{40}O_4+Na]^+$ 607.2819, found 607.2783.

(3*R*,4*R*,5*R*)-3,4-Bis(benzyloxy)-5-((trityloxy)methyl)-2,3,4,5-tetrahydropyridine 1-oxide or (3*R*,4*R*,5*R*)-3,4-bis(benzyloxy)-5a-oxidoimino-5-(trityloxy)methyl isofagomine (1)

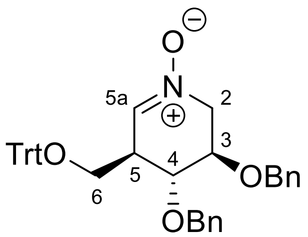

Compound **9** (4.27 g, 7.3 mmol) was treated with methanesulfonyl chloride (730 μL, 9.5 mmol) in

CH_2Cl_2 (20 mL) in the presence of triethylamine (1.5 mL, 11 mmol) at 0 °C for 30 min. The reaction was quenched by water and extracted with CH_2Cl_2. The organic layers were dried over $MgSO_4$, and concentrated. The resulting crude dissolved in CH_2Cl_2 (40 mL) was bubbled by a stream of O_3/O_2 below the surface of the solution at -78 °C. After the solution color became blue, the ozone generator was turned off and the solution was sparged with O_2 to purge the system for a few minutes and warmed to room temperature. The reaction solvent was removed and the residue was treated with hydroxylamine hydrochloride (2.0 g, 29.2 mmol) and triethylamine (8.1 mL, 58.4 mmol) in ethanol at 50 °C for 40 min. The reaction was quenched with water and extracted with EtOAc. The organic layers were dried over $MgSO_4$, concentrated, and purified by CC to give **1** (2.83 g, 4.85 mmol, 3 steps, 66%); TLC (EtOAc) R_f = 0.3; [α]D25 = +14.0° (*c* = 0.2 in CH_2Cl_2); ^{1}H NMR (600 MHz, $CDCl_3$) *δ* 7.43–7.10 (m, 26H, Ph-H and H-5a), 4.64 (d, *J* = 11.6 Hz, 1H, Ph*CH*$_2$), 4.48 (m, 2H, Ph*CH*$_2$), 4.44 (d, *J* = 11.6 Hz, 1H, Ph*CH*$_2$), 4.06–4.02 (m, 1H, H-2), 3.90–3.86 (m, 2H, H-2 and H-3), 3.72 (pt, *J* = 5.3 Hz, 1H, H-4), 3.36 (dd, *J* = 6.0, 9.2 Hz, 1H, H-6), 3.27 (dd, *J* = 6.6, 9.2 Hz, 1H, H-6), 2.80 (m, 1H, H-5); ^{13}C NMR (150 MHz, $CDCl_3$) *δ* 143.6 (Ph-C), 137.6 (Ph-C), 137.2 (Ph-C), 135.9 (C-5a), 128.7 (Ph-C), 128.6 (Ph-C), 128.3 (Ph-C), 128.2 (Ph-C), 128.1 (Ph-C), 127.9 (Ph-C), 127.8 (Ph-C), 127.4 (Ph-C), 87.2 (*C*(Ph)$_3$), 74.3 (C-3), 73.1 (C-4), 72.3 (Ph*CH*$_2$), 72.2 (Ph*CH*$_2$), 62.2 (C-6), 59.4 (C-2), 42.8 (C-5); HRMS: calculated for $[C_{39}H_{37}NO_4+H]^+$ 584.2795, found 584.2739.

(2*S*,3*R*,4*R*,5*R*)-4,5-Bis(benzyloxy)-1-hydroxy-3-((trityloxy)methyl)piperidine-2-carbonitrile or (3*R*,4*R*,5*R*,5a*R*)-3,4-bis(benzyloxy)-5a-cyano-1-*N*-hydroxy-5-(trityloxy)methyl isofagomine (10)

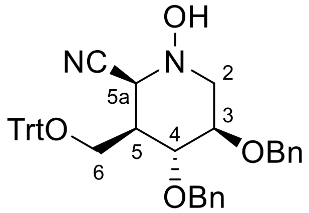

A mixture of **1** (1 g, 1.71 mmol) and trimethylsilyl cyanide (0.68 mL, 5.1 mmol) in dry methanol (10 mL) was stirred at 0 °C for 2 h. The reaction solvent was removed under vacuum and the residue was purified by CC to give **10** (0.93 g, 1.52 mmol, 89%) as a white solid; TLC (Hexanes/EtOAc = 3/1, v/v) R_f = 0.5; [α]D25 = +45.6° (*c* = 0.1 in CH_2Cl_2); ^{1}H NMR (600 MHz, $CDCl_3$) *δ* 7.38–7.17 (m, 23H, Ph-H), 6.97–6.96 (m, 2H, Ph-H), 5.46 (s, 1H, OH), 4.70 (d, *J* = 11.0 Hz, 1H, Ph*CH*$_2$), 4.68–4.50 (m, 3H, Ph*CH*$_2$ and H-5a), 4.28 (d, *J* = 11.0, 1H, Ph*CH*$_2$), 3.64 (dd, *J* = 4.1, 9.5 Hz, 1H, H-6), 3.61–3.57 (m, 1H, H-3), 3.42 (br s, 1H, H-4), 3.25 (br s, 1H, H-6), 3.12 (br s, 1H, H-2), 2.94 (m, 1H, H-2), 2.20–2.30 (m, 1H, H-5); ^{13}C NMR (150 MHz, $CDCl_3$) *δ* 1143.6

(Ph-C), 138.0 (Ph-C), 137.7 (Ph-C), 128.7 (Ph-C), 128.6 (Ph-C), 128.4 (Ph-C), 128.1 (Ph-C), 128.04 (Ph-C), 128.00 (Ph-C), 127.9 (Ph-C), 127.8 (Ph-C), 127.3 (Ph-C), 114.9 (C≡N), 87.3 (*C*(Ph)3), 78.8 (C-3), 77.9 (C-4), 75.2 (Ph*CH*2), 72.7 (Ph*CH*2), 61.3 (C-6), 59.9 (C-2), 56.8 (C-5a), 41.9 (C-5); HRMS: calculated for $[C_{40}H_{38}N_2O_4+Na]^+$ 633.2724, found 633.2748.

(3*R*,4*R*,5*R*,6*S*)-6-(Aminomethyl)-5-(hydroxymethyl)piperidine-3,4-diol or (5a*S*)-5a-*C*-aminomethyl isofagomine (11)

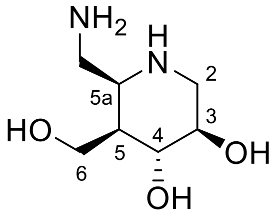

A mixture of **10** (100 mg, 0.16 mmol), 20% palladium hydroxide on carbon (20 mg, 0.0285 mmol), and 100 μL of 37% HCl in H_2O in methanol (2 mL) was stirred under a hydrogen atmosphere for 24 h. The reaction solution was filtered through Celite and the filtrate was concentrated. The residue was purified by CC to give **11** as a yellow oil (23 mg, 0.13 mmol, 81%); TLC (*n*-propanol/NH_4OH = 2/1, v/v) R_f = 0.45; [α]D25 = +22.6° (*c* = 0.1 in H_2O); ^{1}H NMR (600 MHz, D_2O) δ 4.03–4.00 (m, 1H, H-5a), 3.96 (dd, *J* = 7.7, 12.2 Hz, 1H, H-6), 3.90–3.87 (m, 3H, H-3, H-4, and H-6), 3.53 (dd, *J* = 6.7, 13.9 Hz, 1H, *CH2*NH2), 3.49 (dd, *J* = 1.8, 11.4 Hz, 1H, H-2), 3.45 (dd, *J* = 7.1, 13.9 Hz, 1H, *CH2*NH2), 3.18 (dd, *J* = 3.8, 11.4 Hz, 1H, H-2), 2.30–2.27 (m, 1H, H-5); ^{13}C NMR (150 MHz, D_2O) δ 70.7 (C-3), 69.5 (C-4), 61.0 (C-6), 54.3 (C-5a), 47.2 (C-2), 45.3 (*CH2*NH2), 40.0 (C-5); HRMS: calculated for $[C_7H_{16}N_2O_3+H]^+$ 177.1234, found 177.1248.

(3*R*,4*R*,5*R*)-5-(Hydroxymethyl)piperidine-3,4-diol or isofagomine (IFG)

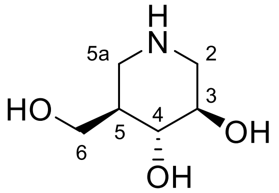

A mixture of **1** (100 mg, 0.17 mmol), 20% palladium hydroxide on carbon (20 mg, 0.0285 mmol), and 100 μL of 37% HCl in H_2O in methanol (2 mL) was stirred under a hydrogen atmosphere for 24 h. The reaction solution was filtered through Celite and the filtrate was concentrated. The residue was purified by CC to give IFG (22 mg, 0.15 mmol, 88%); TLC (*n*-propanol/NH_4OH = 9/1, v/v) R_f = 0.45; [α]D25 = -3.0° (*c* = 0.5 in H_2O) ^{1}H NMR (600 MHz, D_2O) δ 3.82 (dd, *J* = 3.4, 11.5 Hz, 1H, H-6), 3.65 (dd, *J* = 6.7, 11.5 Hz, 1H, H-6), 3.54–3.58 (m, 1H, H-3), 3.27 (pt, *J* = 9.3 Hz, 1H, H-

4), 3.22 (dd, J = 5.3, 12.3 Hz, 1H, H-2), 3.18 (dd, J = 3.4, 13.2 Hz, 1H, H-5a), 2.48–2.55 (m, 2H, H-2 and H-5a), 1.73–1.77 (m, 1H, H-5); ^{13}C NMR (150 MHz, D_2O) δ73.3 (C-3), 71.4 (C-4), 60.3 (C-6), 49.0 (C-2), 46.1 (C-5a), 43.9 (C-5); HRMS: calculated for $[C_6H_{13}NO_3+H]^+$ 148.0968, found 148.0970.

(3*R*,4*R*,5*R*)-3,4-Bis(benzyloxy)-6-cyano-5-((trityloxy)methyl)-2,3,4,5-tetrahydropyridine 1-oxide or (3*R*,4*R*,5*R*)-3,4-bis(benzyloxy)-5a-cyano-oxidoimino-5-(trityloxy)methyl isofagomine (12)

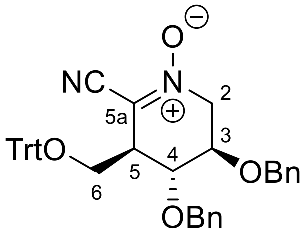

A mixture of **10** (1 g, 1.64 mmol) and MnO_2 (289 mg, 4.92 mmol) in dry CH_2Cl_2 (20 mL) was stirred at room temperature for 2 h. The reaction solution was filtered through Celite and the filtrate was concentrated. The residue was purified by CC to give **12** (0.89 g, 1.46 mmol, 89%); TLC (Hexanes/EtOAc = 3/1, v/v) R_f = 0.35; [α]D25 = -3.66° (c = 3.9 in CH_2Cl_2)^{1}H NMR (600 MHz, $CDCl_3$) δ 7.46–7.40 (m, 6H, Ph-H), 7.36–7.29 (m, 12H, Ph-H), 7.28–7.24 (m, 3H, Ph-H), 7.23–7.19 (m, 2H, Ph-H), 7.15–7.10 (m, 2H, Ph-H), 4.54 (d, J = 11.8 Hz, 1H, PhCH_2), 4.49 (d, J = 11.8 Hz, 1H, PhCH_2), 4.36 (s, 2H, PhCH_2), 4.16–4.11 (m, 1H, H-2), 4.09 (dd, J = 2.2, 4.8 Hz, 1H, H-4), 3.96 (dd, J = 3.2, 15.9 Hz, 1H, H-2), 3.85–3.89 (m, 1H, H-3), 3.65 (dd, J = 4.6, 9.9 Hz, 1H, H-6), 3.56 (dd, J = 8.7 Hz, 9.9 Hz, 1H, H-6), 2.86–2.81 (m, 1H, H-5); ^{13}C NMR (150 MHz, $CDCl_3$) δ 143.5 (Ph-C), 137.2 (Ph-C), 136.6 (Ph-C), 128.9 (Ph-C), 128.8 (Ph-C), 128.7 (Ph-C), 128.5 (Ph-C), 128.4 (Ph-C), 128.2 (Ph-C), 127.9 (Ph-C), 127.87 (Ph-C), 127.6 (Ph-C), 118.3 (C-5a), 113.2 (C≡N), 87.9 (C(Ph)$_3$), 73.0 (C-3), 72.2 (C-4), 71.8 (PhCH_2), 69.8 (PhCH_2), 62.1 (C-6), 61.6 (C-2), 44.2 (C-5); HRMS: calculated for $[C_{40}H_{36}N_2O_4+Na]^+$ 631.2567, found 631.2581.

(3*R*,4*R*,5*R*,6*R*)-6-(Aminomethyl)-5-(hydroxymethyl)piperidine-3,4-diol or (5a*R*)-5a-*C*-aminomethyl isofagomine (14)

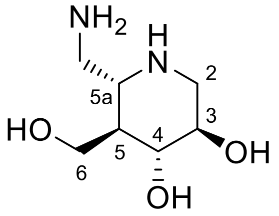

A solution of **12** (1.5 g, 2.46 mmol) in MeOH (25 mL) was added to $NaBH_4$ (140 mg, 3.7 mmol) at 0 ^oC. Then the reaction was warmed to rt and stirred for 1 h. The mixture was quenched with H_2O and extracted with EtOAc and H_2O. The organic layers were dried ($MgSO_4$), filtered, and concentrated. The residue was purified by CC chromatography (*n*-hexane / EtOAc = 3:1, silica gel) to give **13** (1.2 g, 1.97 mmol, 80%). A mixture of **13** (100 mg, 0.16 mmol), 20% palladium hydroxide on carbon (20 mg, 0.0285 mmol), and 100 μL of 37% HCl in H_2O in methanol (2 mL) was stirred under a hydrogen atmosphere for 24 h. The reaction solution was filtered through a Celite and the filtrate was concentrated. The residue was purified by CC to give **14** (23 mg, 0.13 mmol, 81%); TLC (*n*-propanol/NH_4OH = 2/1, v/v) R_f = 0.5; [α]D25 = -13.0^o (*c* = 0.1 in H_2O); ^1H NMR (600 MHz, D_2O) δ 3.84–3.78 (m, 2H, H-6), 3.52–3.47 (m, 1H, H-3), 3.42 (pt, *J* = 10.2 Hz, 1H, H-4), 3.29 (dd, *J* = 3.2, 13.4 Hz, 1H, CH_2NH_2), 3.16 (dd, *J* = 5.0, 12.6 Hz, 1H, H-2), 2.93–2.90 (m, 1H, CH_2NH_2), 2.84–2.80 (m, 1H, H-5a), 2.43 (dd, *J* = 11.2, 12.6 Hz, 1H, H-2), 1.43–1.38 (m, 1H, H-5); ^{13}C NMR (150 MHz, D_2O) δ 72.4 (C-3), 72.1 (C-4), 57.5 (C-6), 54.8 (C-5a), 48.7 (C-2), 47.2 (CH_2NH_2), 41.7 (C-5); HRMS: calculated for $[C_7H_{16}N_2O_3+H]^+$ 177.1234, found 177.1221.

General procedure for resynthesis of IFG derivatives

A mixture of scaffold **11** (or **14)** (0.05–0.06 mmol), EDC (0.15–0.18 mmol), and the desired carboxylic acid (0.065–0.078 mmol) in DMF was stirred at room temperature for 12 h. The mixture was concentrated and the residue was purified by CC (11% aqueous NH_4OH in *n*-propanol, silica gel) to give the corresponding amide product.

***N*-(((2*S*,3*R*,4*R*,5*R*)-4,5-Dihydroxy-3-(hydroxymethyl)piperidin-2-yl)methyl)-2-naphthamide or (5a*S*)-5a-*C*-(2-naphthamido)methyl isofagomine (S1)**

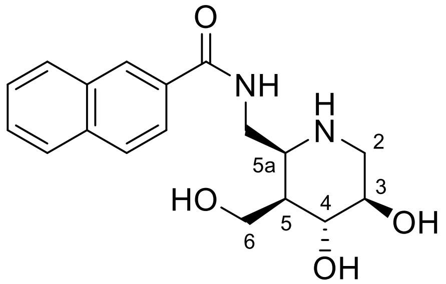

Yield: 90%. [α]D25 = +28.4^o (*c* = 0.1 in H_2O); ^1H NMR (600 MHz, D_2O) δ 8.39 (s, 1H, Ar-H), 8.09–8.03 (m, 3H, Ar-H), 7.80 (dd, *J* = 1.7, 8.6 Hz, 1H, Ar-H), 7.70–7.64 (m, 2H, Ar-H), 4.00 (dd, *J* = 4.9, 11.7 Hz, 1H, H-6), 3.79–3.74 (m, 2H, H-3 and H-6), 3.69–3.63 (m, 2H, H-4 and H-5a), 3.57–3.54 (m, 1H, H-2), 3.51 (dd, *J* = 4.0, 14.0 Hz, 1H, CH_2NHCO), 3.11 (dd, *J* = 4.7, 13.0 Hz, 1H, H-2), 2.93–2.89 (m, 1H, CH_2NHCO), 2.14–2.10 (m, 1H, H-5); ^{13}C NMR (150 MHz, D_2O) δ 171.0

(CONNH), 134.6 (Ar-C), 132.2 (Ar-C), 130.8 (Ar-C), 128.9 (Ar-C), 128.5 (Ar-C), 128.2 (Ar-C), 127.8 (Ar-C), 127.7 (Ar-C), 127.1 (Ar-C), 123.4 (Ar-C), 72.0 (C-3), 71.3 (C-4), 59.9 (C-6), 52.2 (C-5a), 45.7 (C-2), 44.0 (*CH2*NHCO), 36.5 (C-5); HRMS: calculated for $[C_{18}H_{22}N_2O_4+H]^+$ 331.1652, found 331.1673.

(*E*)-*N*-(((2*R*,3*R*,4*R*,5*R*)-4,5-Dihydroxy-3-(hydroxymethyl)piperidin-2-yl)methyl)-2-methyl-3-phenylacrylamide or (5a*R*)-5a-*C*-((*E*)-2-methyl-3-phenylacrylamido)methyl isofagomine (15)

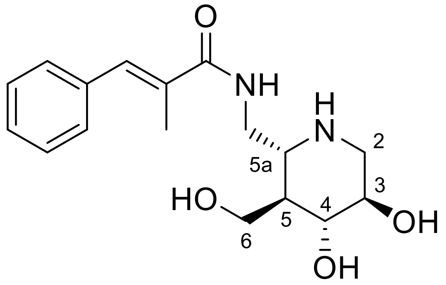

Yield: 23%. [α]D25 = -10.5° (*c* = 0.1 in H_2O); ^{1}H NMR (600 MHz, D_2O) *δ* 7.51–7.47 (m, 4H, Ph-H), 7.43–7.41 (m, 1H, Ph-H), 7.30 (s, 1H, *CH*=C), 3.94 (dd, *J* = 3.0, 11.9 Hz, 1H, H-6), 3.89 (dd, *J* = 2.5, 11.9 Hz, 1H, H-6), 3.68 (dd, *J* = 3.1, 14.4 Hz, 1H, *CH2*NHCO), 3.55–3.47 (m, 2H, H-3 and H-4), 3.40 (dd, *J* = 7.5, 14.4 Hz, 1H, *CH2*NHCO), 3.15 (dd, *J* = 4.7, 12.1 Hz, 1H, H-2), 2.89–2.85 (m, 1H, H-5a), 2.44 (pt, *J* = 11.3 Hz, 1H, H-2), 1.93 (s, 3H, CH_3), 1.42–1.38 (m, 1H, H-5); ^{13}C NMR (150 MHz, D_2O) *δ* 173.4 (CONH), 135.7 (*CH*=C), 134.4 (Ph-C), 131.7 (Ph-C), 129.4 (Ph-C), 128.5 (Ph-C), 128.3 (CH=*C*(CH_3)CONH), 72.7 (C-3), 72.1 (C-4), 57.5 (C-6), 55.1 (C-5a), 49.0 (C-2), 46.9 (*CH2*NHCO), 42.0 (C-5), 13.6 (CH_3); HRMS: calculated for $[C_{17}H_{24}N_2O_4+H]^+$ 321.1809, found 321.1812.

(*E*)-3-(3-Bromophenyl)-*N*-(((2*R*,3*R*,4*R*,5*R*)-4,5-dihydroxy-3-(hydroxymethyl)piperidin-2-yl)methyl)acrylamide or (5a*R*)-5a-*C*-((*E*)-3-(3-bromophenyl)acrylamido)methyl isofagomine (16)

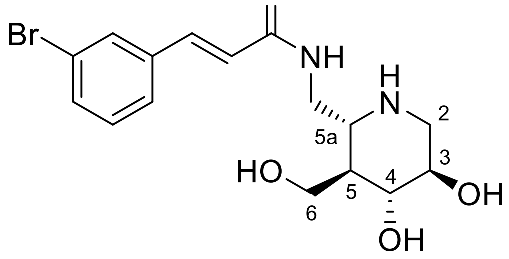

Yield: 17%. [α]D25 = -5.9° (*c* = 0.1 in H_2O); ^{1}H NMR (600 MHz, D_2O) *δ* 7.77 (s, 1H, Ar-H), 7.56

(d, J = 8.0 Hz, 1H, Ar-H), 7.53 (d, J = 7.8 Hz, 1H, Ar-H), 7.41 (d, J = 15.8 Hz, 1H, BrC_6H_5*CH*=CH), 7.32 (pt, J = 7.9 Hz, 1H, Ar-H), 6.57 (d, J = 15.8 Hz, 1H, CH=*CH*CONH), 3.96 (dd, J = 2.8, 12.1 Hz, 1H, H-6), 3.87 (dd, J = 2.3, 12.1 Hz, 1H, H-6), 3.74 (dd, J = 2.9, 14.6 Hz, 1H, $\mathit{CH_2}$NHCO), 3.59–3.50 (m, 2H, H-3 and H-4), 3.41 (dd, J = 7.3, 14.6 Hz, 1H, $\mathit{CH_2}$NHCO), 3.21 (dd, J = 4.9, 12.2 Hz, 1H, H-2), 2.98–2.94 (m, 1H, H-5a), 2.51 (pt, J = 11.4 Hz, 1H, H-2), 1.46 (m, 1H, H-5); ^{13}C NMR (150 MHz, D_2O) δ 168.7 (CONH), 139.9 (BrC_6H_5*CH*=CH), 136.5 (Ar-C), 132.7 (Ar-C), 130.6 (Ar-C), 130.5 (Ar-C), 126.8 (Ar-C), 122.3 (Ar-C), 121.1 (CH=*CH*CONH), 72.2 (C-3), 71.4 (C-4), 57.2 (C-6), 55.4 (C-5a), 48.5 (C-2), 46.1 ($\mathit{CH_2}$NHCO), 41.1 (C-5); HRMS: calculated for $[C_{16}H_{21}BrN_2O_4+H]^+$ 385.0757, found 385.0761.

2-(4-Bromophenyl)-*N*-(((2*R*,3*R*,4*R*,5*R*)-4,5-dihydroxy-3-(hydroxymethyl)piperidin-2-yl)methyl)acetamide or (5a*R*)-5a-*C*-(2-(4-bromophenyl)acetamido)methyl isofagomine (17)

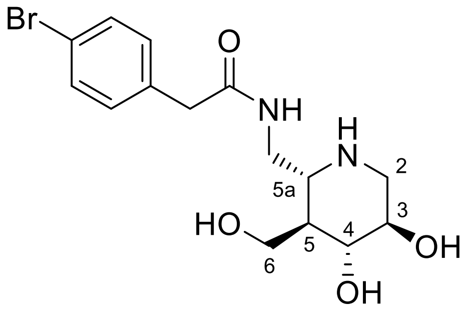

Yield: 65%. [α]D25 = -15.3º (c = 0.1 in H_2O); ^{1}H NMR (600 MHz, D_2O) δ 7.55 (d, J = 8.3 Hz, 2H, Ar-H), 7.21 (d, J = 8.3 Hz, 2H, Ar-H), 3.85 (dd, J = 2.6, 11.9 Hz, 1H, H-6), 3.76 (dd, J = 2.1, 11.9 Hz, 1H, H-6), 3.61–3.54 (m, 3H, CO$\mathit{CH_2}$ and $\mathit{CH_2}$NHCO), 3.49–3.43 (m, 2H, H-3 and H-4), 3.28 (dd, J = 7.2, 14.5 Hz, 1H, $\mathit{CH_2}$NHCO), 3.12 (dd, J = 4.5, 12.1 Hz, 1H, H-2), 2.81–2.78 (m, 1H, H-5a), 2.41 (pt, J = 11.3 Hz, 1H, H-2), 1.33–1.29 (m, 1H, H-5); ^{13}C NMR (150 MHz, D_2O) δ 174.6 (CONH), 134.1 (Ar-C), 131.7 (Ar-C), 131.0 (Ar-C), 120.5 (Ar-C), 72.3 (C-3), 71.7 (C-4), 57.2 (C-6), 55.0 (C-5a), 48.6 (C-2), 46.3 ($\mathit{CH_2}$NHCO) , 41.6 (CO$\mathit{CH_2}$), 41.3 (C-5); HRMS: calculated for $[C_{15}H_{21}BrN_2O_4+H]^+$ 373.0757, found 373.0734.

N-(((2*S*,3*R*,4*R*,5*R*)-4,5-Dihydroxy-3-(hydroxymethyl)piperidin-2-yl)methyl)-4-methyl-2-nitrobenzamide or (5a*S*)-5a-*C*-(4-methyl-2-nitrobenzamido)methyl isofagomine (18)

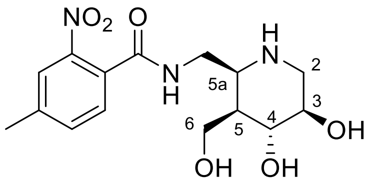

Yield: 35%. [α]D25 = +19.6º (c = 0.1 in H_2O); ^{1}H NMR (600 MHz, MeOD) δ 7.95 (s, 1H, Ar-H), 7.61 (d, J = 7.7 Hz, 1H, Ar-H), 7.54 (d, J = 7.7 Hz, 1H, Ar-H), 4.00 (dd, J = 4.9, 11.4 Hz, 1H, H-6), 3.76–3.69 (m, 2H, H-6 and CH_2NHCO), 3.55 (dd, J = 7.9, 8.6 Hz, 1H, H-4), 3.50–3.47 (m, 2H, H-3 and CH_2NHCO), 3.40 (m, 1H, H-5a), 2.99 (dd, J = 4.5, 12.6 Hz, 1H, H-2), 2.85 (dd, J = 8.9, 12.6 Hz, 1H, H-2), 2.50 (s, 3H, CH_3), 2.03–2.01 (m, 1H, H-5); ^{13}C NMR (150 MHz, MeOD) δ 168.9 (CONH), 146.7 (Ar-C), 142.0 (Ar-C), 134.0 (Ar-C), 129.4 (Ar-C), 128.5 (Ar-C), 124.4 (Ar-C), 70.4 (C-4), 70.3 (C-3), 59.7 (C-6), 53.3 (C-5a), 45.4 (C-2), 44.1 (C-5), 37.9 (CH_2NHCO), 19.6 (CH_3); HRMS: calculated for $[C_{15}H_{21}N_3O_6+H]^+$ 340.1503, found 340.1504.

(*E*)-3-(5-Bromo-2-methoxyphenyl)-*N*-(((2*S*,3*R*,4*R*,5*R*)-4,5-dihydroxy-3-(hydroxymethyl)piperidin-2-yl)methyl)acrylamide or (5a*S*)-5a-*C*-((*E*)-3-(5-bromo-2-methoxyphenyl)acrylamido)methyl isofagomine (19)

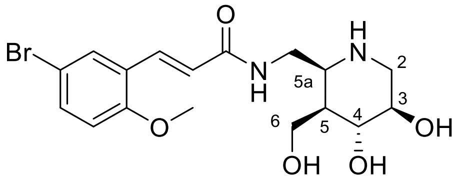

Yield: 60%. [α]D25 = +12.1º (c = 0.1 in H_2O); ^{1}H NMR (600 MHz, MeOD) δ 7.81 (d, J = 15.9 Hz, 1H, $BrC_6H_5OCH_3$*CH*=CH), 7.70 (s, 1H, Ar-H), 7.49 (d, J = 8.9 Hz, 1H, Ar-H), 7.00 (d, J = 8.9 Hz, 1H, Ar-H), 6.74 (d, J = 15.9 Hz, 1H, CH=*CH*CONH), 3.98 (dd, J = 4.6, 11.9 Hz, 1H, H-6), 3.91 (s, 3H, CH_3), 3.91–3.88 (m, 1H, H-6), 3.86–3.84 (m, 2H, H-4 and CH_2NHCO), 3.78–3.75 (m, 2H, H-3 and H-5a), 3.59 (dd, J = 4.7, 14.4 Hz, 1H, CH_2NHCO), 3.36–3.33 (m, 1H, H-2), 3.18 (dd, J = 5.0, 13.0 Hz, 1H, H-2), 2.16–2.14 (m, 1H, H-5); ^{13}C NMR (150 MHz, MeOD) δ 168.3 (CONH), 157.4 (Ar-C), 134.9 ($BrC_6H_5OCH_3$*CH*=CH), 133.4 (Ar-C), 130.4 (Ar-C), 125.5 (Ar-C), 121.7 (CH=*CH*CONH), 113.1 (Ar-C), 112.5 (Ar-C), 69.1 (C-4), 67.0 (C-3), 59.1 (C-6), 55.0 (CH_3), 54.0 (C-5a), 45.3 (C-2), 43.4 (C-5), 38.1 (CH_2NHCO); HRMS: calculated for $[C_{17}H_{23}BrN_2O_5+H]^+$ 415.0863, found 415.0865.

N-(((2*S*,3*R*,4*R*,5*R*)-4,5-Dihydroxy-3-(hydroxymethyl)piperidin-2-yl)methyl)-2,4-dimethoxybenzamide or (5a*S*)-5a-*C*-(2,4-dimethoxybenzamido)methyl isofagomine (20)

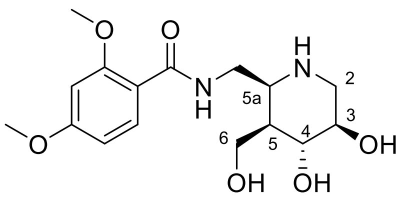

Yield: 61%. [α]D25 = +25.6º (*c* = 0.1 in H_2O); ^{1}H NMR (600 MHz, MeOD) *δ* 8.00 (d, *J* = 9.3 Hz, 1H, Ar-H), 6.66–6.65 (m, 2H, Ar-H), 4.05–4.00 (m, 2H, H-6 and *CH2*NHCO), 3.99 (s, 3H, CH_3), 3.92–3.85 (m, 5H, H-4, H-6, and CH_3), 3.85–3.80 (m, 2H, H-3 and H-5a), 3.63 (dd, *J* = 6.7, 13.9 Hz, 1H, *CH2*NHCO), 3.39–3.37 (m, 1H, H-2), 3.20 (dd, *J* = 4.2, 12.9 Hz, 1H, H-2), 2.21–2.19 (m, 1H, H-5); ^{13}C NMR (150 MHz, MeOD) *δ* 167.2 (CONH), 164.4 (Ar-C), 159.7 (Ar-C), 132.9 (Ar-C), 113.0 (Ar-C), 105.5 (Ar-C), 97.9 (Ar-C), 68.8 (C-4), 66.4 (C-3), 59.0 (C-6), 55.2 (CH_3), 54.7 (CH_3), 53.5 (C-5a), 45.4 (C-2), 43.4 (C-5), 38.2 (*CH2*NHCO); HRMS: calculated for $[C_{16}H_{24}N_2O_6+H]^+$ 341.1707, found 341.1708.

***N*-(((2*S*,3*R*,4*R*,5*R*)-4,5-Dihydroxy-3-(hydroxymethyl)piperidin-2-yl)methyl)-9-oxo-9*H*-fluorene-1-carboxamide or (5a*S*)-5a-*C*-(9-oxo-9*H*-fluorene-1-carboxamido)methyl isofagomine (21)**

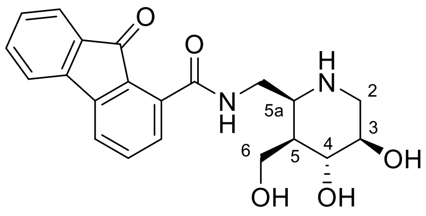

Yield: 46%. [α]D25 = +21.2º (*c* = 0.1 in H_2O); ^{1}H NMR (600 MHz, MeOD) *δ* 7.81 (d, *J* = 7.3 Hz, 1H, Ar-H), *δ* 7.73 (d, *J* = 7.4 Hz, 1H, Ar-H), 7.67–7.61 (m, 2H, Ar-H), *δ* 7.62 (dd, *J* = 7.6, 7.7 Hz, 1H, Ar-H), *δ* 7.55 (d, *J* = 7.7 Hz, 1H, Ar-H), *δ* 7.40 (d, *J* = 7.3, 7.4 Hz, 1H, Ar-H), 4.02 (dd, *J* = 4.9, 11.4 Hz, 1H, H-6), 3.95 (dd, *J* = 11.1, 13.4 Hz, 1H, *CH2*NHCO), 3.75 (dd, *J* = 8.3, 11.4 Hz, 1H, H-6), 3.62–3.56 (m, 3H, H-3, H-4, and H-5a), 3.50 (dd, *J* = 3.7, 13.4 Hz, 1H, *CH2*NHCO), 3.14 (dd, *J* = 3.7, 11.8 Hz, 1H, H-2), 3.02 (dd, *J* = 8.2, 11.8 Hz, 1H, H-2), 2.13–2.11 (m, 1H, H-5); ^{13}C NMR (150 MHz, MeOD) *δ* 193.9 (Ar-CO-Ar), 168.0 (CONH), 145.1 (Ar-C), 143.6 (Ar-C), 135.5 (Ar-C), 135.1 (Ar-C), 134.5 (Ar-C), 133.3 (Ar-C), 129.6 (Ar-C), 129.5 (Ar-C), 128.7 (Ar-C), 124.1 (Ar-C), 122.1 (Ar-C), 120.5 (Ar-C), 71.6 (C-4), 71.2 (C-3), 60.2 (C-6), 53.1 (C-5a), 45.2 (C-2), 45.1 (C-5), 36.7 (*CH2*NHCO); HRMS: calculated for $[C_{21}H_{22}N_2O_5+H]^+$ 383.1601, found 383.1602.

2-(4-Bromophenyl)-*N*-(((2*S*,3*R*,4*R*,5*R*)-4,5-dihydroxy-3-(hydroxymethyl)piperidin-2-yl)methyl)acetamide or (5a*S*)-5a-*C*-(2-(4-

bromophenyl)acetamido)methyl isofagomine (22)

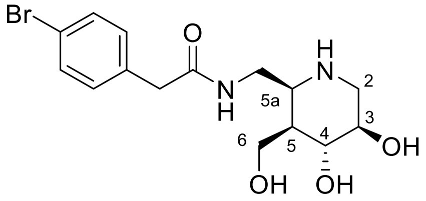

Yield: 14%. [α]D25 = +23.5° (*c* = 0.1 in H_2O); ^{1}H NMR (600 MHz, MeOD) *δ* 7.47 (d, *J* = 8.3 Hz, 2H, Ar-H), 7.24 (d, *J* = 8.3 Hz, 2H, Ar-H), 3.91 (dd, *J* = 4.9, 11.4 Hz, 1H, H-6), 3.65 (dd, *J* = 7.9, 11.4 Hz, 1H, H-6), 3.53–3.49 (m, 3H, H-4 and COC*H*$_2$), 3.47–3.44 (m, 2H, H-3 and *CH*$_2$NHCO), 3.25–3.21 (m, 1H, H-5a), 2.93 (dd, *J* = 4.4, 13.0 Hz, 1H, H-2), 2.72 (dd, *J* = 8.2, 13.0 Hz, 1H, H-2), 1.92–1.90 (m, 1H, H-5); ^{13}C NMR (150 MHz, MeOD) *δ* 172.4 (CONH), 134.9 (Ar-C), 131.2 (Ar-C), 130.8 (Ar-C), 120.3 (Ar-C), 71.9 (C-4), 71.3 (C-3), 60.3 (C-6), 52.7 (C-5a), 45.6 (C-2), 45.2 (C-5), 41.8 (COC*H*$_2$), 37.0 (*CH*$_2$NHCO); HRMS: calculated for $[C_{15}H_{21}BrN_2O_4+H]^+$ 373.0757, found 373.0759.

_N_-(((2*R*,3*R*,4*R*,5*R*)-4,5-Dihydroxy-3-(hydroxymethyl)piperidin-2-yl)methyl)-2-naphthamide or (5a*R*)-5a-*C*-(2-naphthamido)methyl isofagomine (23)

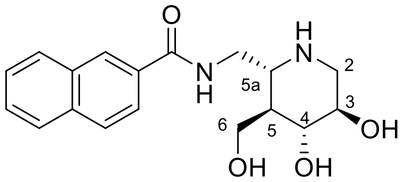

Yield: 64%. [α]D25 = -9.1° (*c* = 0.1 in H_2O); ^{1}H NMR (600 MHz, MeOD) *δ* 8.50 (s, 1H, Ar-H), 8.01 (d, *J* = 7.8 Hz, 1H, Ar-H), 7.98–7.97 (m, 2H, Ar-H), 7.95 (d, *J* = 7.8 Hz, 1H, Ar-H), 7.64–7.58 (m, 2H, Ar-H), 4.05 (dd, *J* = 3.1, 11.5 Hz, 1H, H-6), 3.98–3.95 (m, 2H, H-6 and *CH*$_2$NHCO), 3.77 (dd, *J* = 6.6, 14.9 Hz, 1H, *CH*$_2$NHCO), 3.69–3.65 (m, 1H, H-3), 3.56 (pt, *J* = 10.1 Hz, 1H, H-4), 3.40–3.33 (m, 2H, H-2 and H-5a), 2.74 (dd, *J* = 11.5, 12.1 Hz, 1H, H-2), 1.69–1.66 (m, 1H, H-5); ^{13}C NMR (150 MHz, MeOD) *δ* 170.0 (CONH), 135.0 (Ar-C), 132.6 (Ar-C), 130.7 (Ar-C), 128.7 (Ar-C), 128.0 (Ar-C), 127.8 (Ar-C), 127.7 (Ar-C), 127.4 (Ar-C), 126.6 (Ar-C), 123.6 (Ar-C), 71.3 (C-3), 69.6 (C-4), 57.4 (C-6), 56.8 (C-5a), 47.5 (C-2), 44.5 (C-5), 40.1 (*CH*$_2$NHCO); HRMS: calculated for $[C_{18}H_{22}N_2O_4+H]^+$ 331.1652, found 331.1653.

_N_-(((2*R*,3*R*,4*R*,5*R*)-4,5-Dihydroxy-3-(hydroxymethyl)piperidin-2-yl)methyl)-4-methyl-2-nitrobenzamide or (5a*R*)-5a-*C*-(4-methyl-2-nitrobenzamido)methyl isofagomine (24)

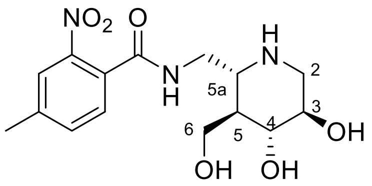

Yield: 51%. [α]D25 = -6.4º (*c* = 0.1 in H_2O); ^{1}H NMR (600 MHz, MeOD) *δ* 7.95 (s, 1H, Ar-H), 7.61 (d, *J* = 7.7 Hz, 1H, Ar-H), 7.54 (d, *J* = 7.7 Hz, 1H, Ar-H), 3.95 (dd, *J* = 3.4, 11.6 Hz, 1H, H-6), 3.86 (dd, *J* = 3.0, 11.6 Hz, 1H, H-6), 3.73 (dd, *J* = 2.8, 14.0 Hz, 1H, CH_2NHCO), 3.55 (dd, *J* = 6.9, 14.0 Hz, 1H, CH_2NHCO), 3.48–3.42 (m, 2H, H-3 and H-4), 3.16 (dd, *J* = 4.5, 11.9 Hz, 1H, H-2), 2.94–2.91 (m, 1H, H-5a), 2.52–2.48 (m, 4H, H-2 and CH_3), 1.46–1.41 (m, 1H, H-5); ^{13}C NMR (150 MHz, MeOD) *δ* 168.7 (CONH), 146.6 (Ar-C), 141.8 (Ar-C), 134.0 (Ar-C), 129.8 (Ar-C), 128.5 (Ar-C), 124.4 (Ar-C), 73.2 (C-3), 72.3 (C-4), 57.8 (C-6), 56.1 (C-5a), 49.7 (C-2), 46.8 (C-5), 41.7 (CH_2NHCO), 19.6 (CH_3); HRMS: calculated for $[C_{15}H_{21}N_3O_6+H]^+$ 340.1537, found 340.1504.

(*E*)-3-(5-Bromo-2-methoxyphenyl)-*N*-(((2*R*,3*R*,4*R*,5*R*)-4,5-dihydroxy-3-(hydroxymethyl)piperidin-2-yl)methyl)acrylamide or (5a*R*)-5a-*C*-((*E*)-3-(5-bromo-2-methoxyphenyl)acrylamido)methyl isofagomine (25)

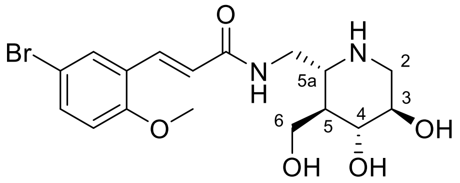

Yield: 68%. [α]D25 = -15.1º (*c* = 0.1 in H_2O); ^{1}H NMR (600 MHz, MeOD) *δ* 7.81 (d, *J* = 15.9 Hz, 1H, $BrC_6H_5OCH_3$*CH*=CH), 7.70 (s, 1H, Ar-H), 7.49 (d, *J* = 6.5 Hz, 1H, Ar-H), 7.00 (d, *J* = 6.5 Hz, 1H, Ar-H), 6.75 (d, *J* = 15.9 Hz, 1H, CH=*CH*CONH), 4.02 (dd, *J* = 2.9, 11.6 Hz, 1H, H-6), 3.91 (s, 3H, CH_3), 3.89–3.85 (m, 2H, H-6 and CH_2NHCO), 3.67–3.62 (m, 1H, H-3), 3.59 (dd, *J* = 6.2, 15.0 Hz, 1H, CH_2NHCO), 3.54 (pt, *J* = 10.1 Hz, 1H, H-4), 3.37–3.29 (m, 2H, H-2 and H-5a), 2.73 (pt, *J* = 11.8 Hz, 1H, H-2),, 1.62–1.58 (m, 1H, H-5); ^{13}C NMR (150 MHz, MeOD) *δ* 168.7 (CONH), 157.4 ($BrC_6H_5OCH_3$*CH*=CH) 135.0 (Ar-C), 133.4 (Ar-C), 130.4 (Ar-C), 125.5 (Ar-C), 121.6 (Ar-C), 113.1 (Ar-C), 112.5 (CH=*CH*CONH), 71.1 (C-3), 69.4 (C-4), 57.2 (C-6), 56.5 (C-5a), 55.0 (CH_3), 47.2 (C-2), 44.1 (C-5), 39.3 (CH_2NHCO); HRMS: calculated for $[C_{17}H_{23}BrN_2O_5+H]^+$ 415.0863, found 415.0862.

N-(((2*R*,3*R*,4*R*,5*R*)-4,5-Dihydroxy-3-(hydroxymethyl)piperidin-2-yl)methyl)-2,4-dimethoxybenzamide or (5a*R*)-5a-*C*-(2,4-dimethoxybenzamido)methyl isofagomine (26)

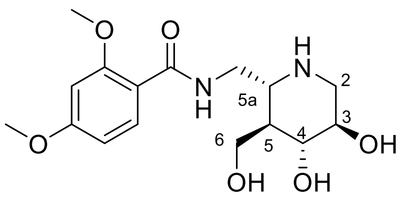

Yield: 72%. [α]D25 = -8.4º (c = 0.1 in H_2O); ^{1}H NMR (600 MHz, MeOD) δ 8.02 (d, J = 9.4 Hz, 1H, Ar-H), 6.67–6.65 (m, 2H, Ar-H), 4.01–3.99 (m, 5H, H-6, *CH_2*NHCO and CH_3), 3.90–3.88 (m, 4H, H-6 and CH_3), 3.68 (dd, J = 5.8, 14.9 Hz, 1H, *CH_2*NHCO), 3.63–3.59 (m, 1H, H-3), 3.44 (pt, J = 10.2 Hz, 1H, H-4), 3.33–3.31 (m, 1H, H-2), 3.31–3.25 (m, 1H, H-5a), 2.73 (pt, J = 11.6 Hz, 1H, H-2),, 1.68–1.64 (m, 1H, H-5); ^{13}C NMR (150 MHz, MeOD) δ 167.6 (CONH), 164.3 (Ar-C), 159.6 (Ar-C), 132.9 (Ar-C), 113.1 (Ar-C), 105.5 (Ar-C), 97.9 (Ar-C), 71.5 (C-3), 69.5 (C-4), 58.2 (C-6), 57.5 (C-5a), 55.2 (CH_3), 54.7 (CH_3), 47.5 (C-2), 44.8 (C-5), 39.8 (*CH_2*NHCO); HRMS: calculated for $[C_{16}H_{24}N_2O_6+H]^+$ 341.1707, found 341.1709.

_N_-(((2_R_,3_R_,4_R_,5_R_)-4,5-Dihydroxy-3-(hydroxymethyl)piperidin-2-yl)methyl)-9-oxo-9_H_-fluorene-1-carboxamide or (5a_R_)-5a-_C_-(9-oxo-9_H_-fluorene-1-carboxamido)methyl isofagomine (27)

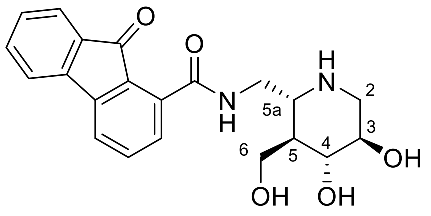

Yield: 64%. [α]D25 = -5.2º (c = 0.1 in H_2O); ^{1}H NMR (600 MHz, MeOD) δ 7.74 (d, J = 6.8 Hz, 1H, Ar-H), 7.68 (d, J = 7.5 Hz, 1H, Ar-H), 7.64–7.61 (m, 3H, Ar-H), 7.56 (d, J = 7.5 Hz, 1H, Ar-H), 7.39 (pt, J = 7.4 Hz, 1H, Ar-H), 4.11 (dd, J = 2.6, 14.9 Hz, 1H, H-6), 4.06 (dd, J = 2.9, 11.7 Hz, 1H, *CH_2*NHCO), 3.89 (dd, J = 2.7, 11.7 Hz, 1H, *CH_2*NHCO), 3.87–3.83 (m, 1H, H-3), 3.72 (dd, J = 6.0, 14.9 Hz, 1H, H-6), 3.61 (pt, J = 10.0 Hz, 1H, H-4), 3.55 (dd, J = 5.0, 12.3 Hz, 1H, H-2), 3.50–3.47 (m, 1H, H-5a), 2.90 (pt, J = 11.9 Hz, 1H, H-2),, 1.78–1.75 (m, 1H, H-5); ^{13}C NMR (150 MHz, MeOD) δ 194.6 (Ar-CO-Ar), 168.5 (CONH), 145.1 (Ar-H), 143.6 (Ar-H), 135.9 (Ar-C), 135.4 (Ar-C), 134.0 (Ar-C),133.1 (Ar-C), 129.6 (Ar-C), 129.5 (Ar-C), 128.6 (Ar-C), 124.4 (Ar-C), 122.4 (Ar-C), 120.7 (Ar-C), 71.1 (C-3), 69.3 (C-4), 57.6 (C-6), 56.5 (C-5a), 47.4 (C-2), 43.7 (C-5), 38.9 (*CH_2*NHCO); HRMS: calculated for $[C_{21}H_{22}N_2O_5+H]^+$ 383.1601, found 383.1602.
