# Supplementary material for: A practical synthesis of nitrone-derived C5a-functionalized isofagomines as protein stabilizers to treat Gaucher disease
Source: Commun Chem. 2024 Apr 20;7:91. doi: 10.1038/s42004-024-01164-9 (PMC11032326; doi:10.1038/s42004-024-01164-9)
Supplement: Supplementary file 2 — Description of Additional Supplementary Files [file 42004_2024_1164_MOESM2_ESM.pdf]

Description of Additional Supplementary Files

File name: Supplementary Data 1

Description: The 1H and 13C NMR spectra of synthesized compounds.
