# Supplementary material for: A practical synthesis of nitrone-derived C5a-functionalized isofagomines as protein stabilizers to treat Gaucher disease
Source: Commun Chem. 2024 Apr 20;7:91. doi: 10.1038/s42004-024-01164-9 (PMC11032326; doi:10.1038/s42004-024-01164-9)
Supplement: Supplementary file 3 — Supplementary Data 1 [file 42004_2024_1164_MOESM3_ESM.pdf]

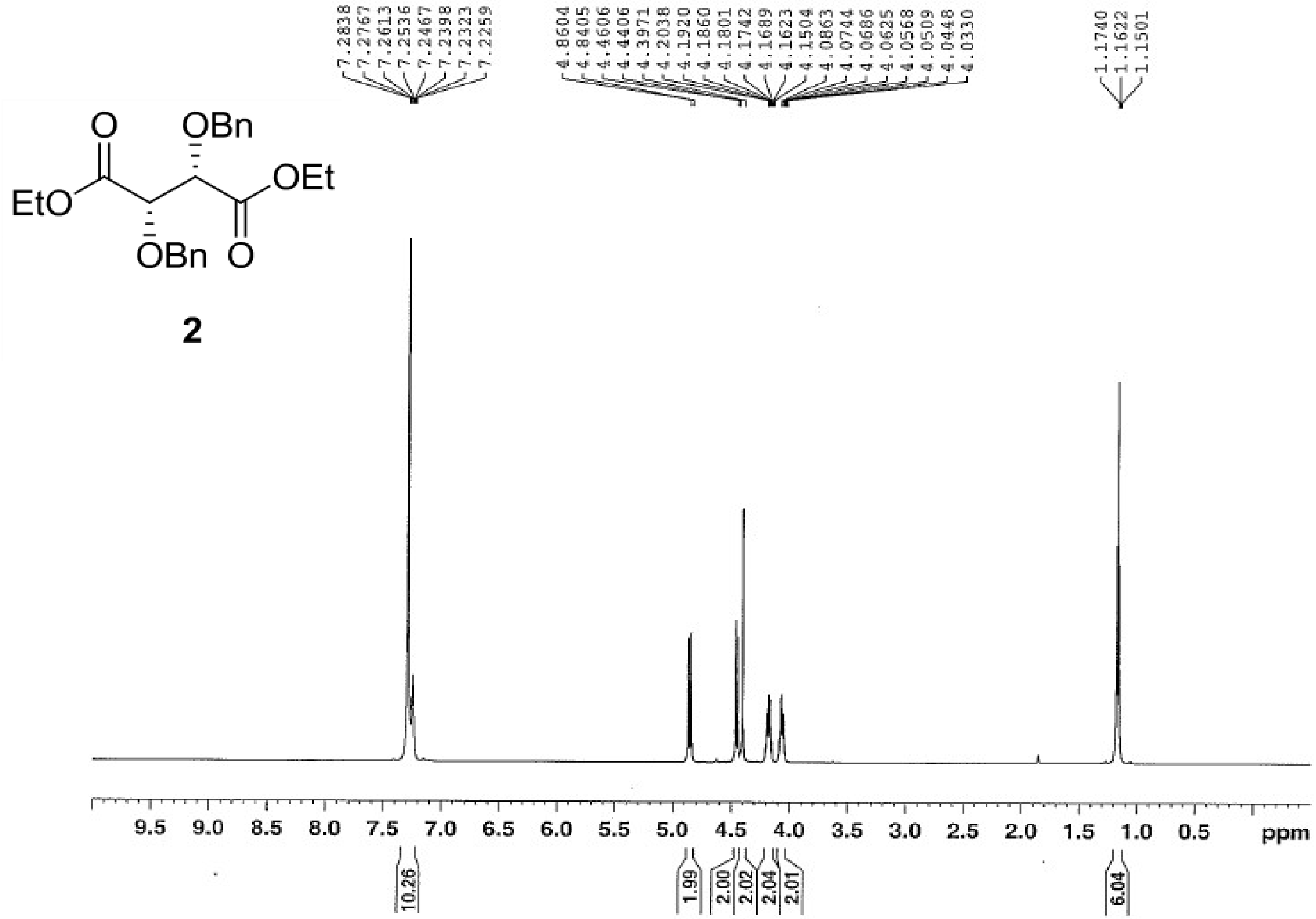

^{1}H spectrum of compound **2 (**600 MHz, $CDCl_3$)

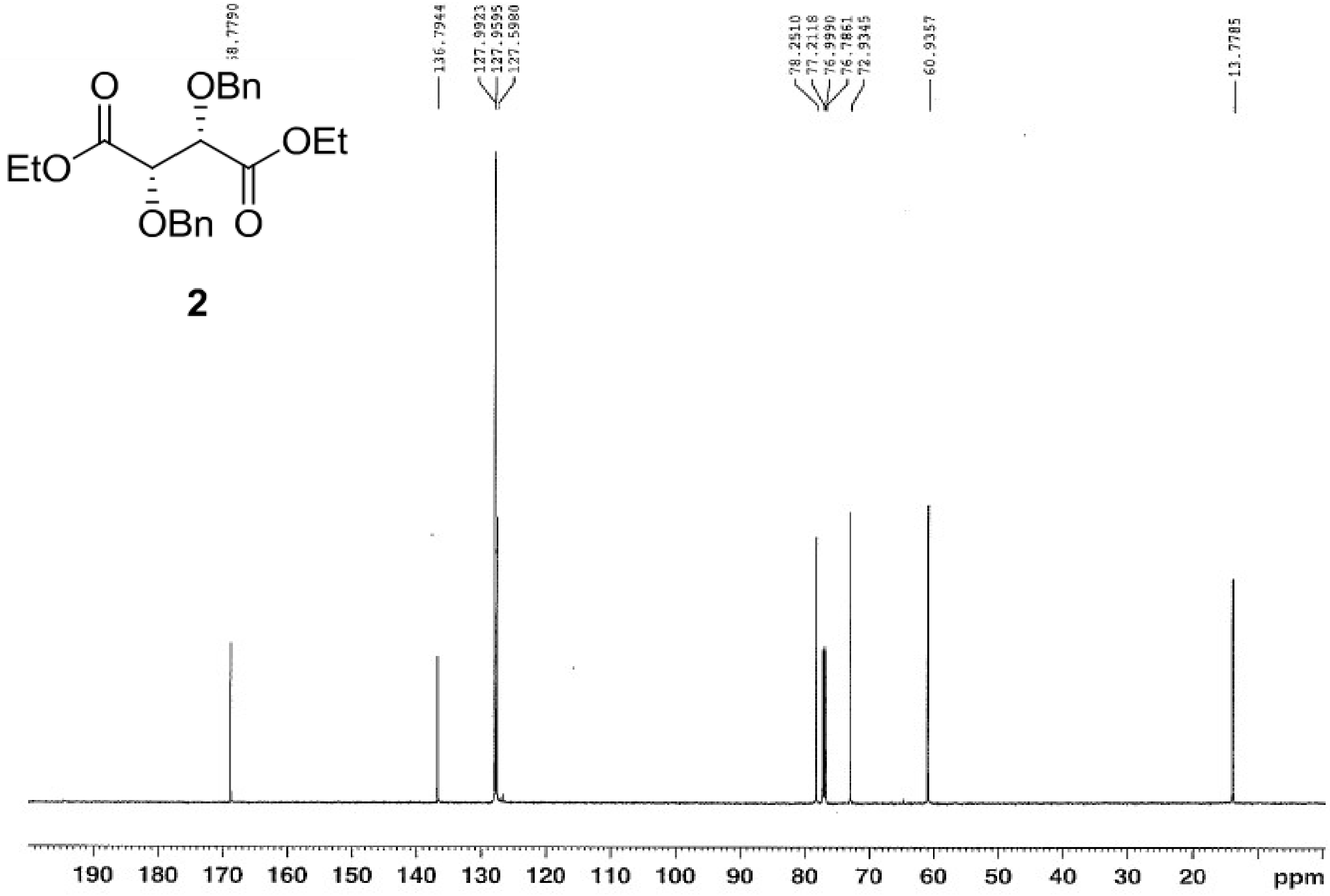

^{13}C spectrum of compound **2** (150 MHz, $CDCl_3$)

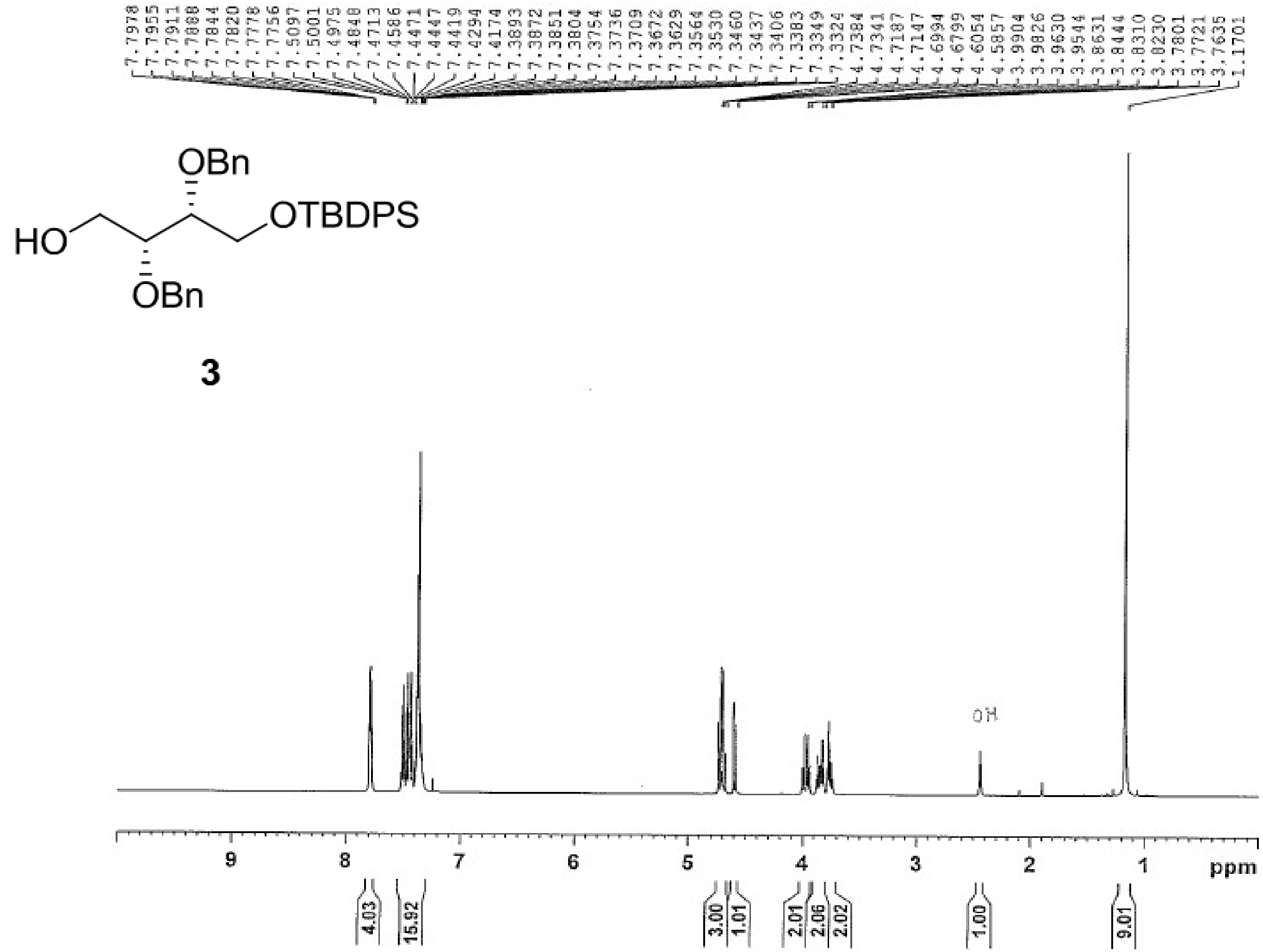

^{1}H spectrum of compound **3 (**600 MHz, $CDCl_3$)

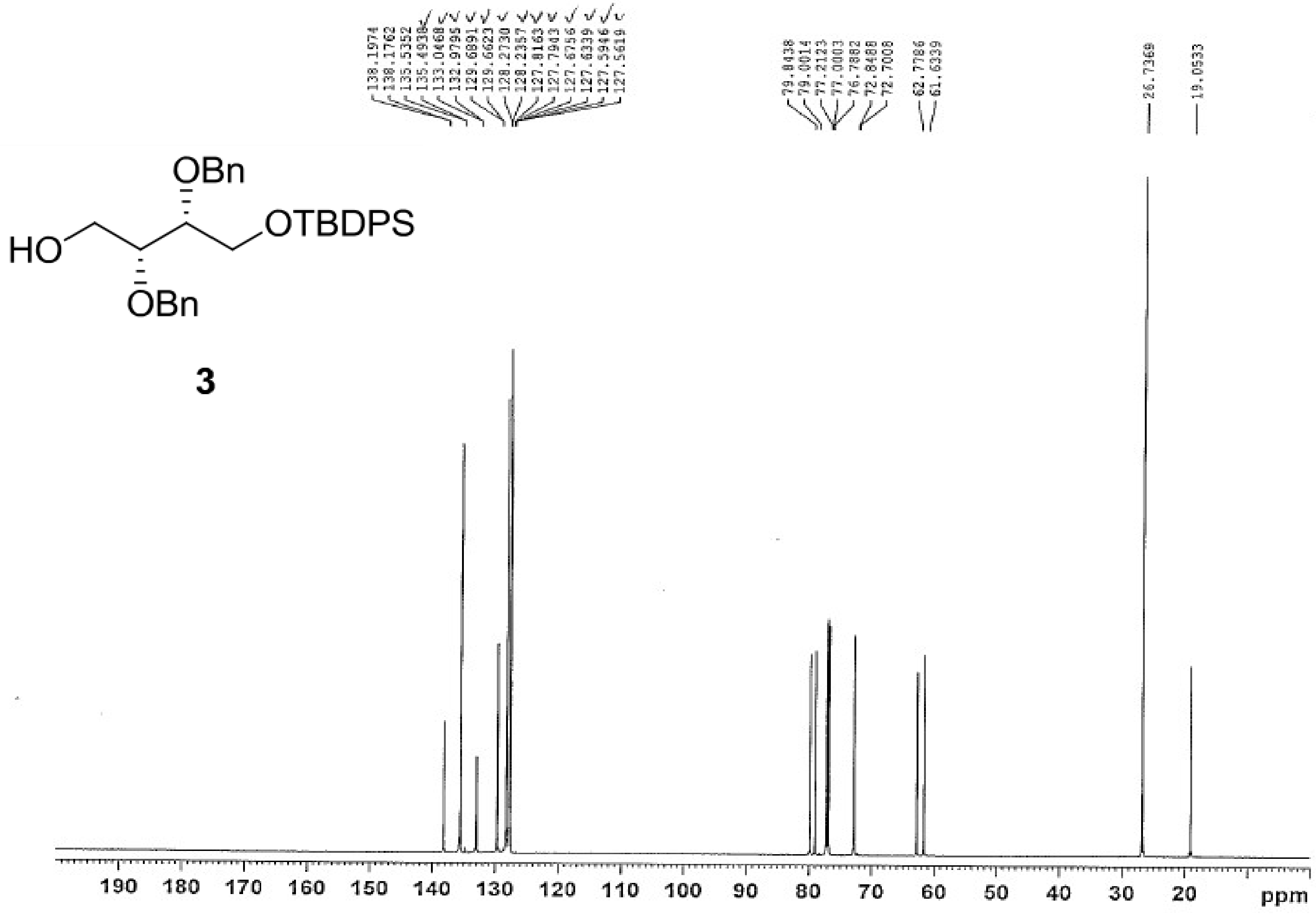

^{13}C spectrum of compound **3 (**150 MHz, $CDCl_3$)

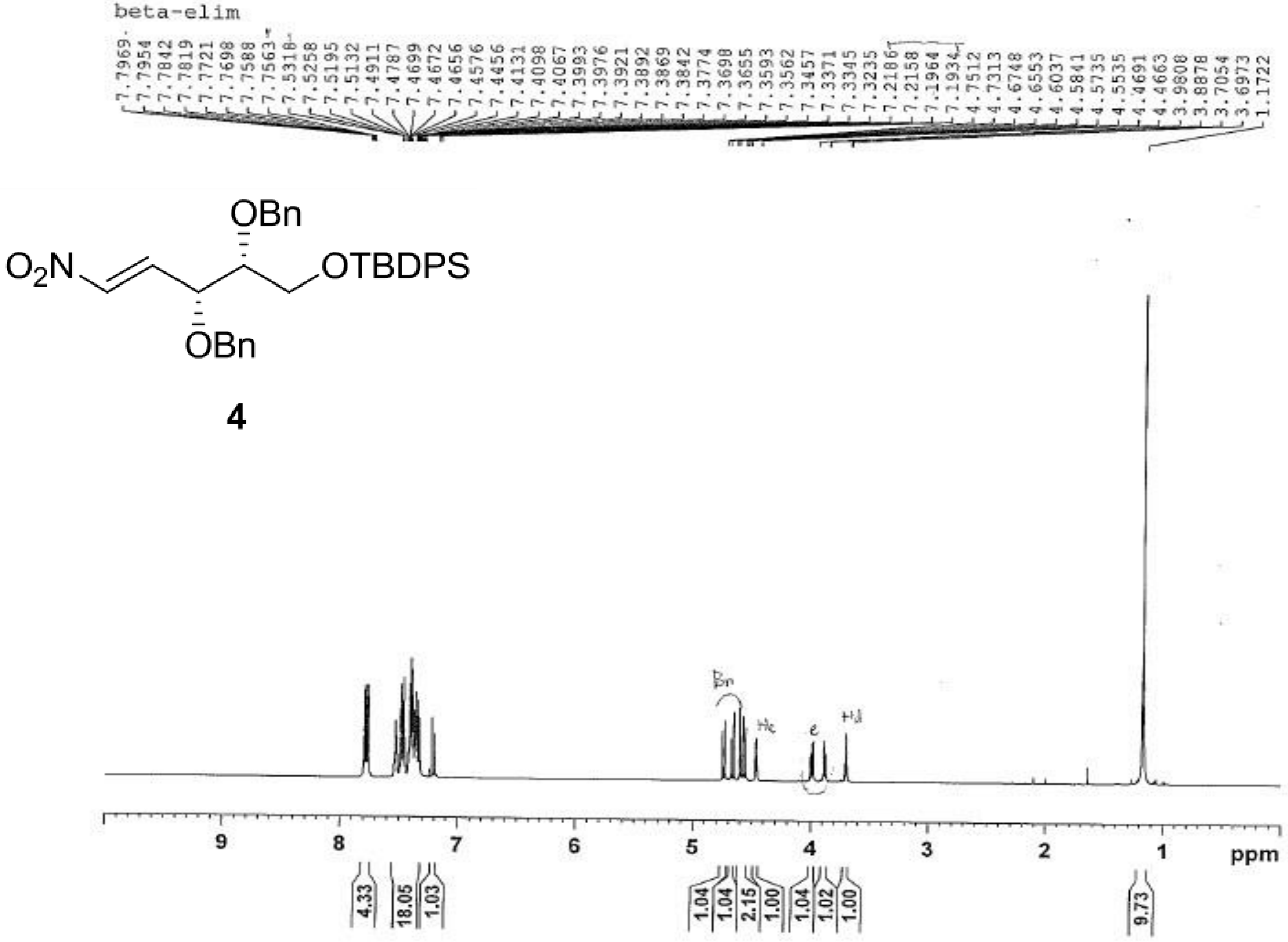

4

^{1}H spectrum of compound **4 (**600 MHz, $CDCl_3$)

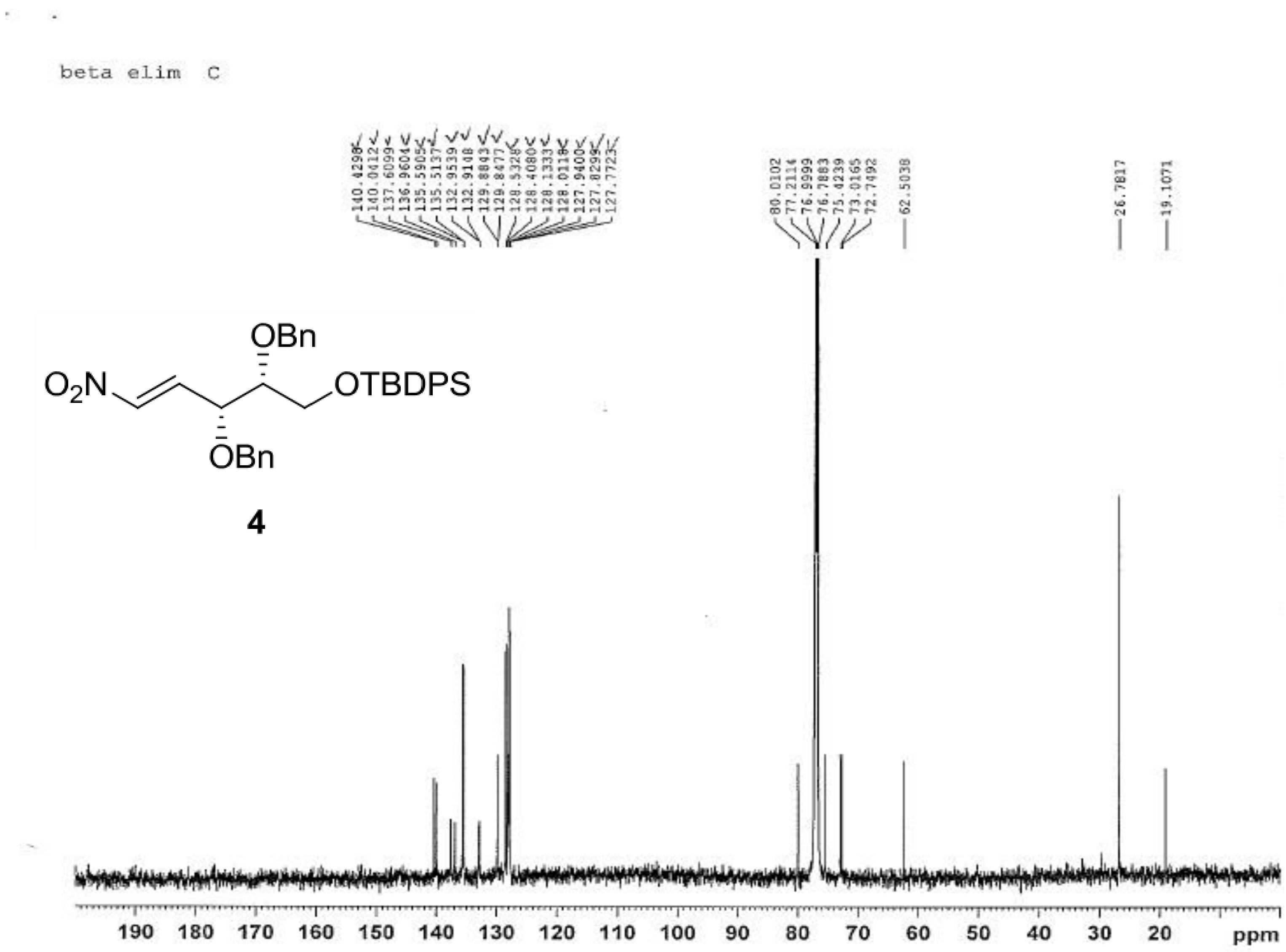

^{13}C spectrum of compound **4** (150 MHz, $CDCl_3$)

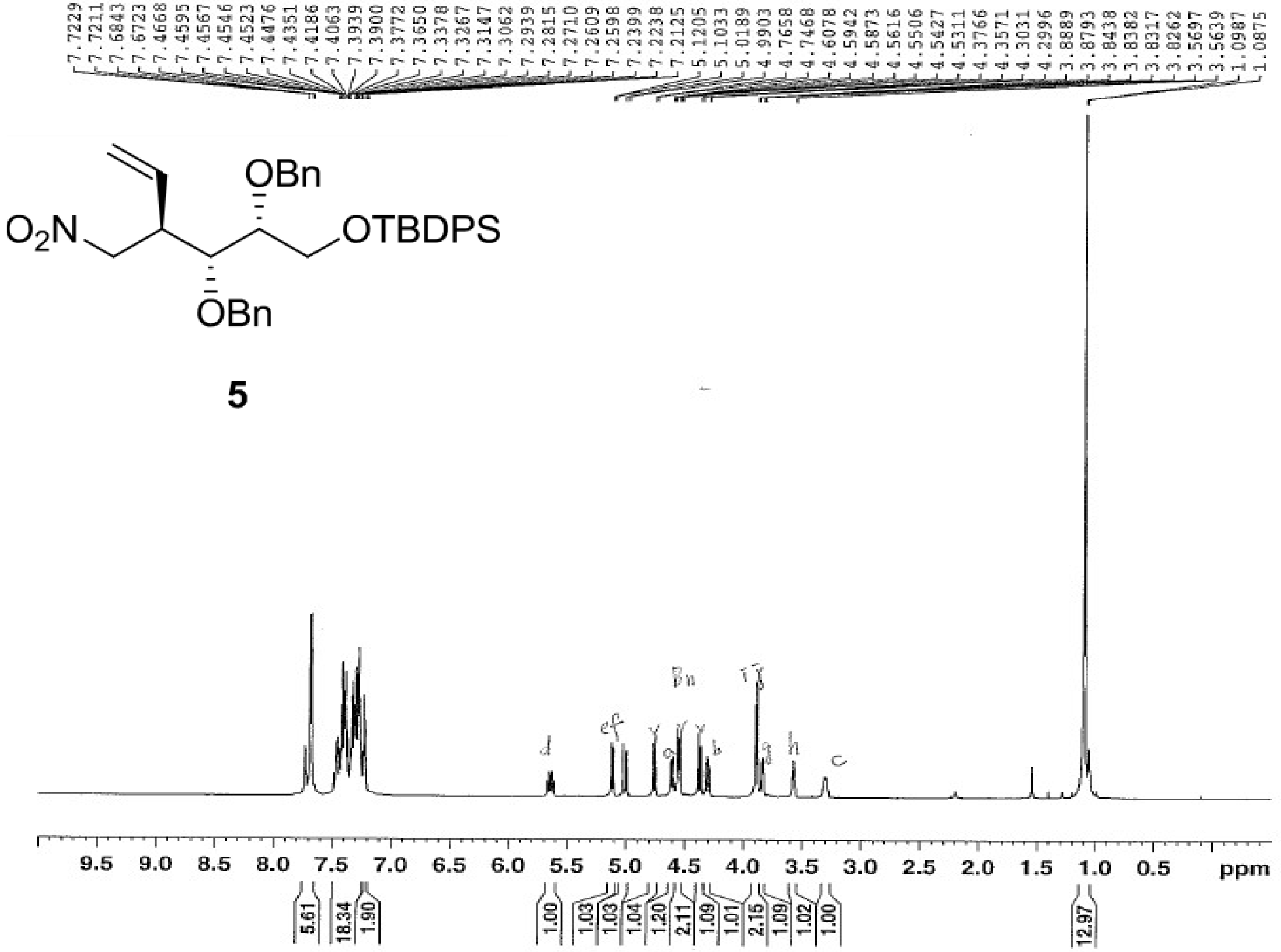

^{1}H spectrum of compound **5 (**600 MHz, $CDCl_3$)

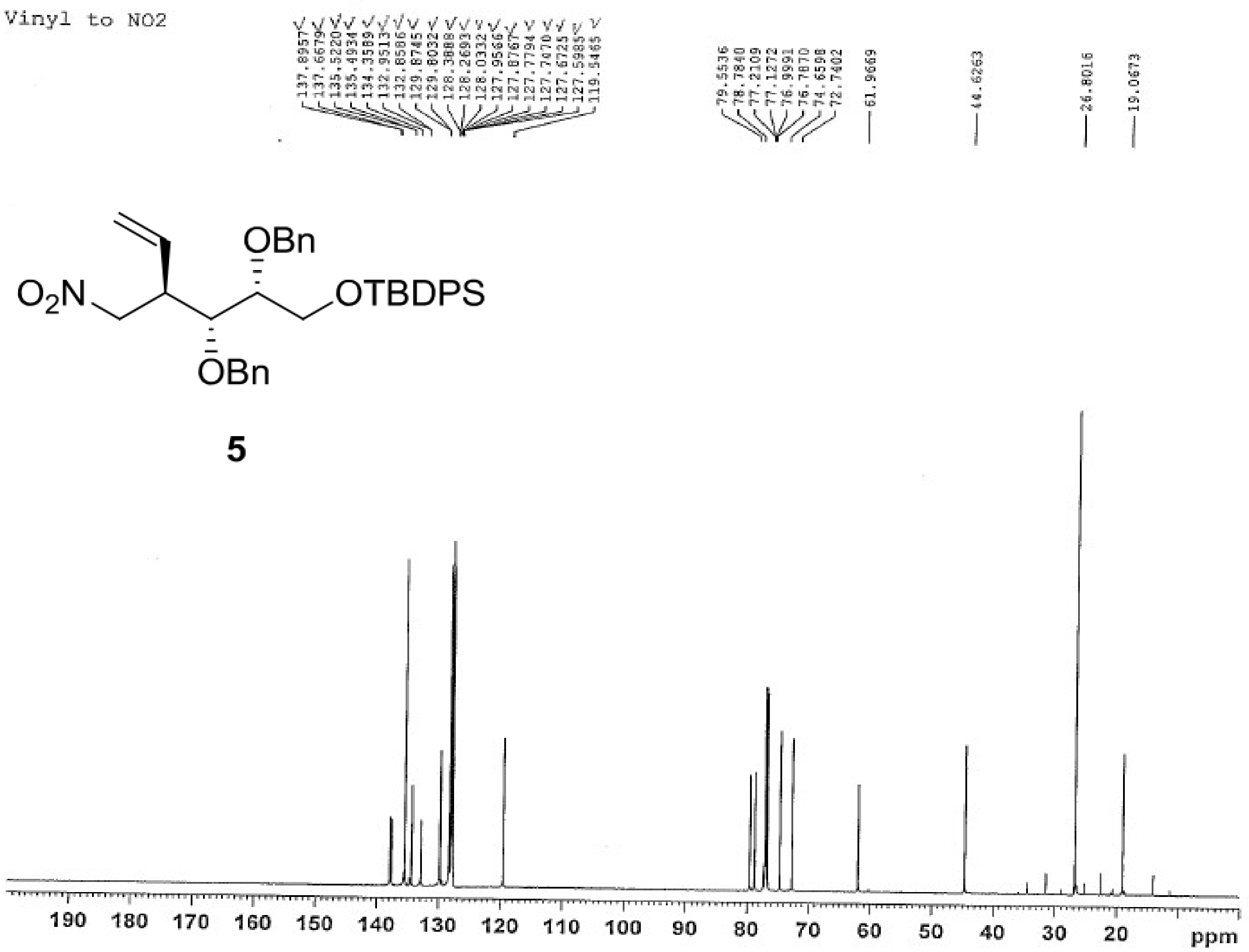

^{13}C spectrum of compound **5** (150 MHz, $CDCl_3$)

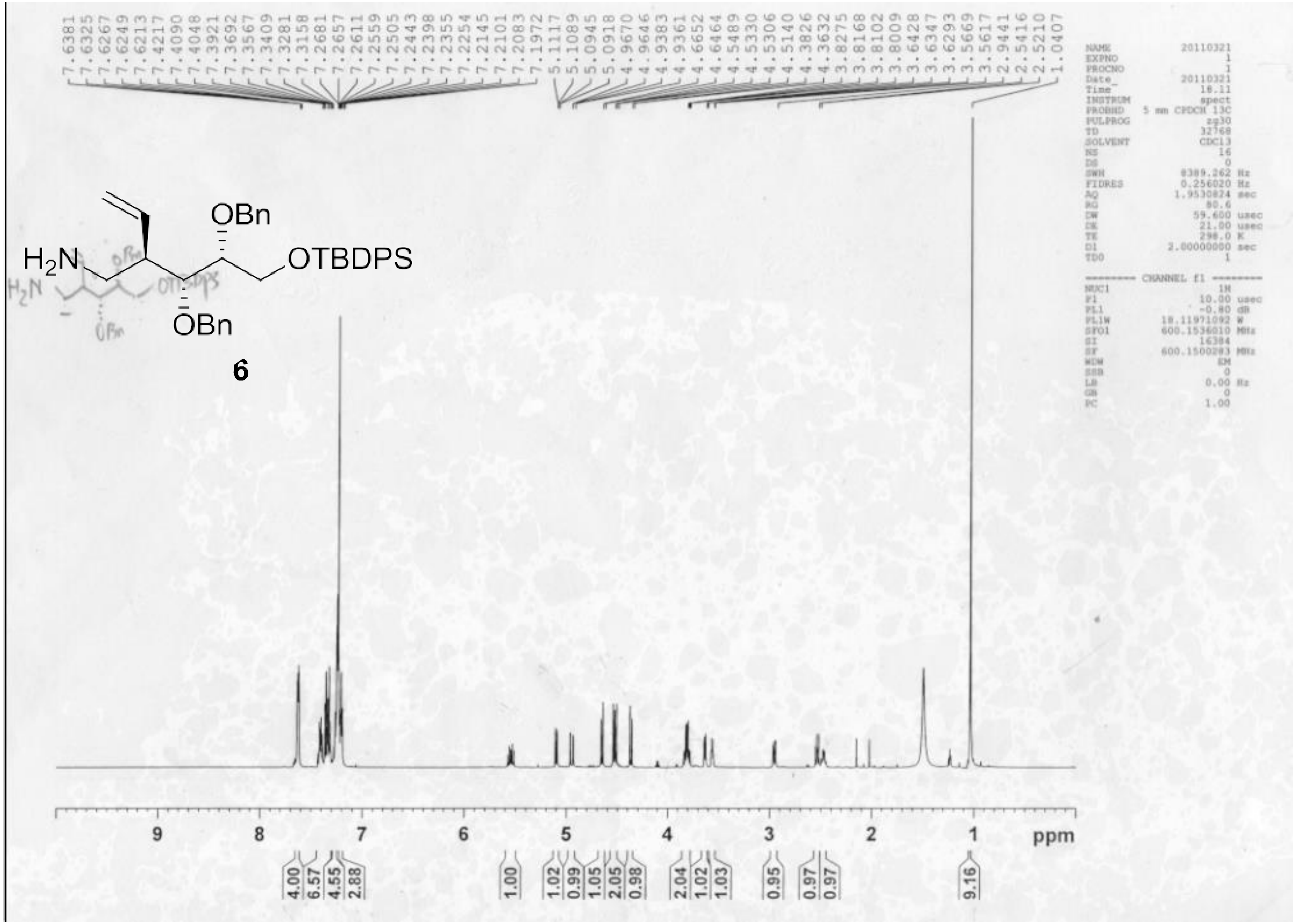

^{1}H spectrum of compound **6 (**600 MHz, $CDCl_3$)

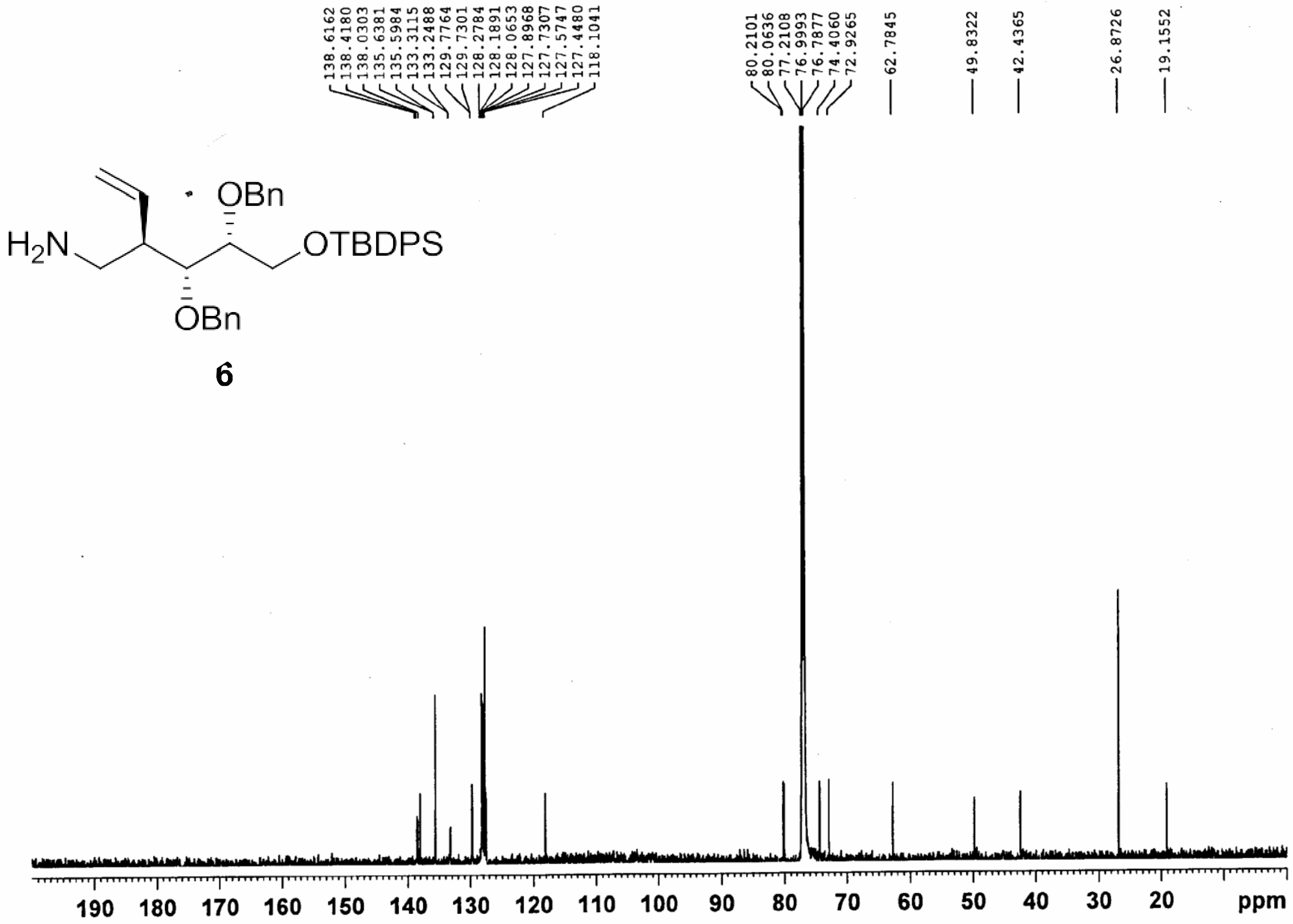

^{13}C spectrum of compound **6** (150 MHz, $CDCl_3$)

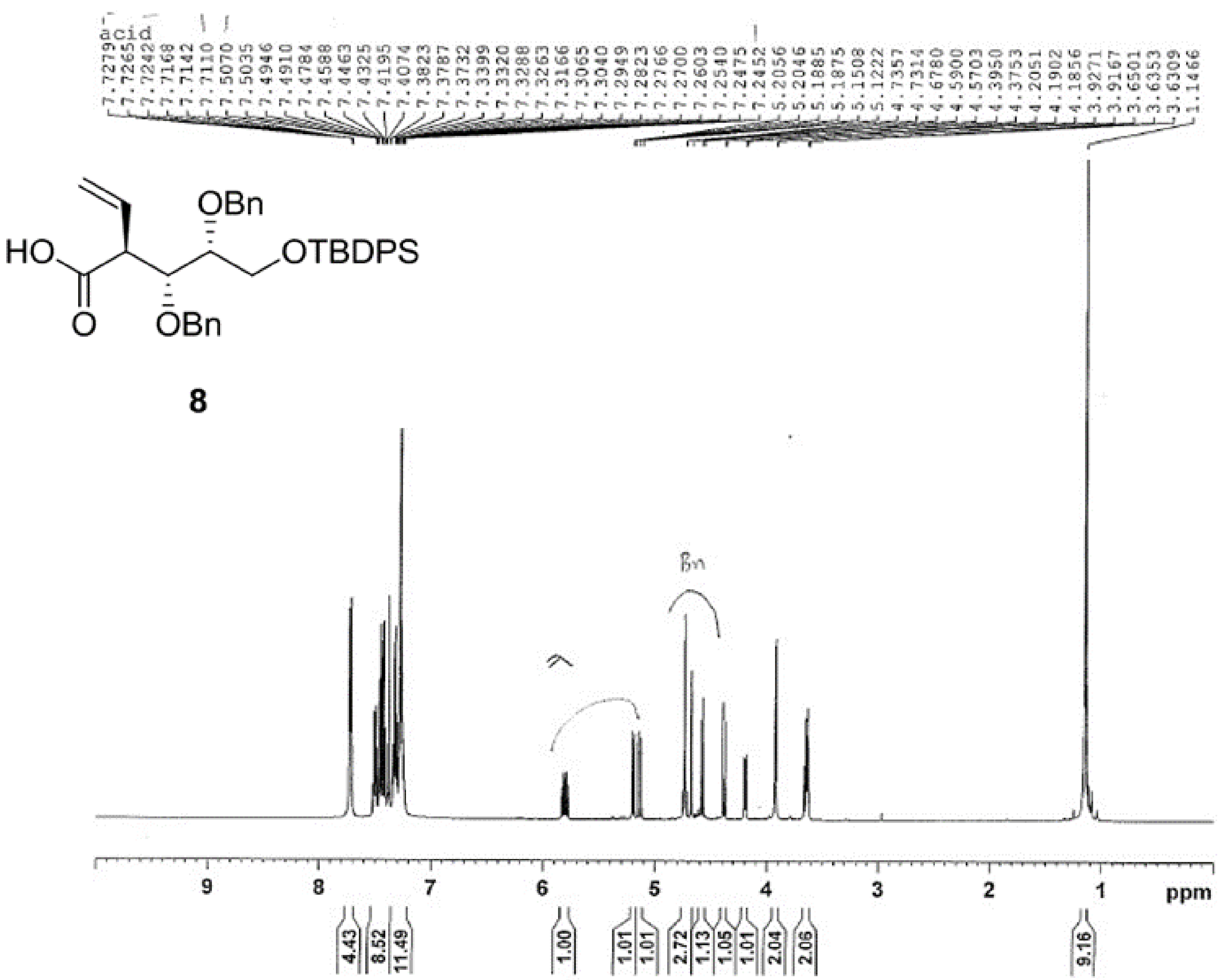

^{1}H spectrum of compound **8 (**600 MHz, $CDCl_3$)

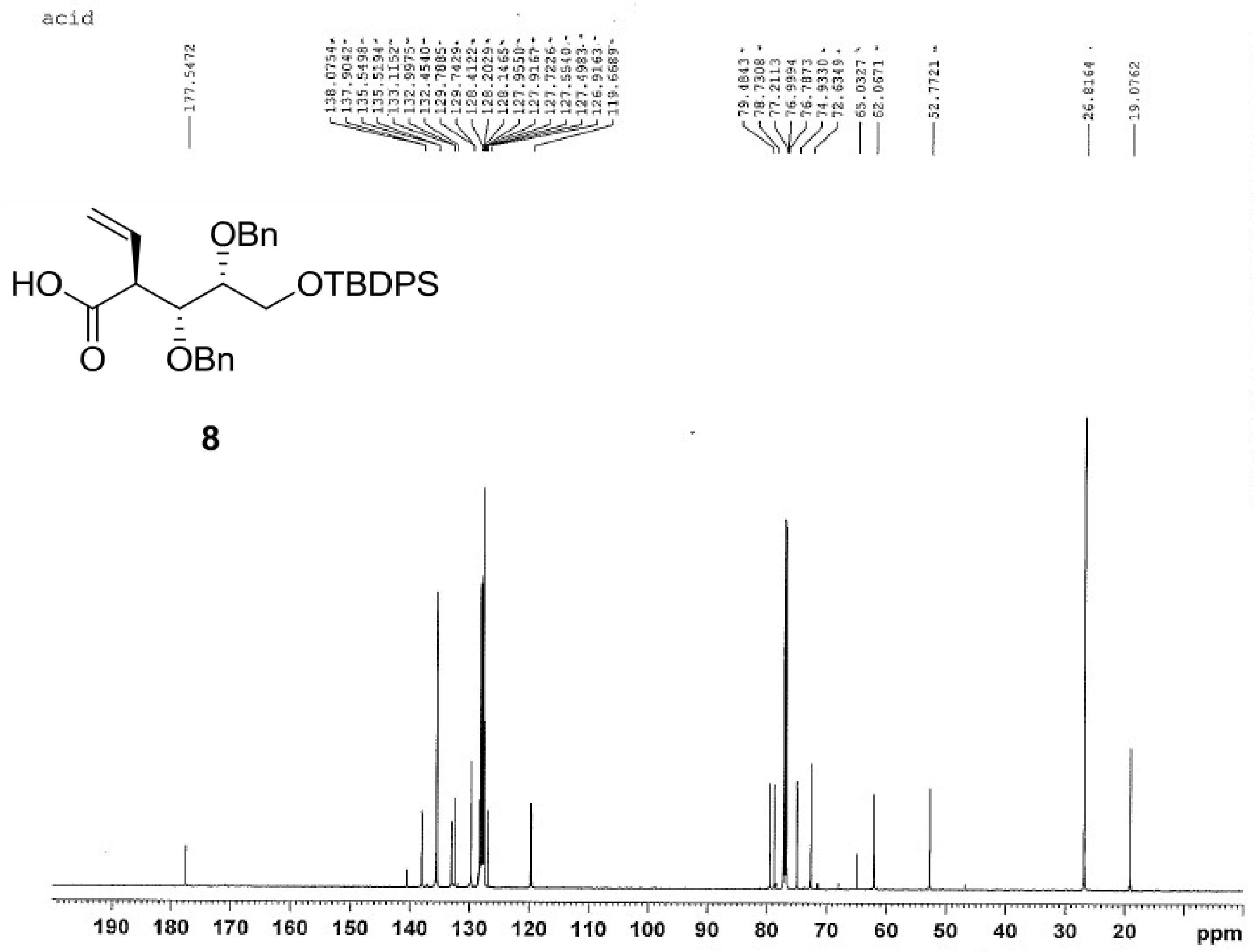

^{13}C spectrum of compound **8 (**150 MHz, $CDCl_3$)

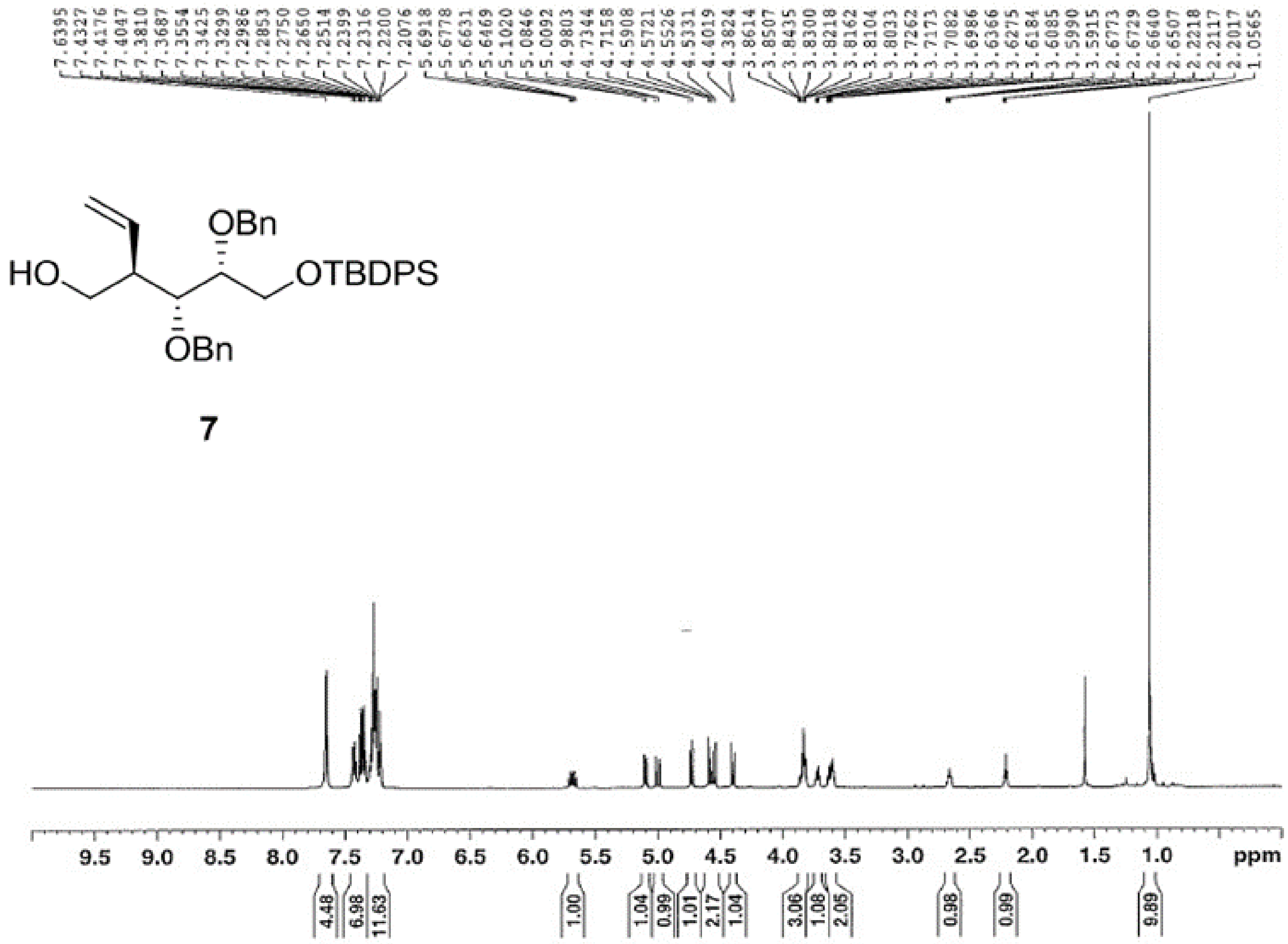

^{1}H spectrum of compound **7 (**600 MHz, $CDCl_3$)

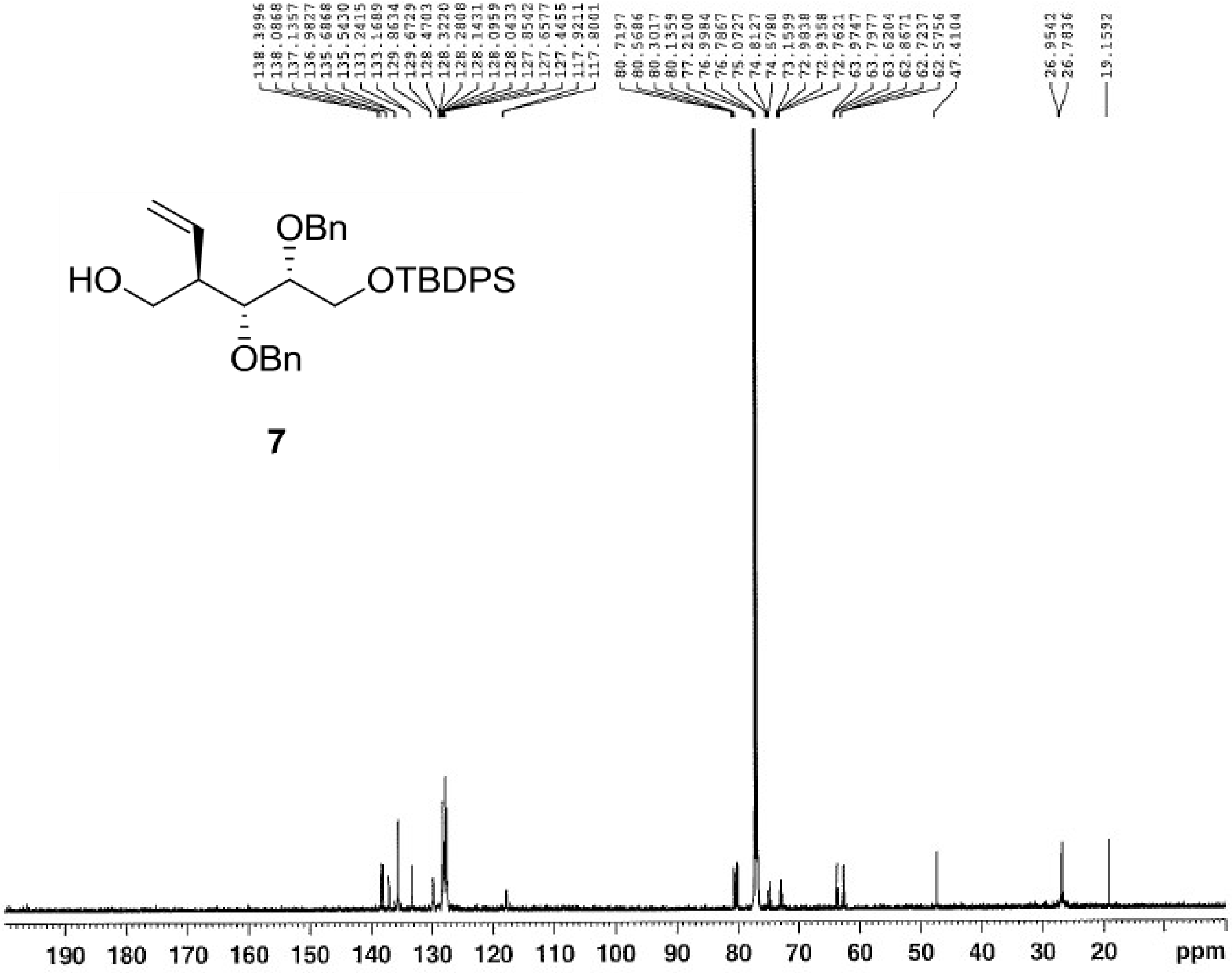

^{13}C spectrum of compound **7 (**150 MHz, $CDCl_3$)

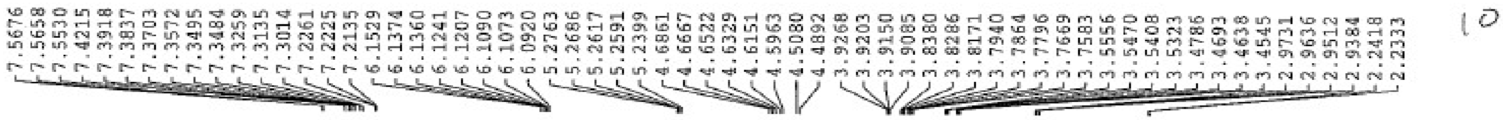

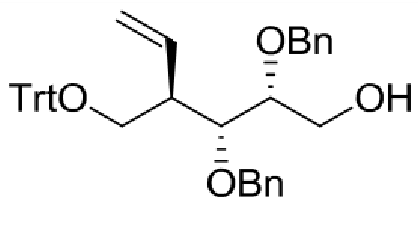

9

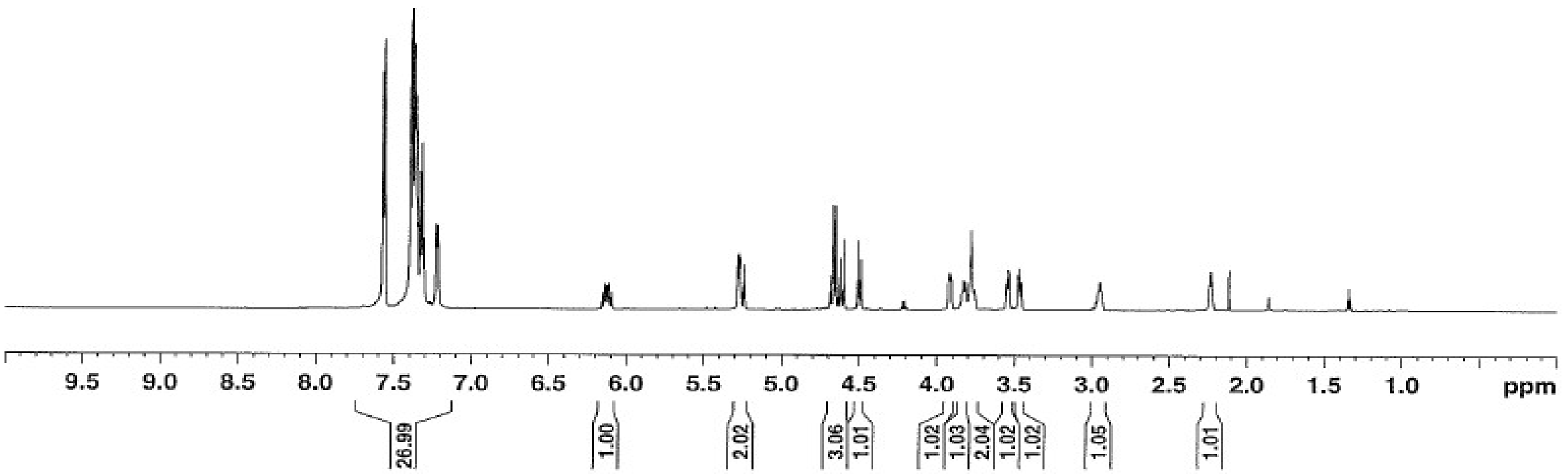

^{1}H spectrum of compound **9** (600 MHz, $CDCl_3$)

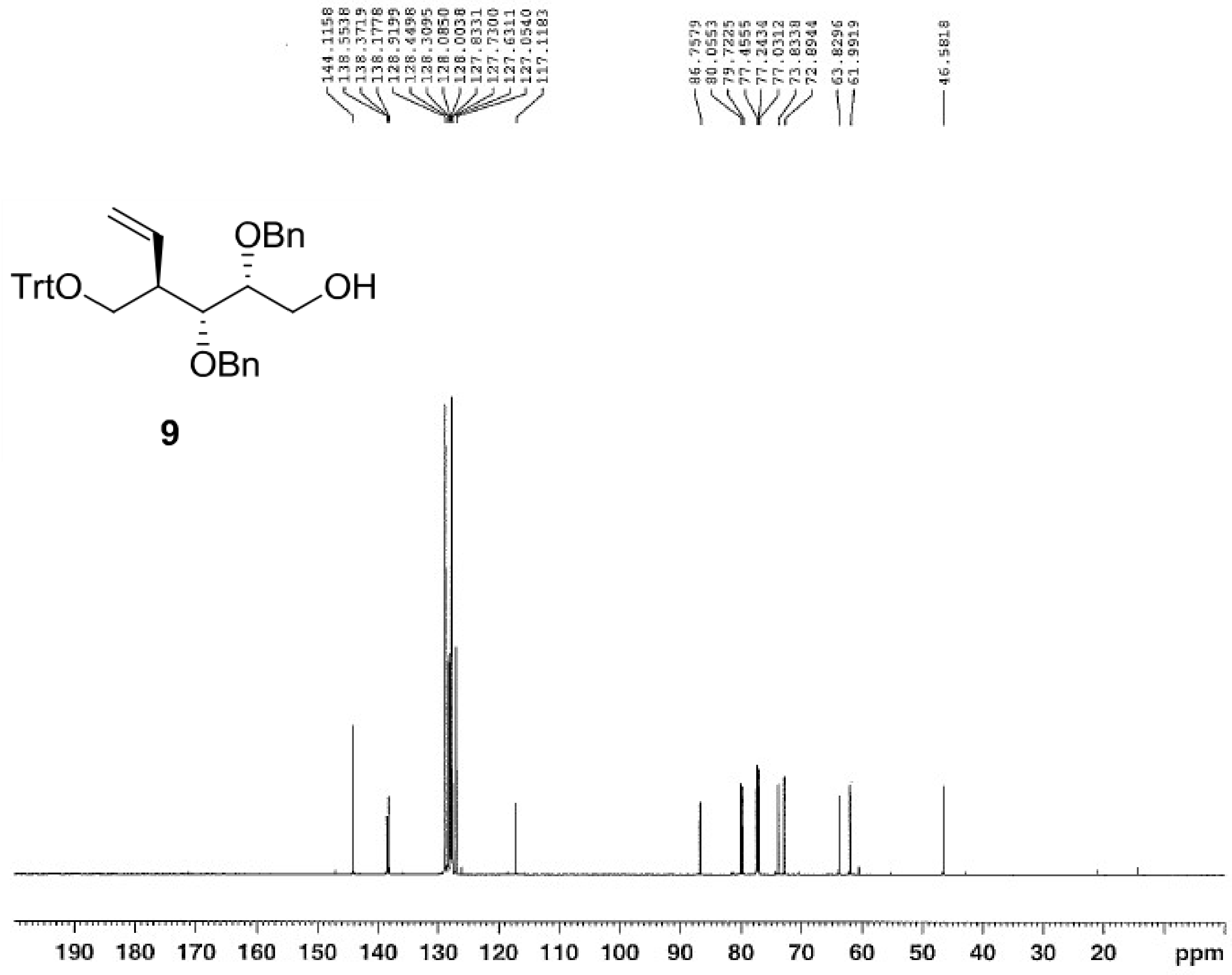

^{13}C spectrum of compound **9** (150 MHz, $CDCl_3$)

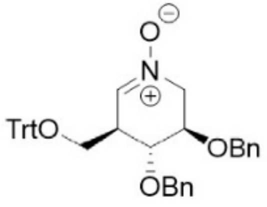

1

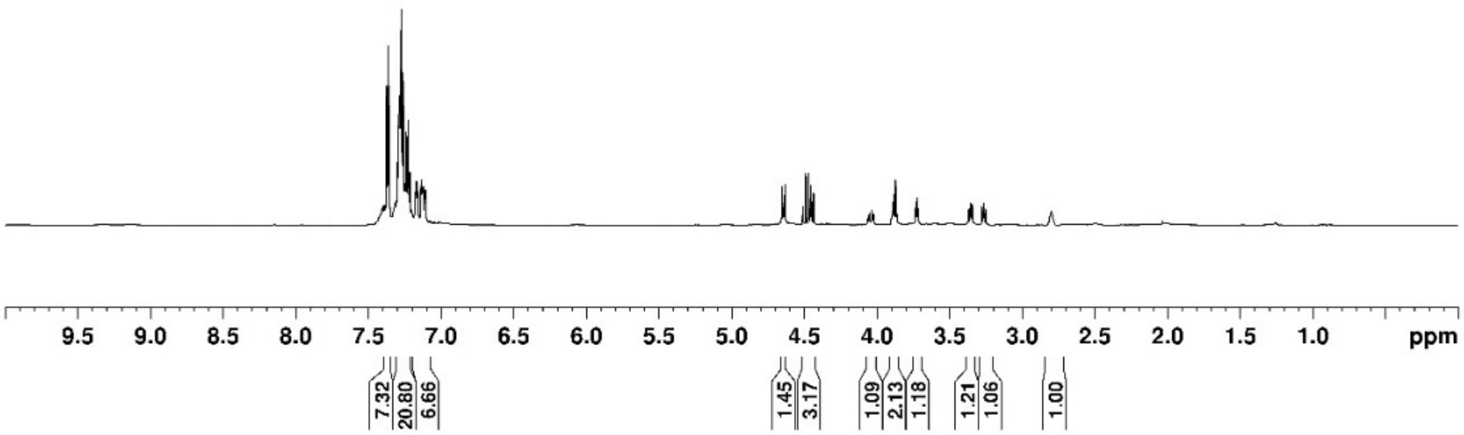

^{1}H spectrum of compound **1 (**600 MHz, $CDCl_3$)

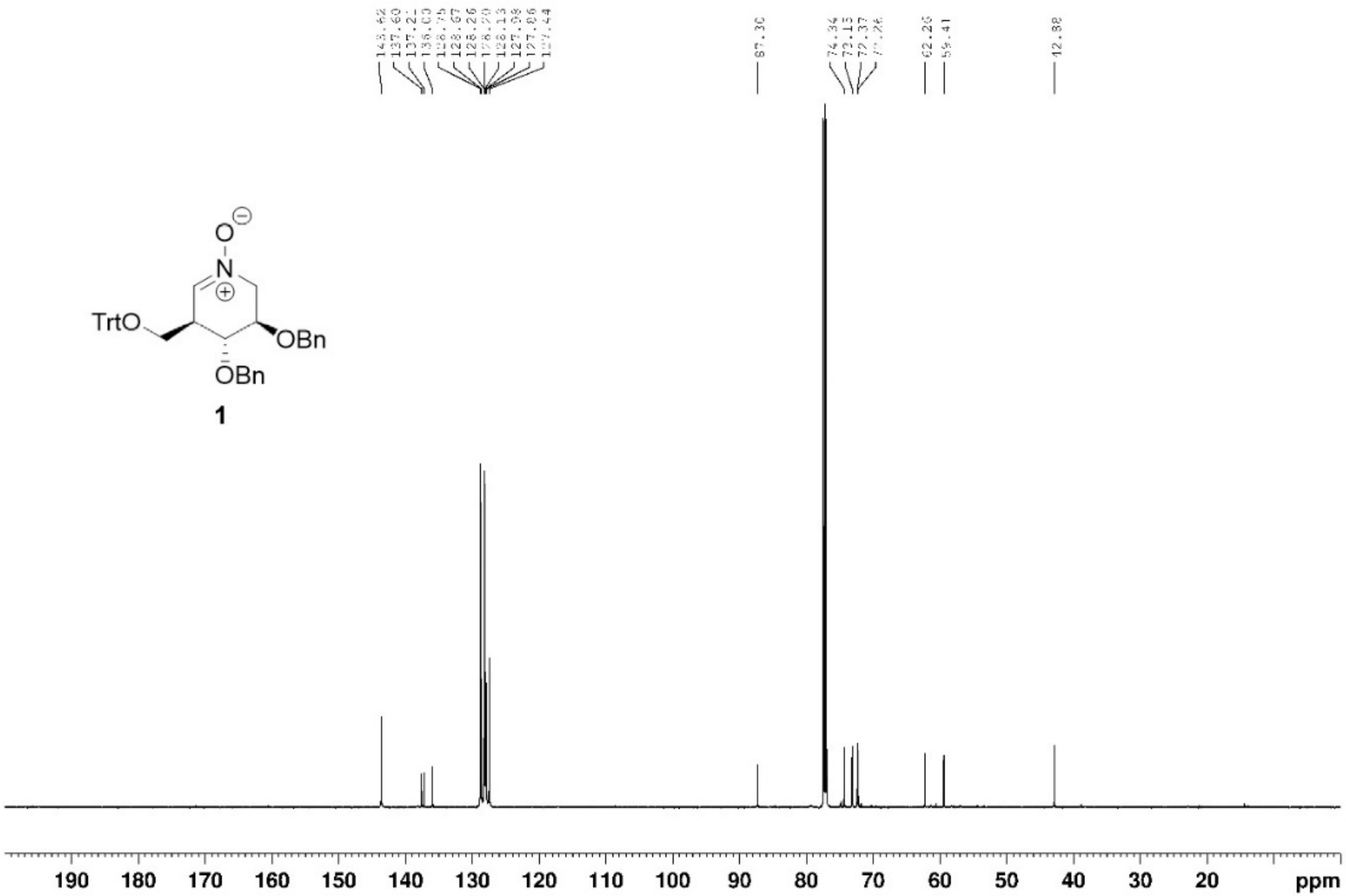

^{13}C spectrum of compound **1** (150 MHz, $CDCl_3$)

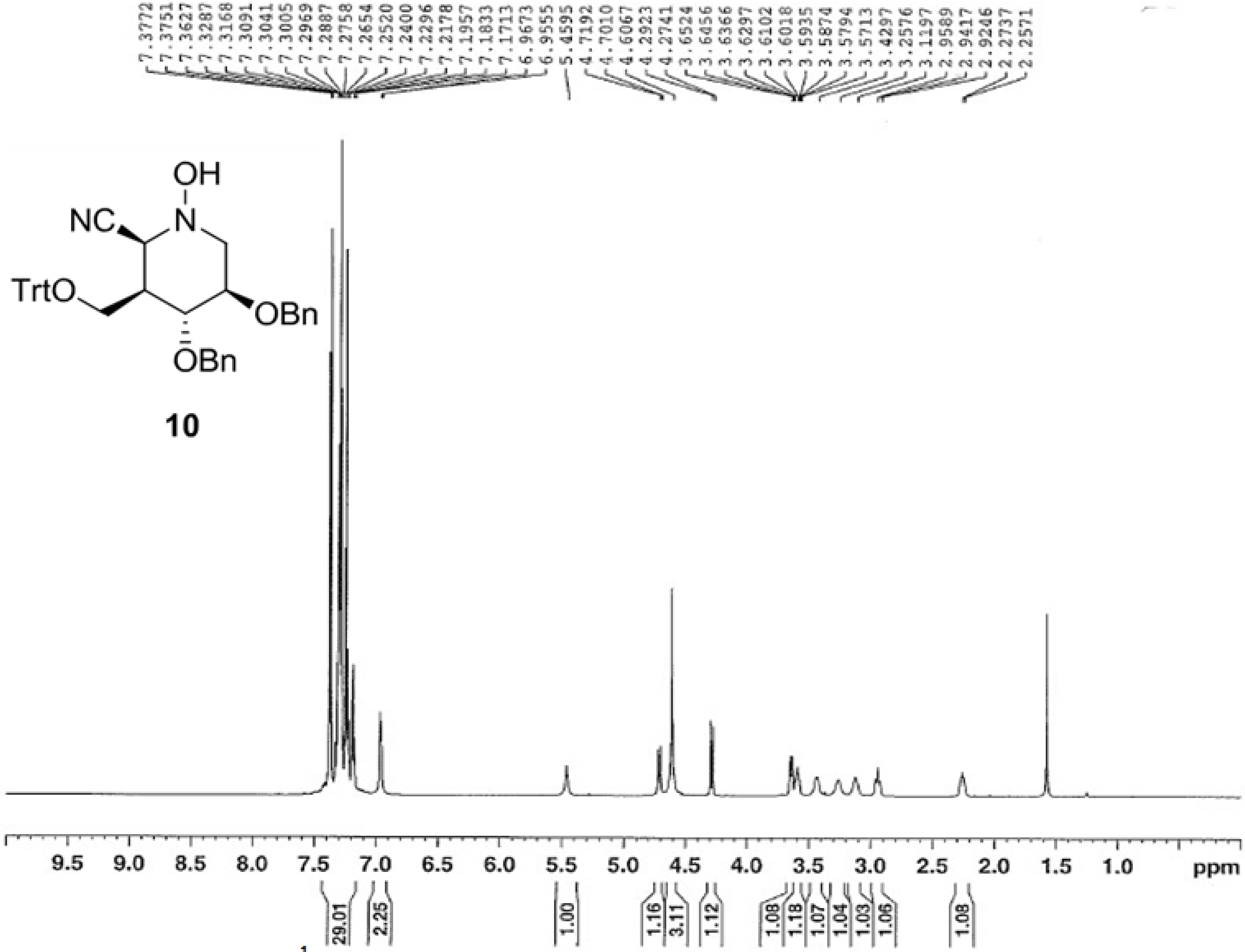

^{1}H spectrum of compound **10** (600 MHz, $CDCl_3$)

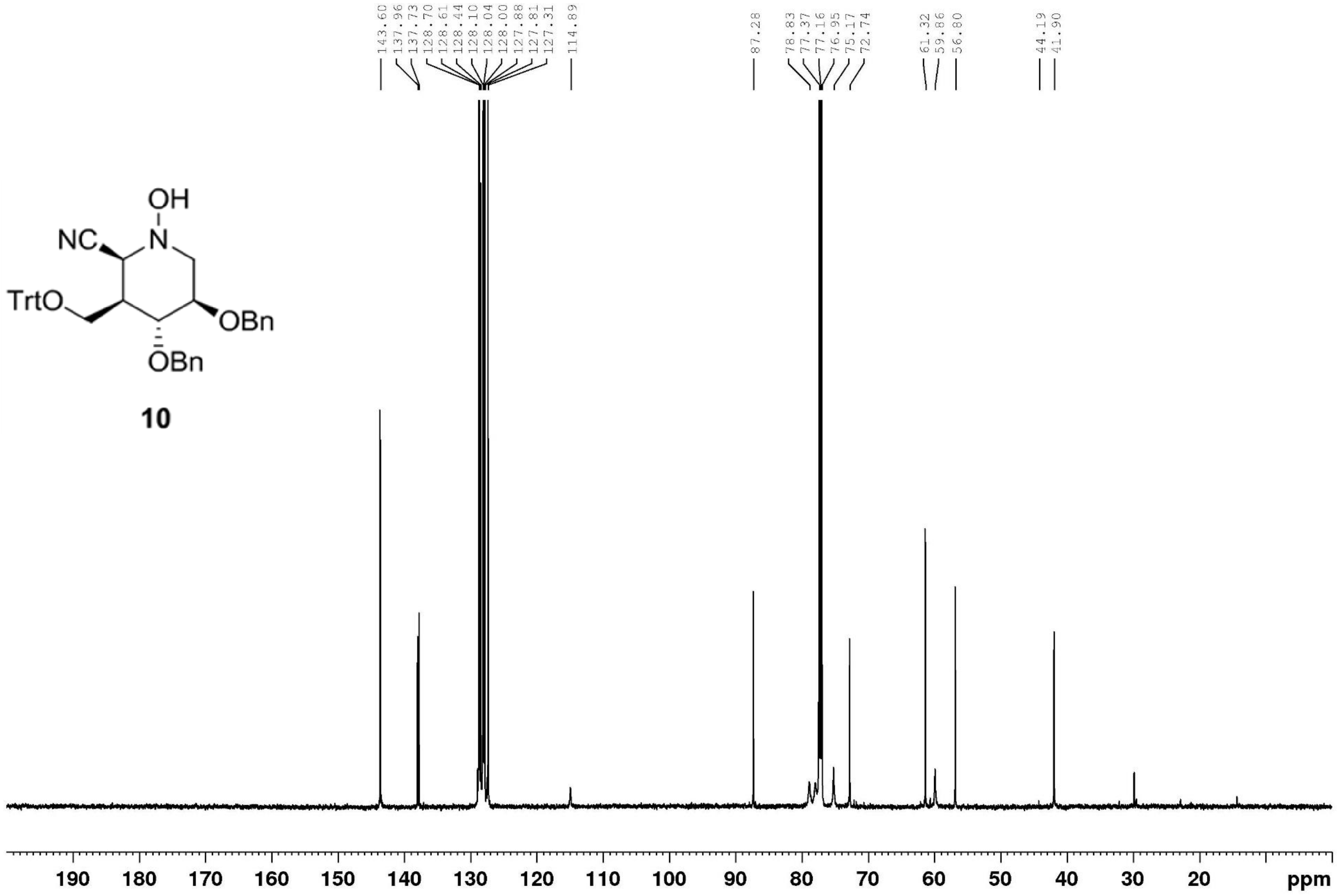

^{13}C spectrum of compound **10** (150 MHz, $CDCl_3$)

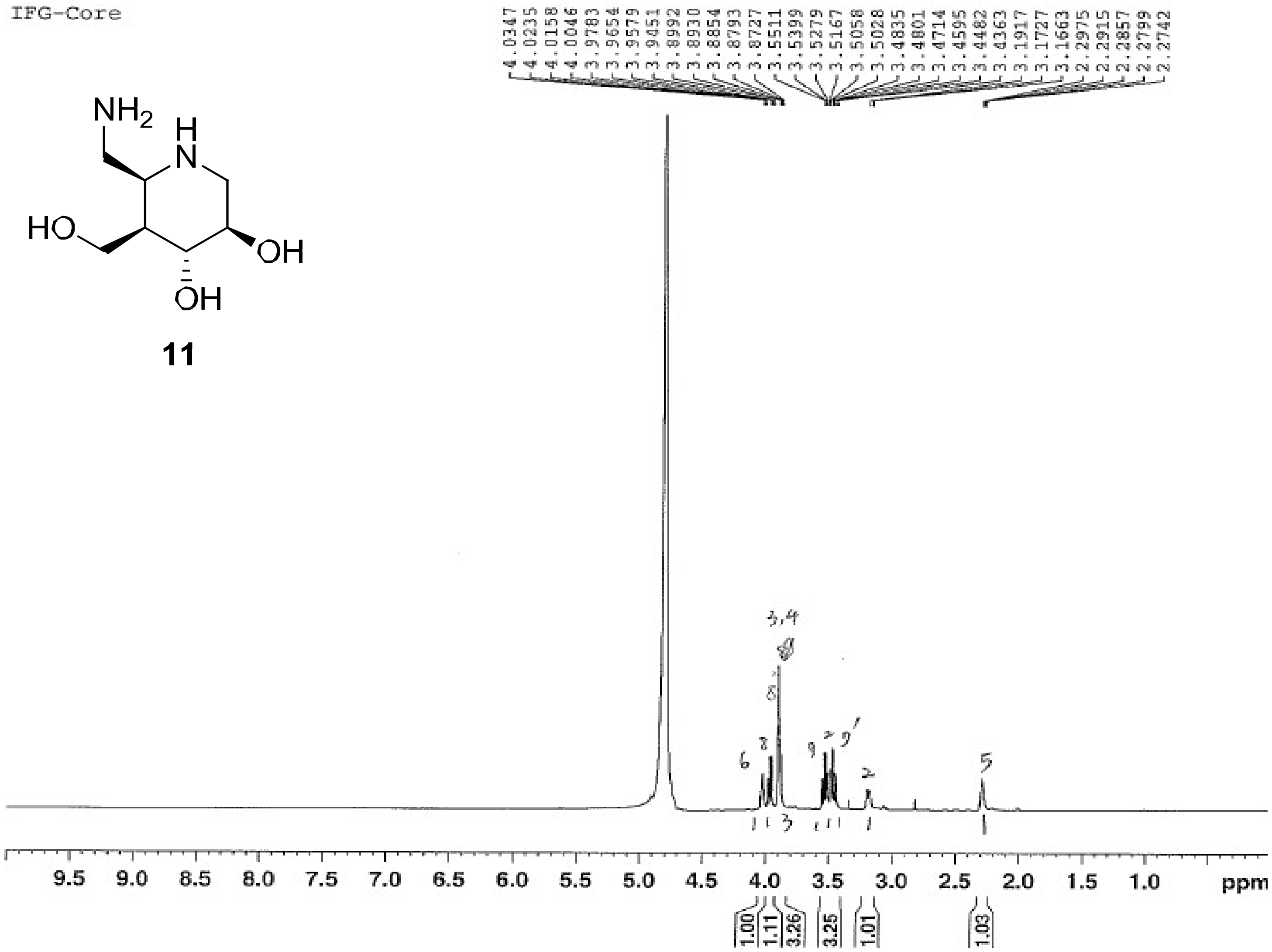

^{1}H spectrum of compound **11** (600 MHz, D_2O)

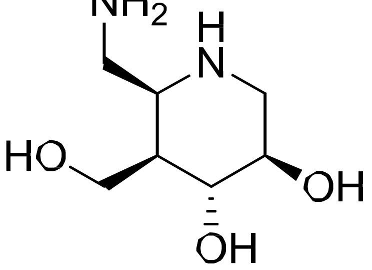

11

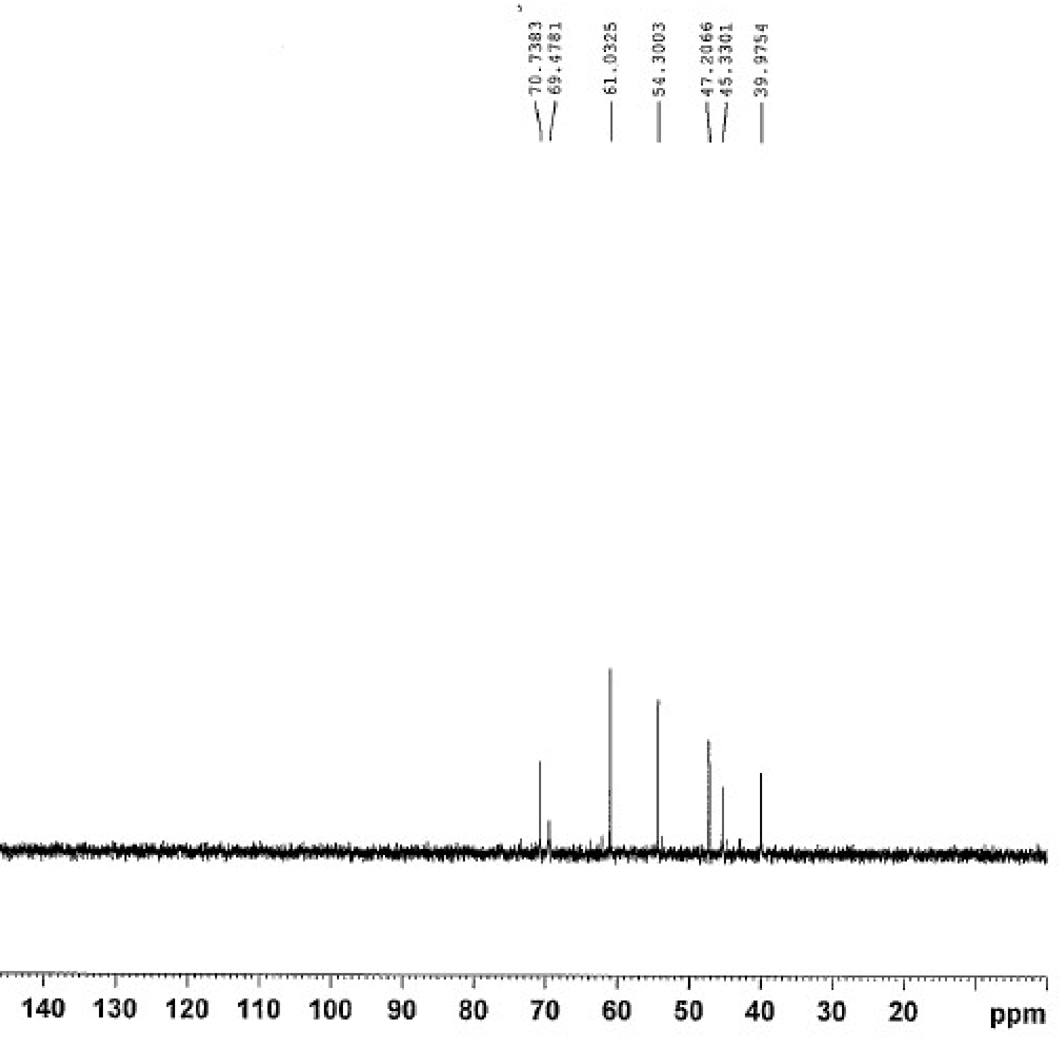

^{13}C spectrum of compound **11** (150 MHz, D_2O)

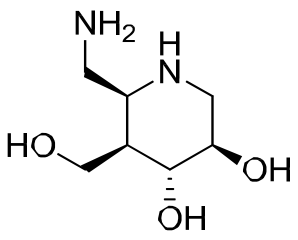

11

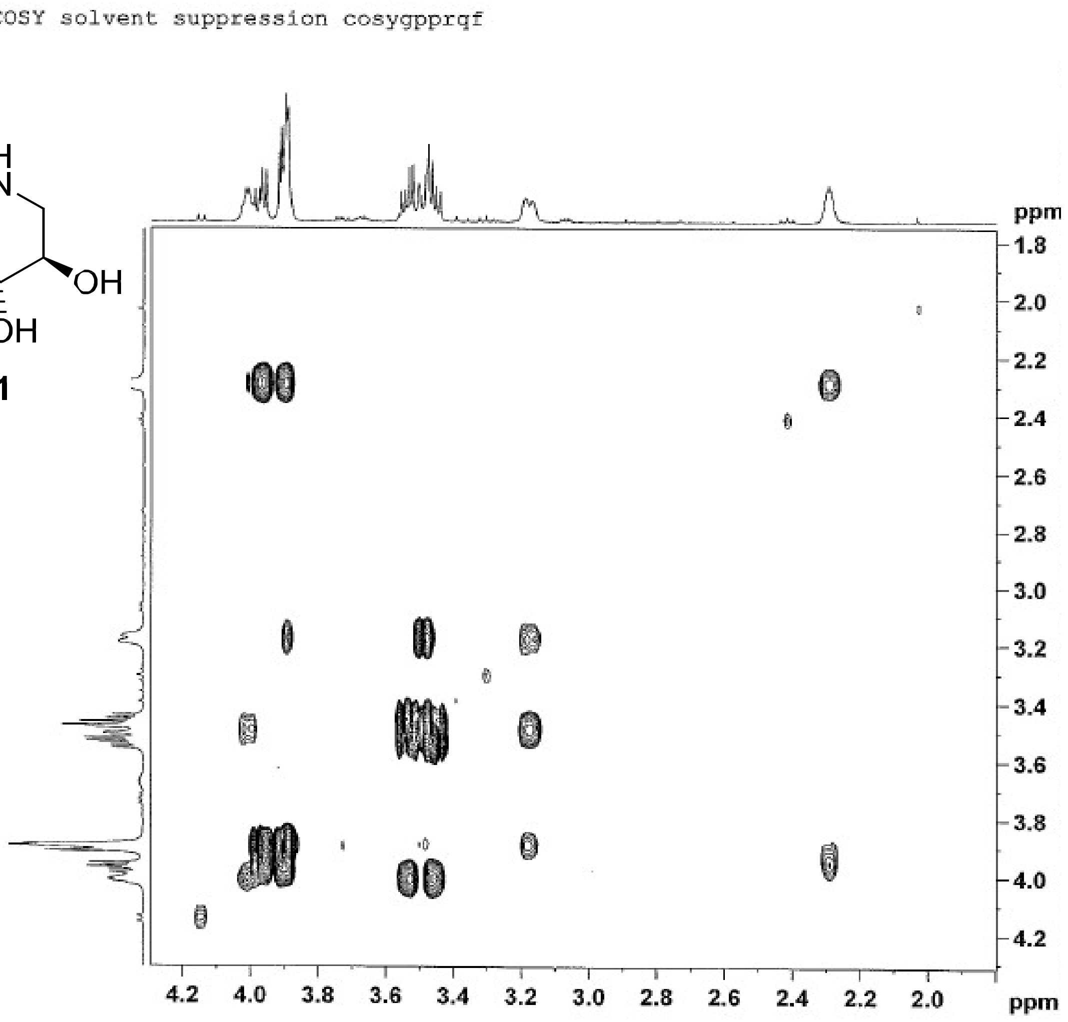

COSY of compound **11 (**600 MHz, D_2O)

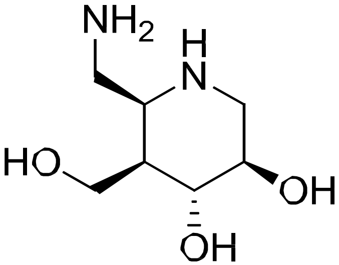

11

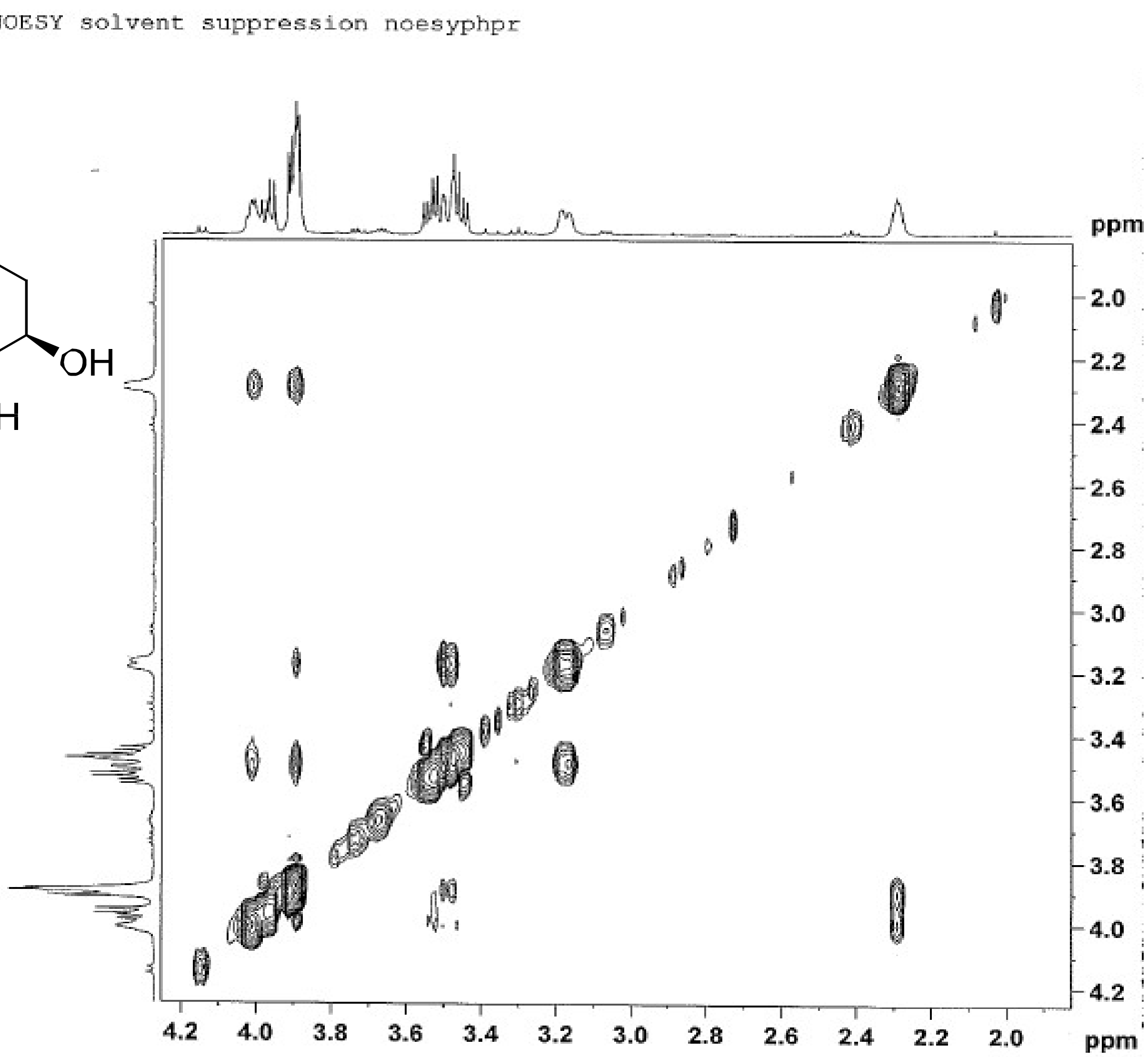

NOESY of compound **11 (**600 MHz, D_2O)

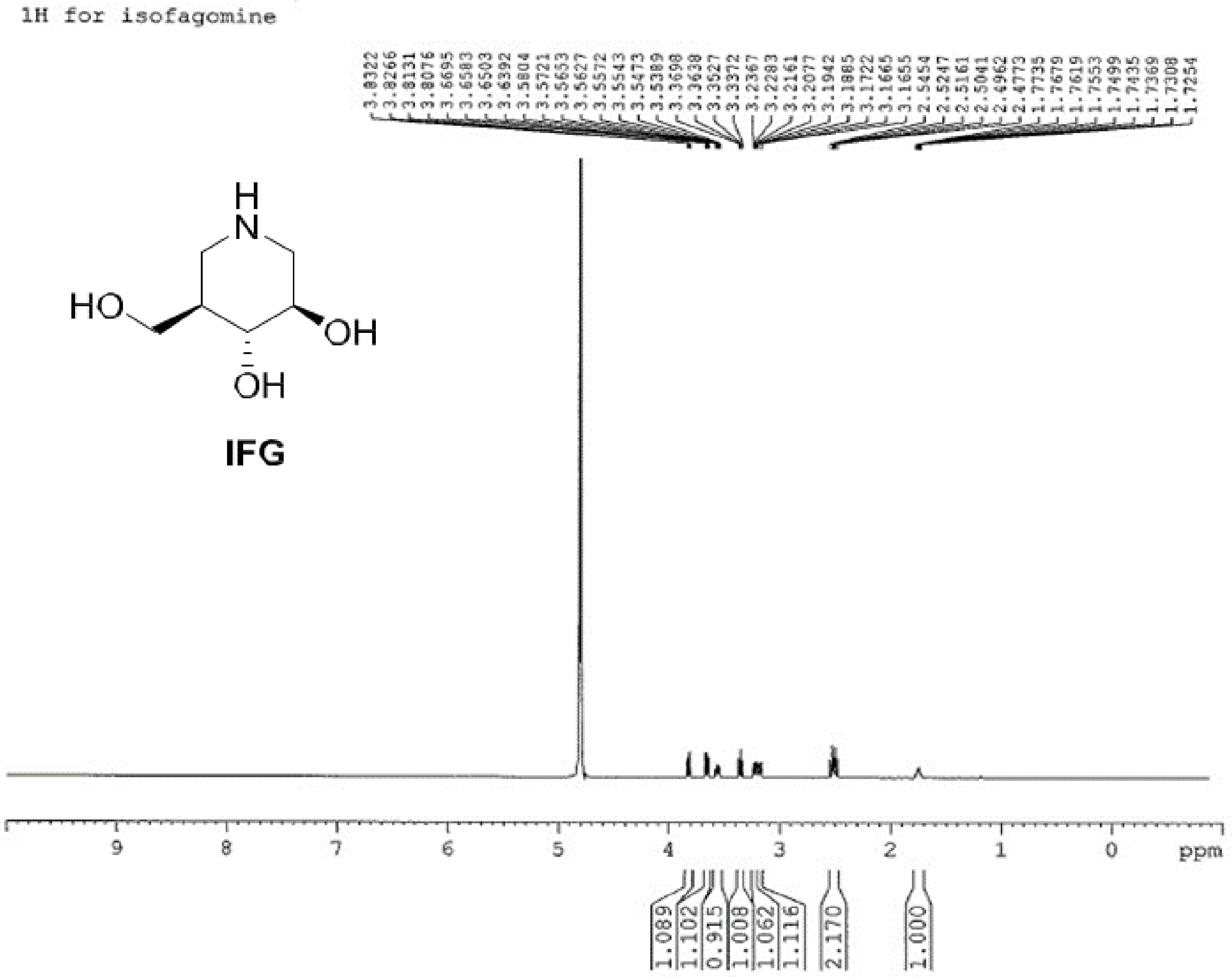

^{1}H spectrum of IFG (600 MHz, D_2O)

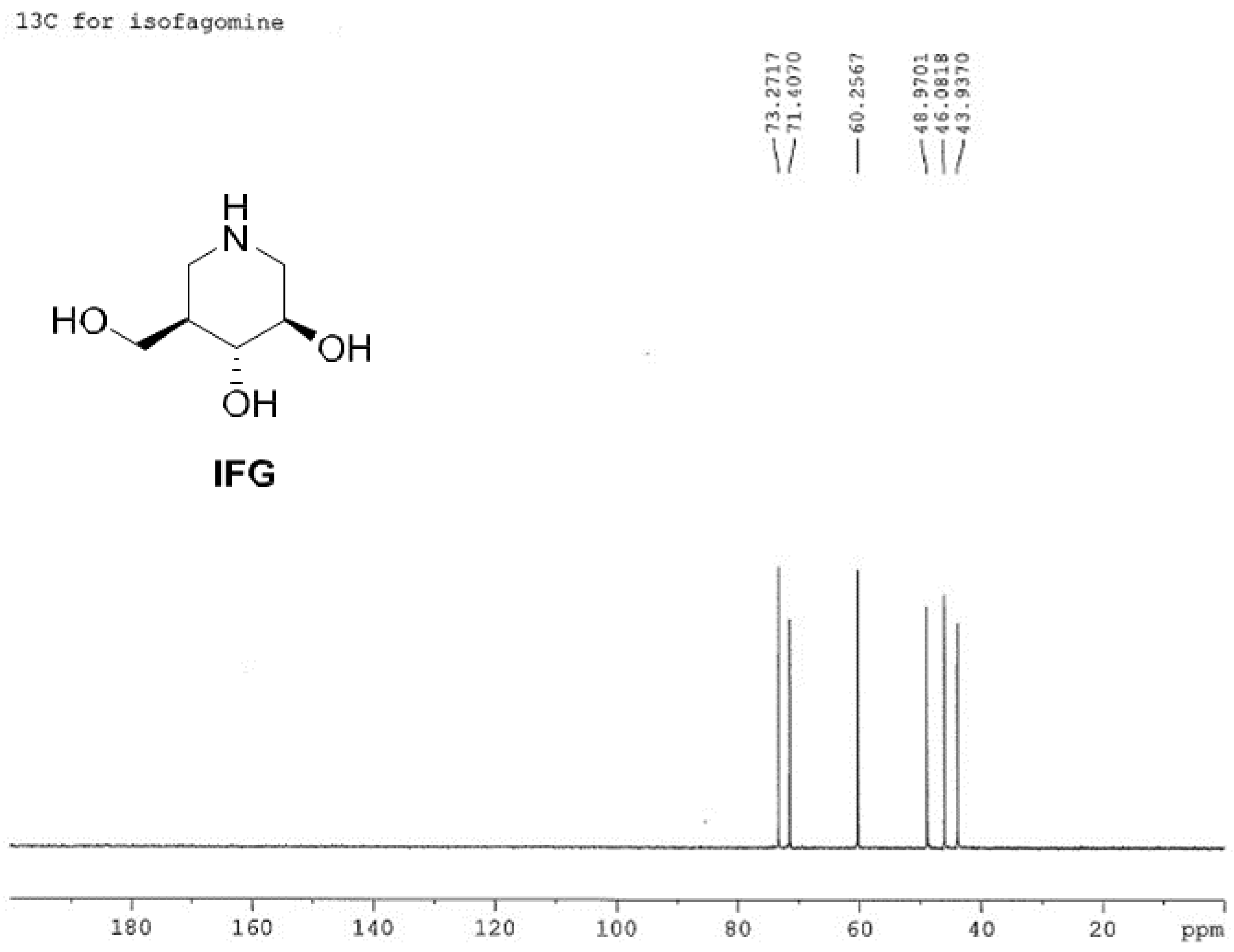

^{13}C spectrum of IFG (150 MHz, D_2O)

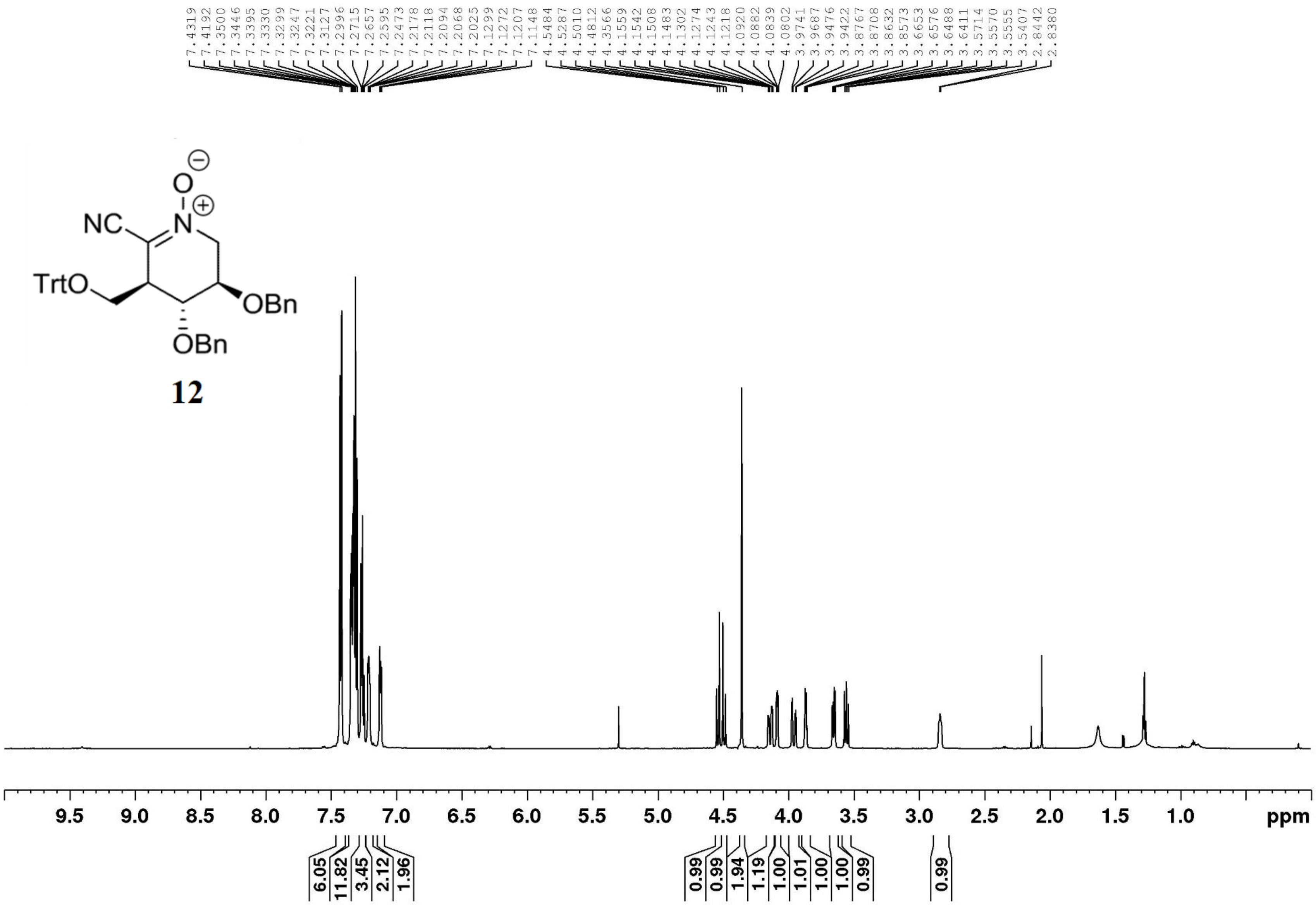

^{1}H spectrum of compound **12** (600 MHz, $CDCl_3$)

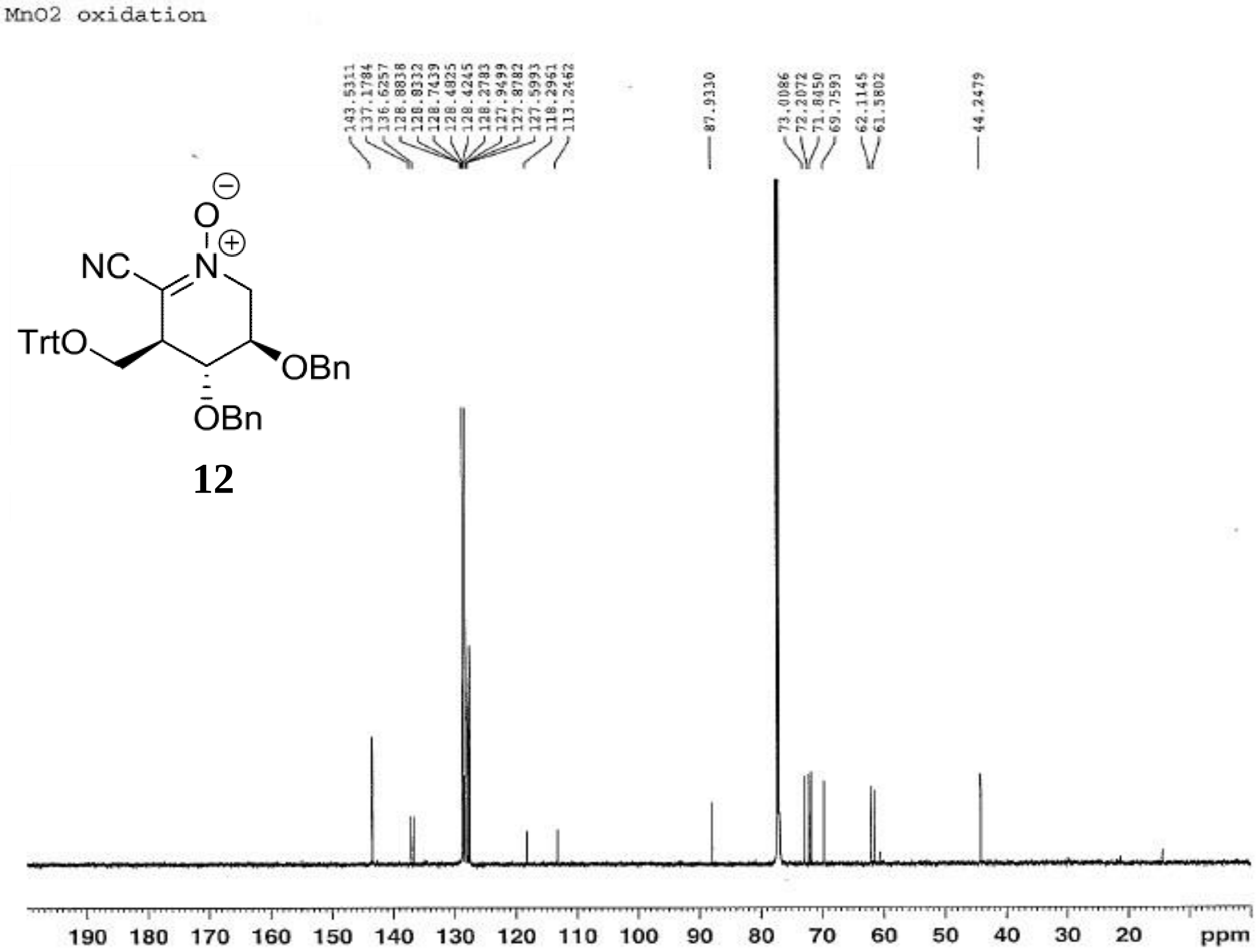

^{13}C spectrum of compound **12 (**150 MHz, $CDCl_3$)

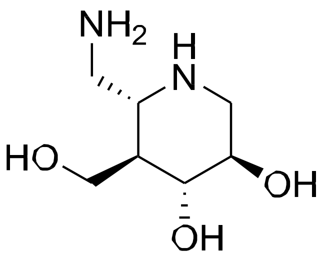

14

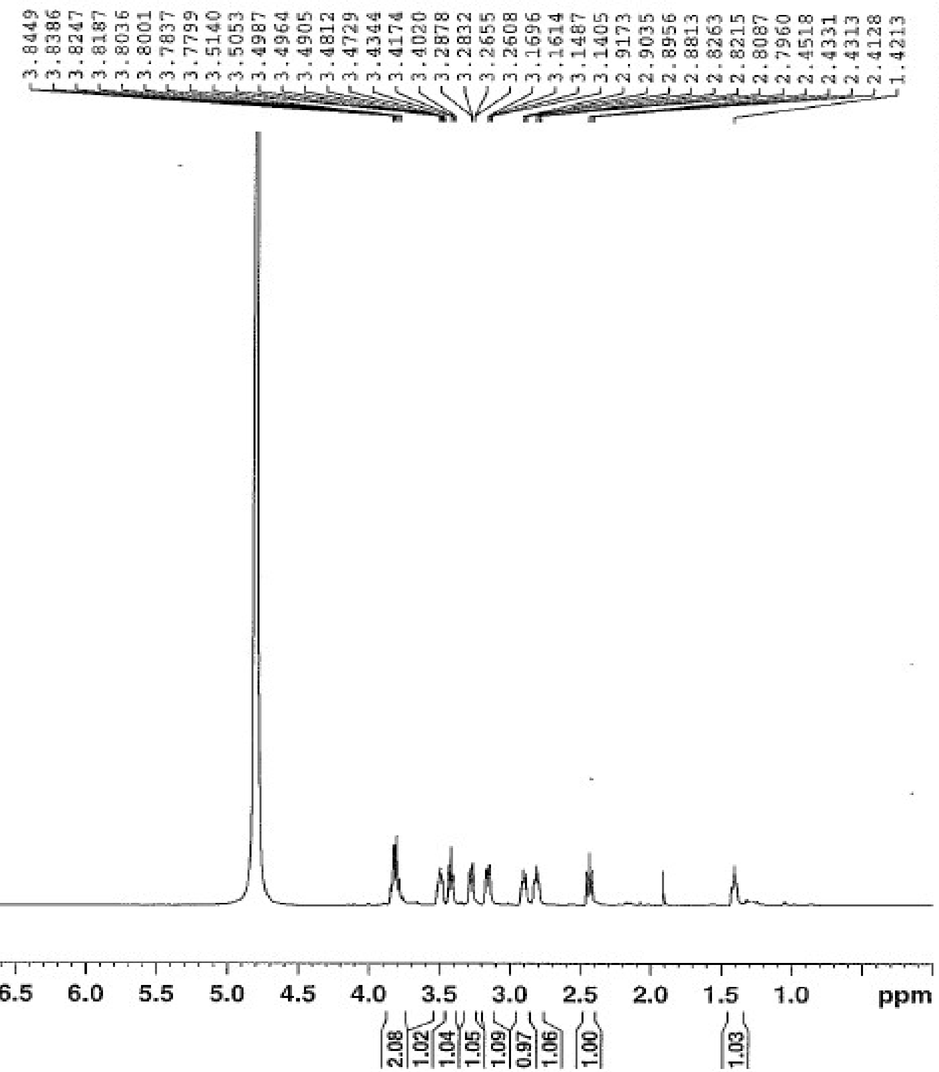

^{1}H spectrum of compound **14 (**600 MHz, D_2O)

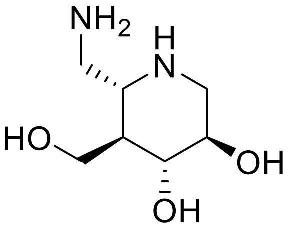

14

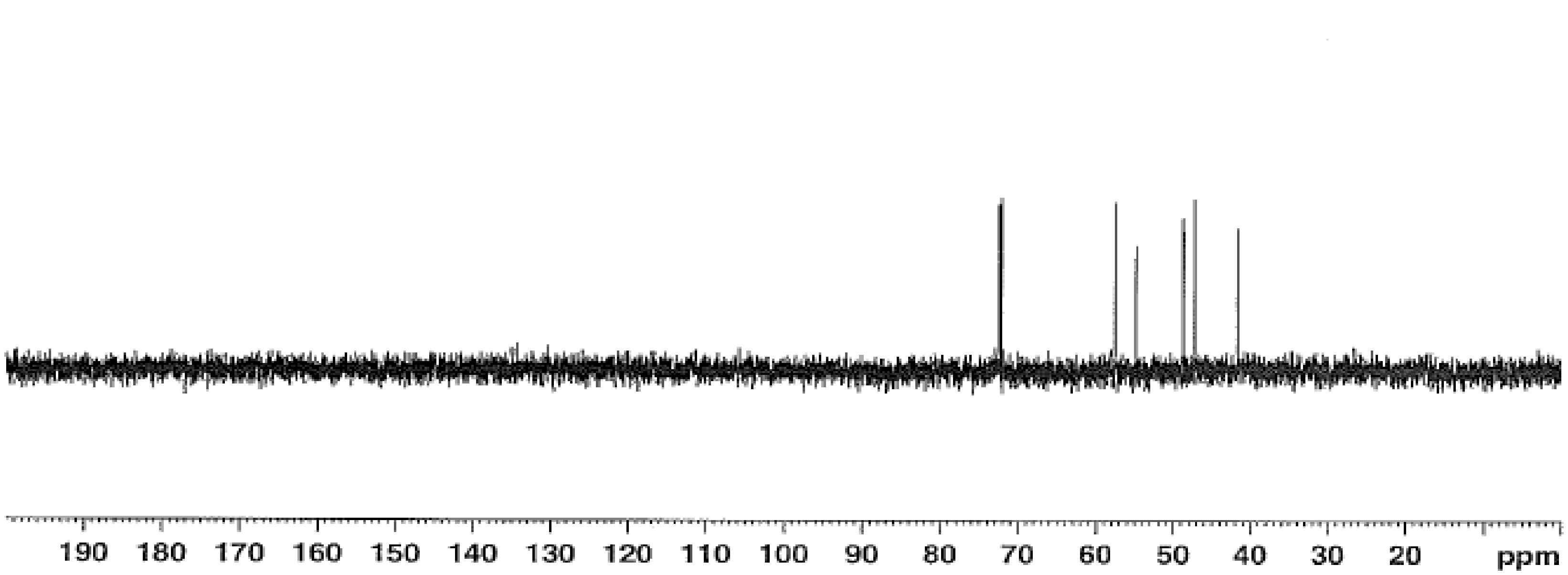

^{1}H spectrum of compound **14** (150 MHz, D_2O)

2D COSY solvent suppression cosygpprqf

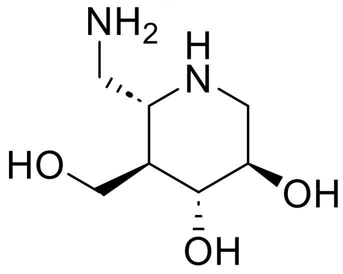

14

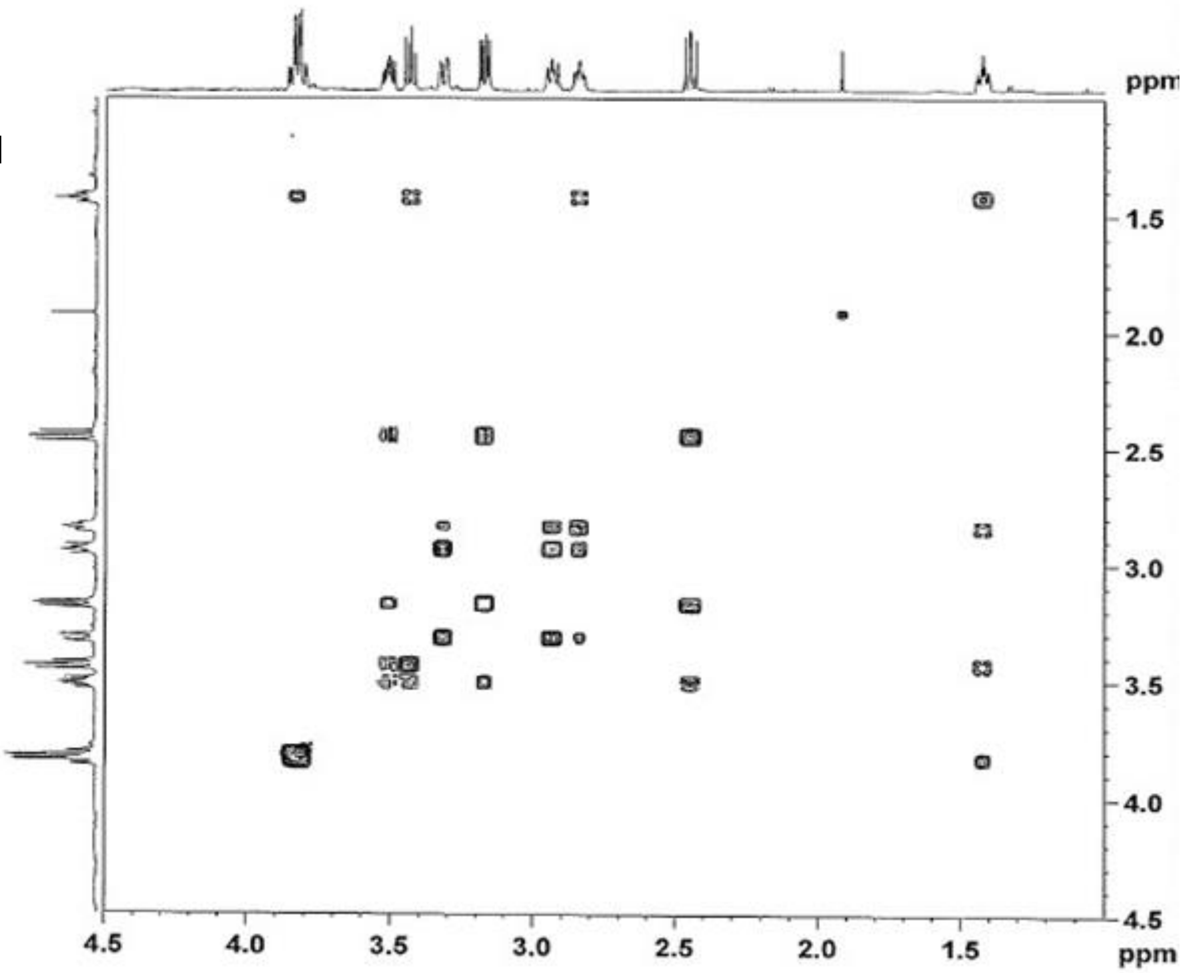

COSY of compound **14** (600 MHz, D_2O)

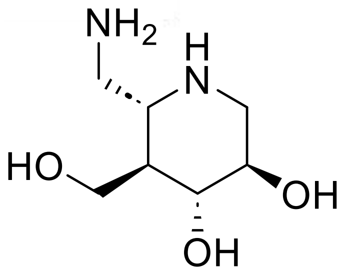

14

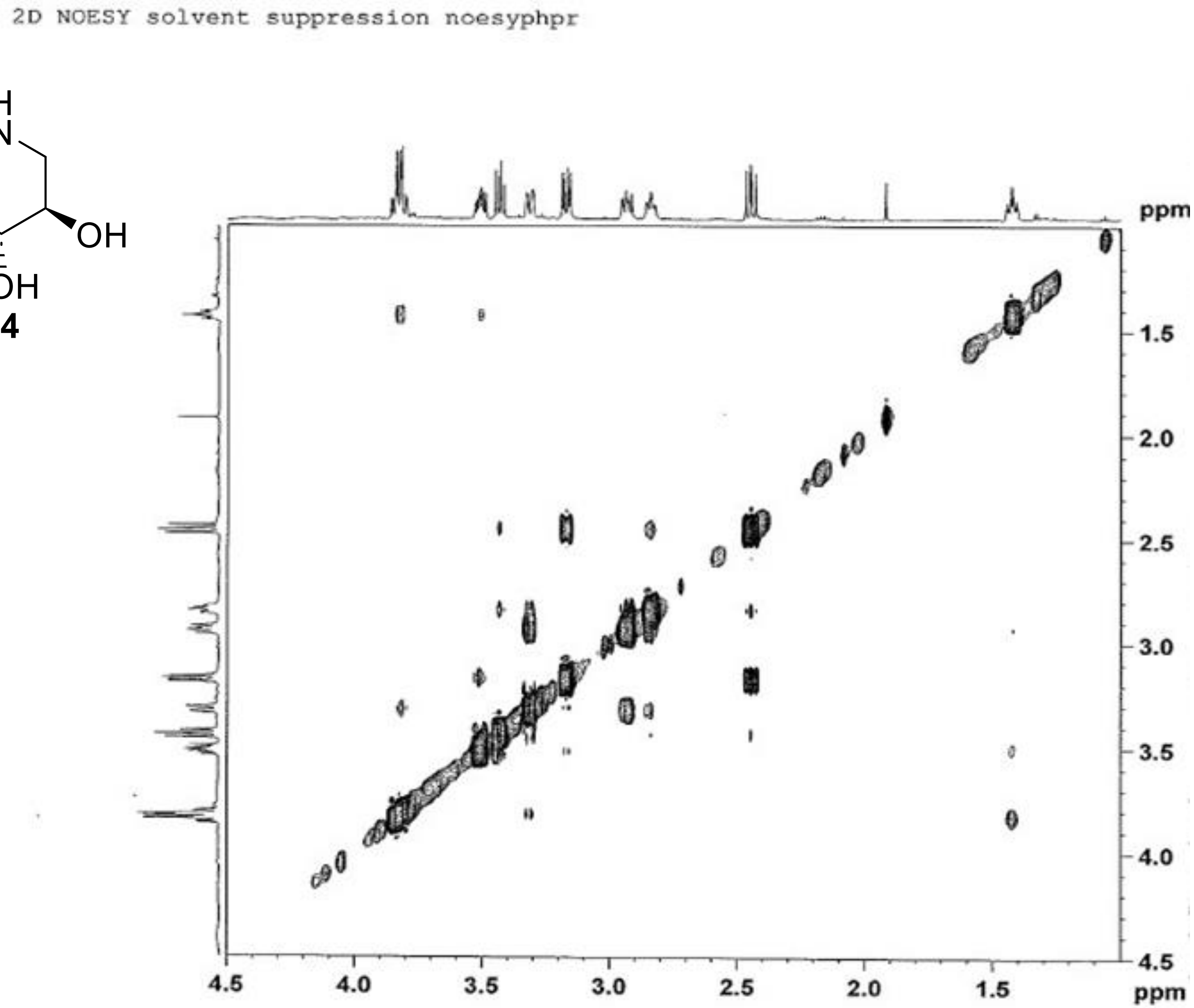

NOESY of compound **14** (600 MHz, D_2O)

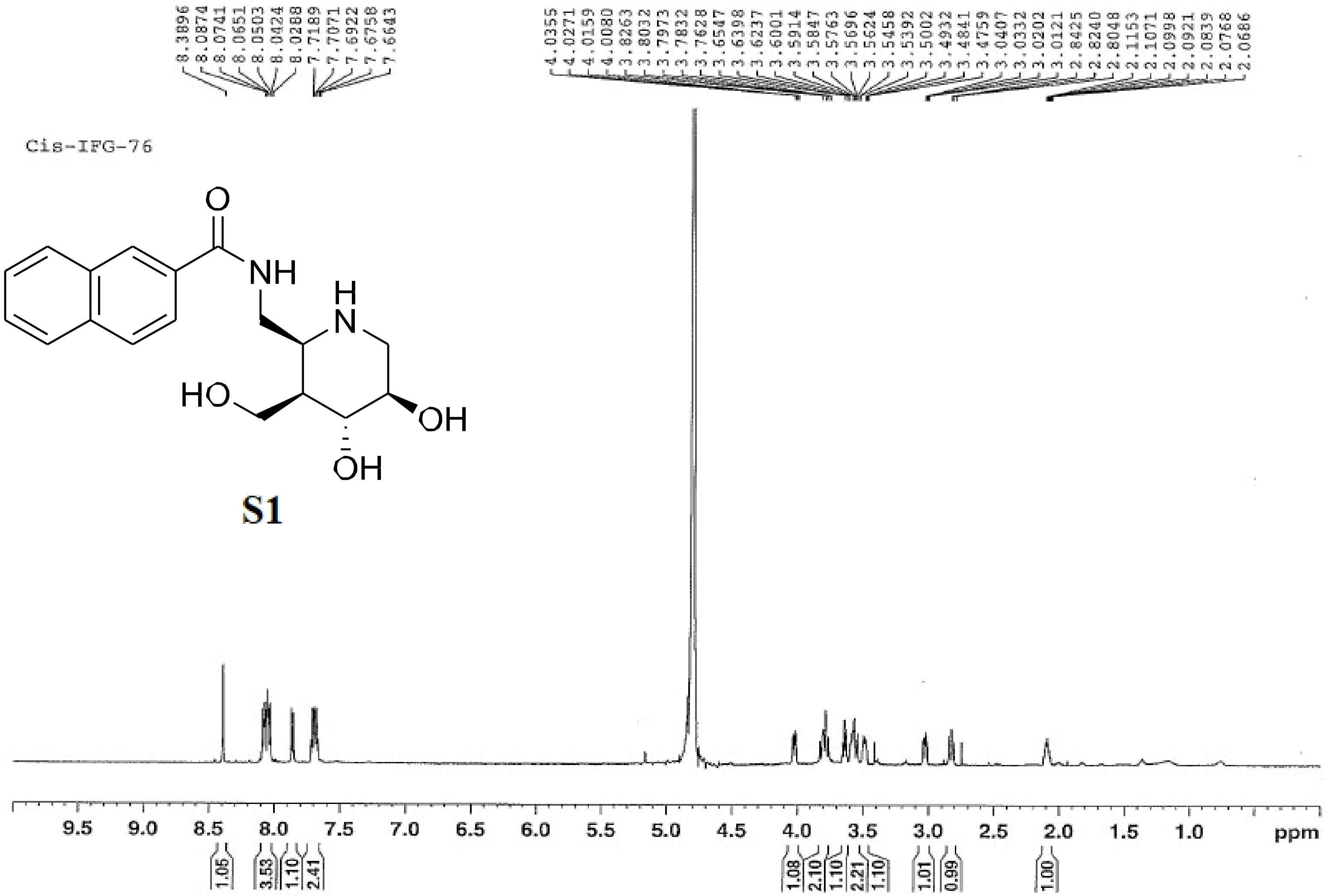

^{1}H spectrum of compound **S1 (**600 MHz, D_2O)

IFG 96

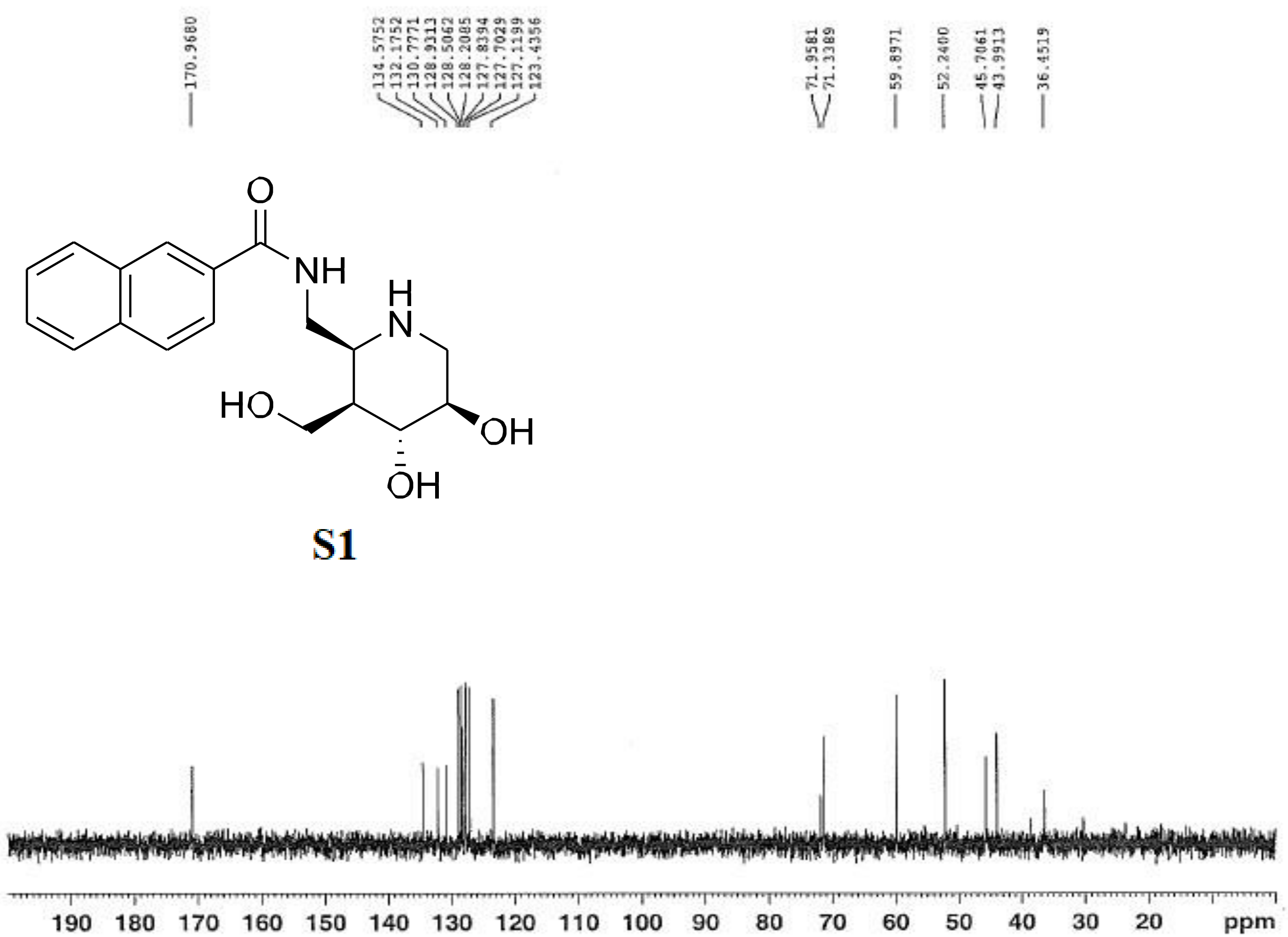

S1

^{13}C spectrum of compound **S1 (**150 MHz, D_2O)

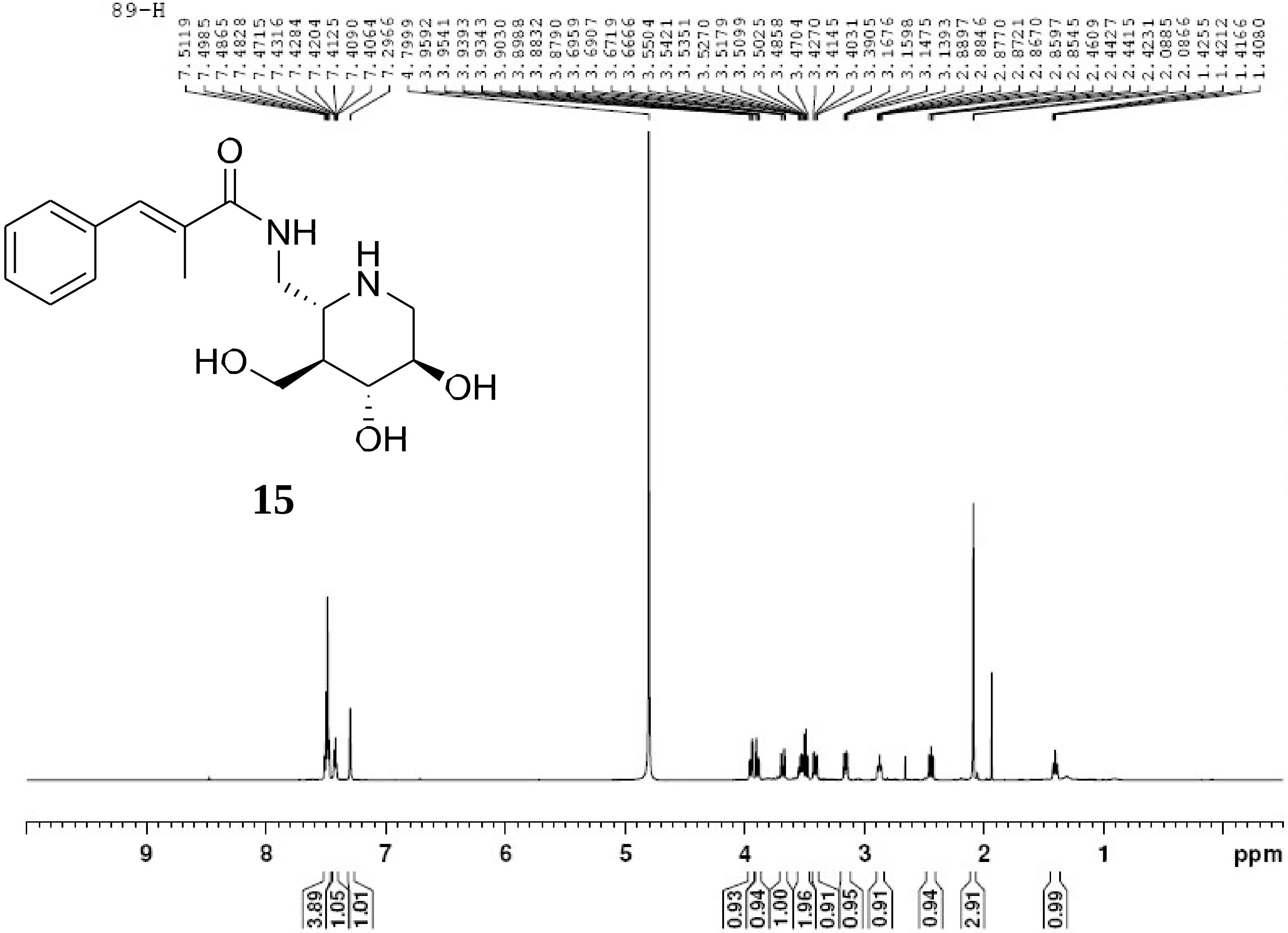

15

^{1}H spectrum of compound **15 (**600 MHz, D_2O)

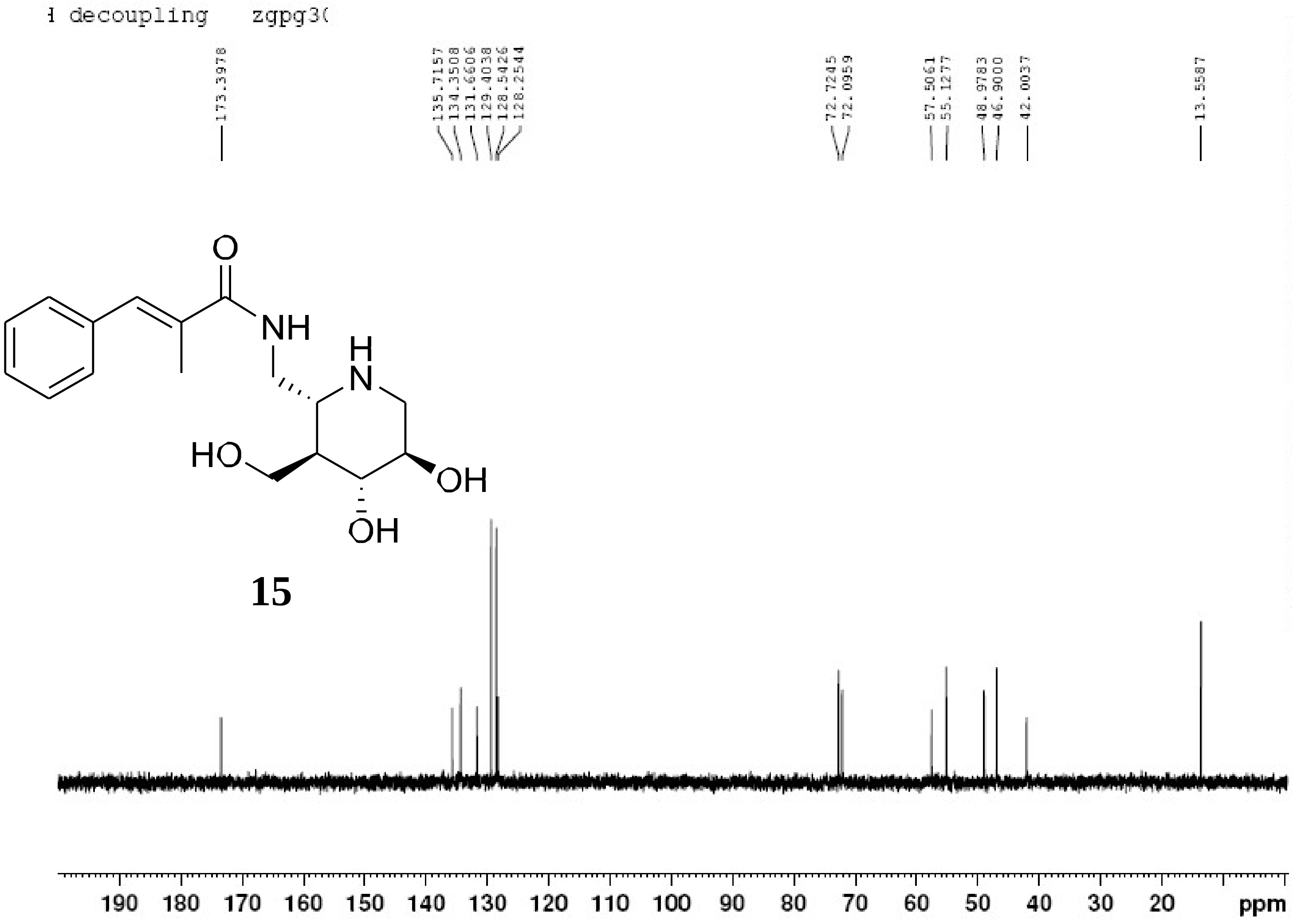

^{13}C spectrum of compound **15 (**150 MHz, D_2O)

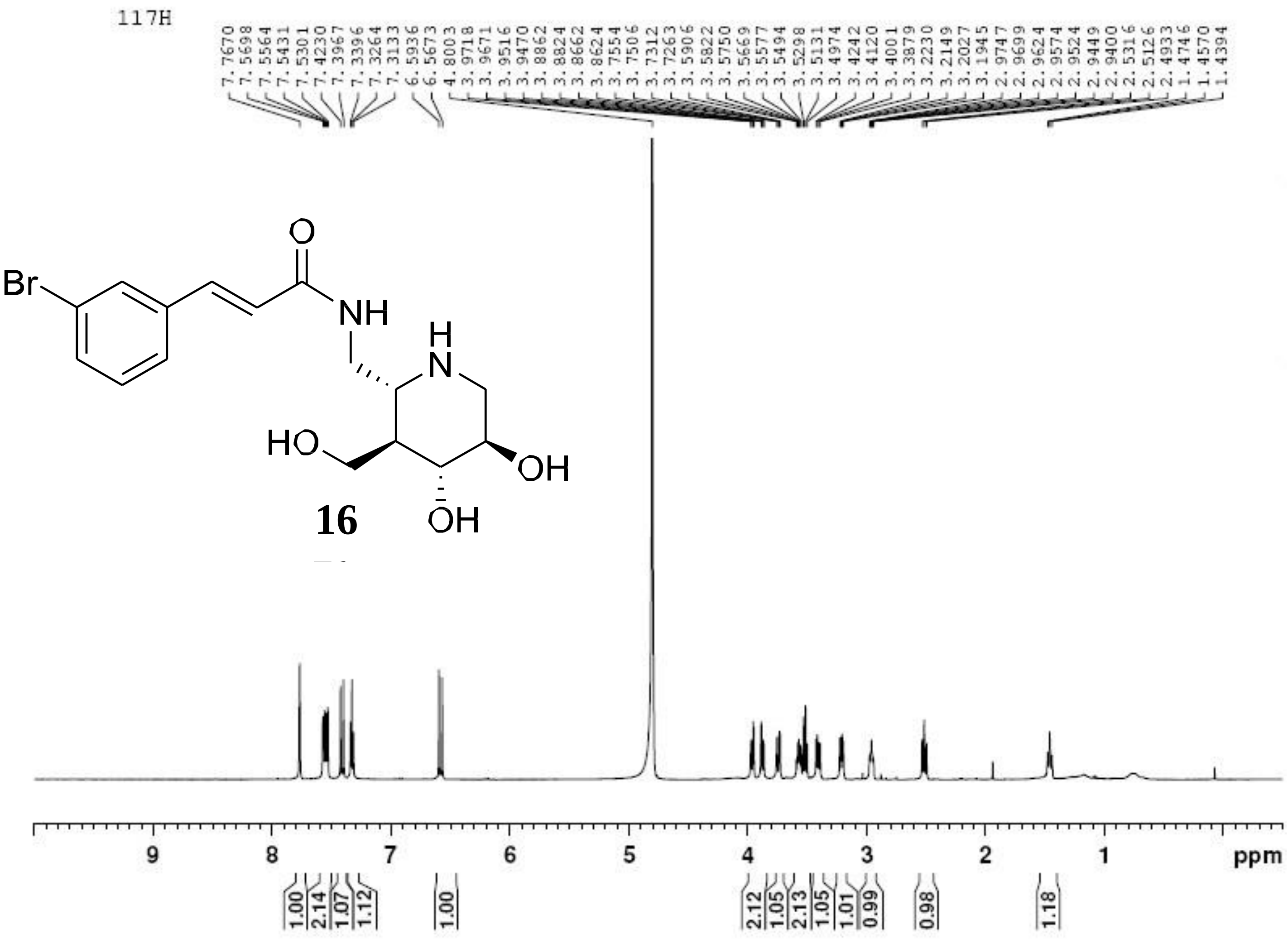

^{1}H spectrum of compound **16** (600 MHz, D_2O)

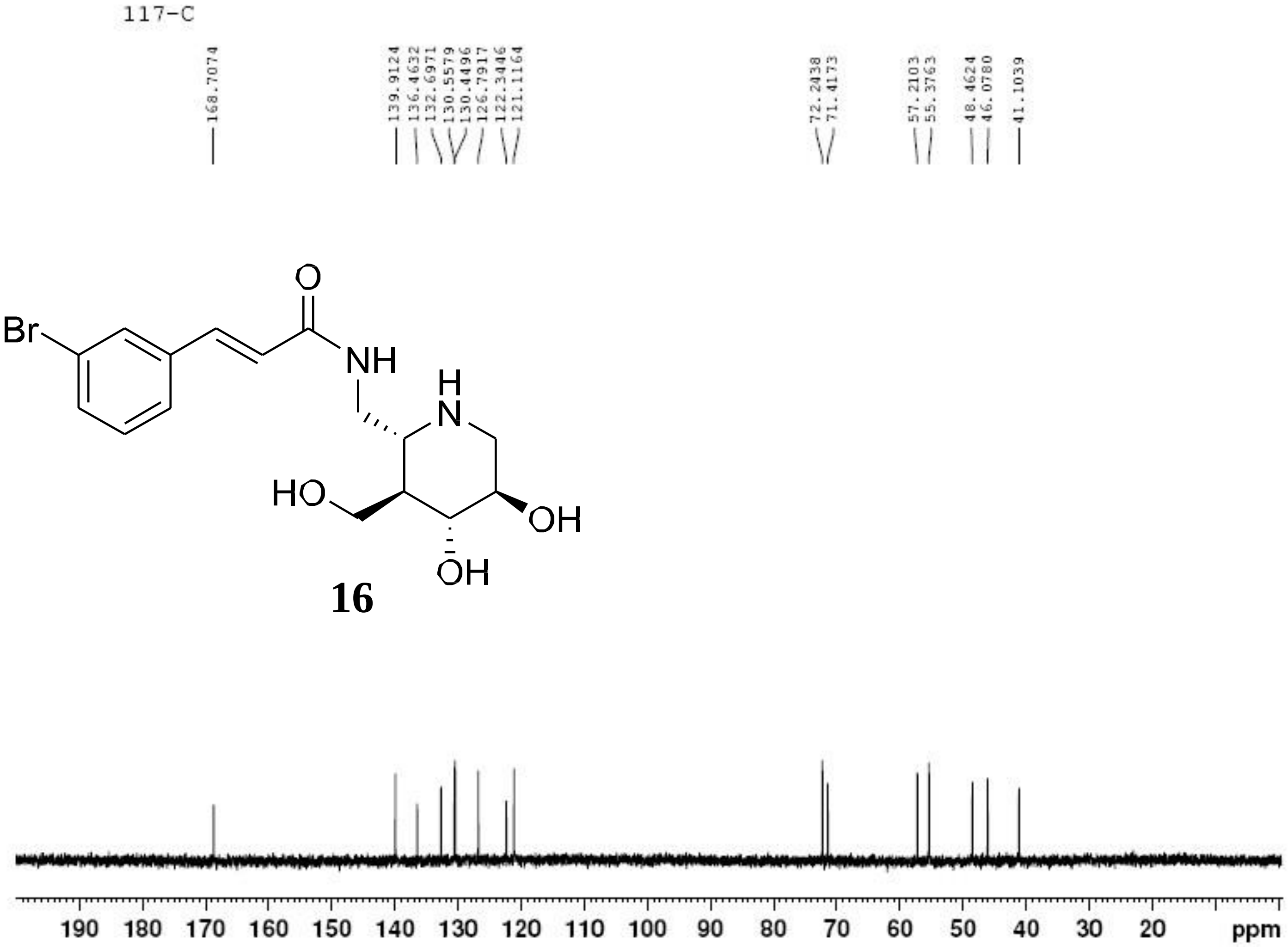

16

^{13}C spectrum of compound **16 (**150 MHz, D_2O)

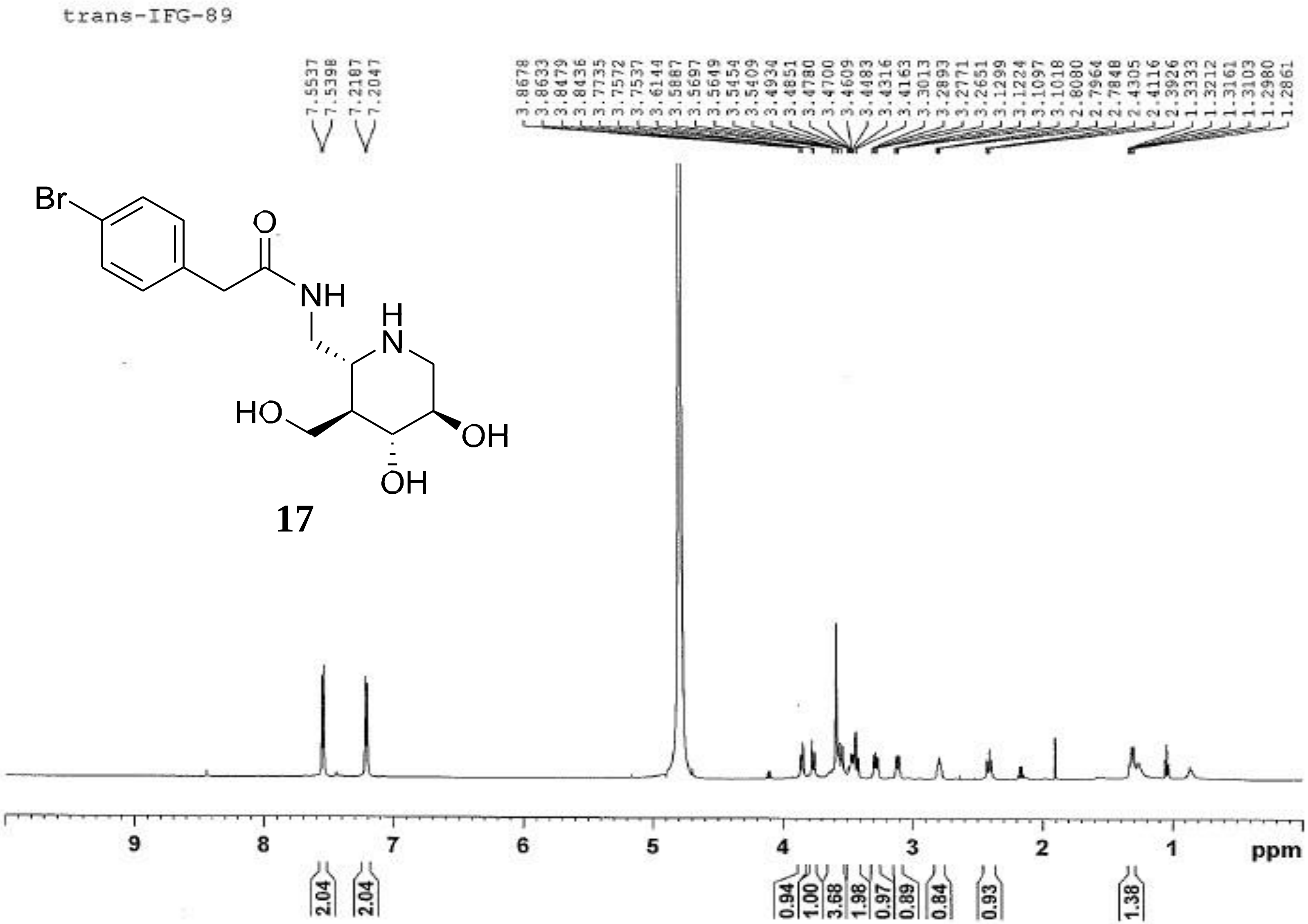

^{1}H spectrum of compound **17 (**600 MHz, D_2O)

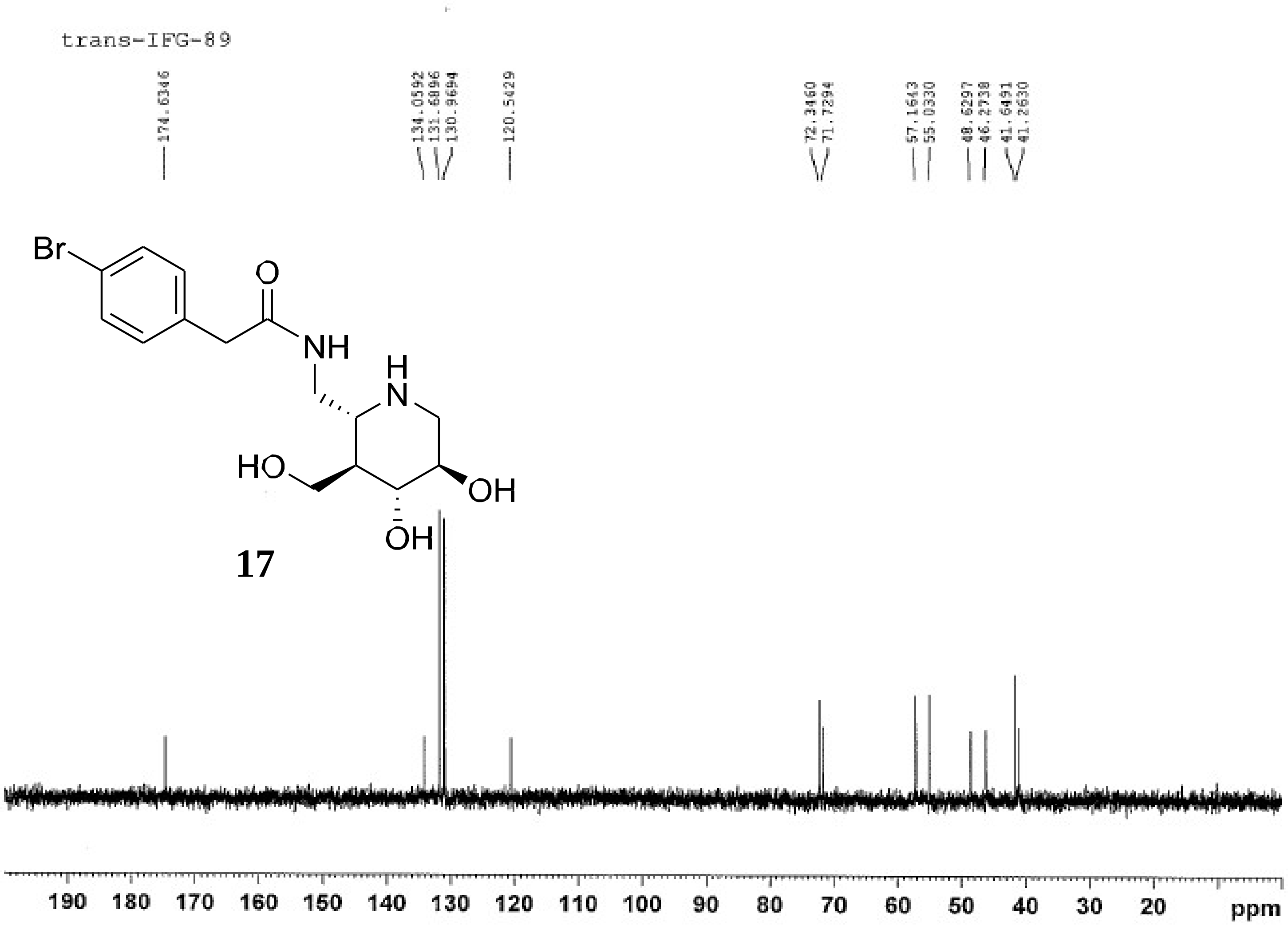

^{13}C spectrum of compound **17** **(**150 MHz, D_2O)

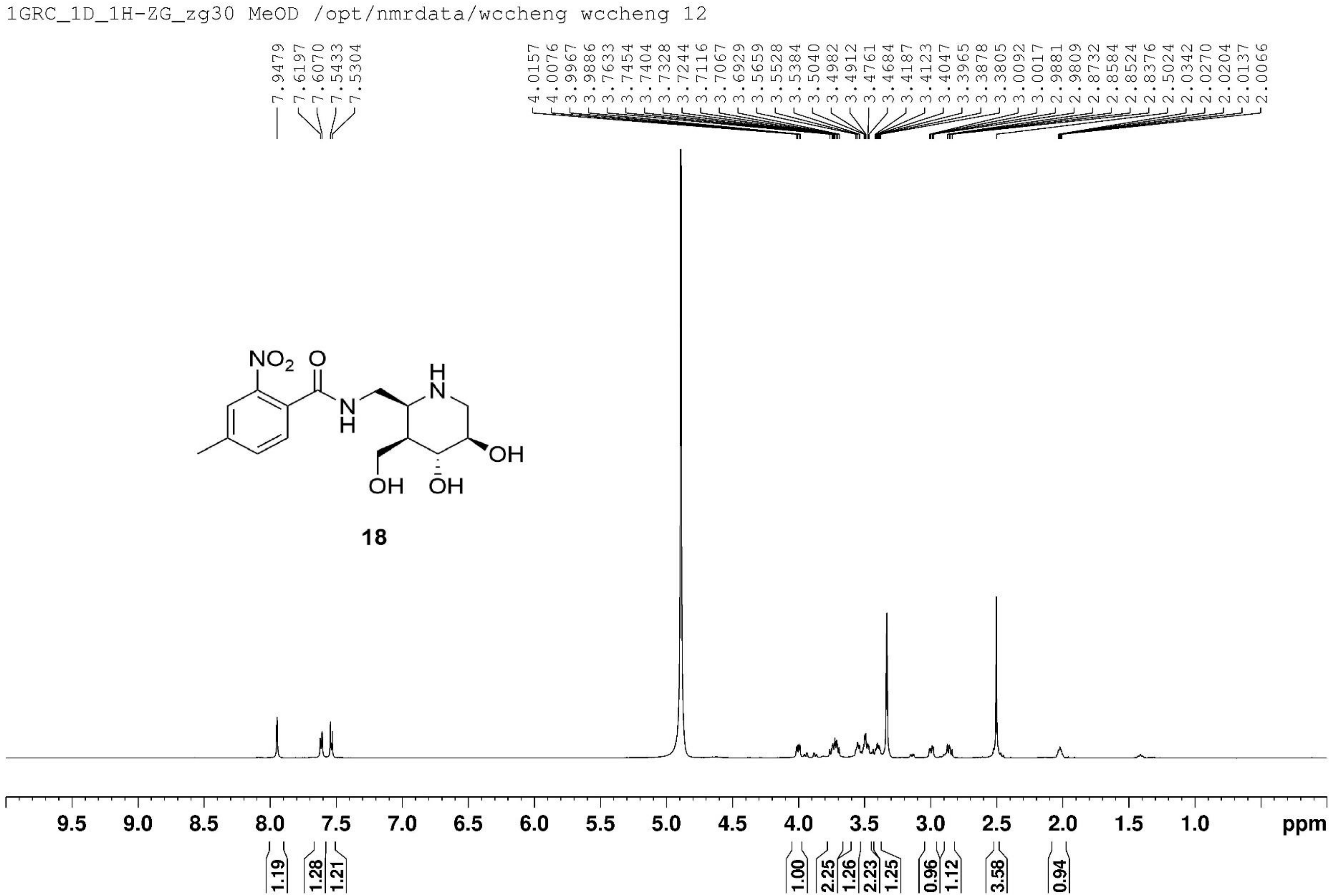

^{1}H spectrum of compound **18** (600 MHz, MeOD)

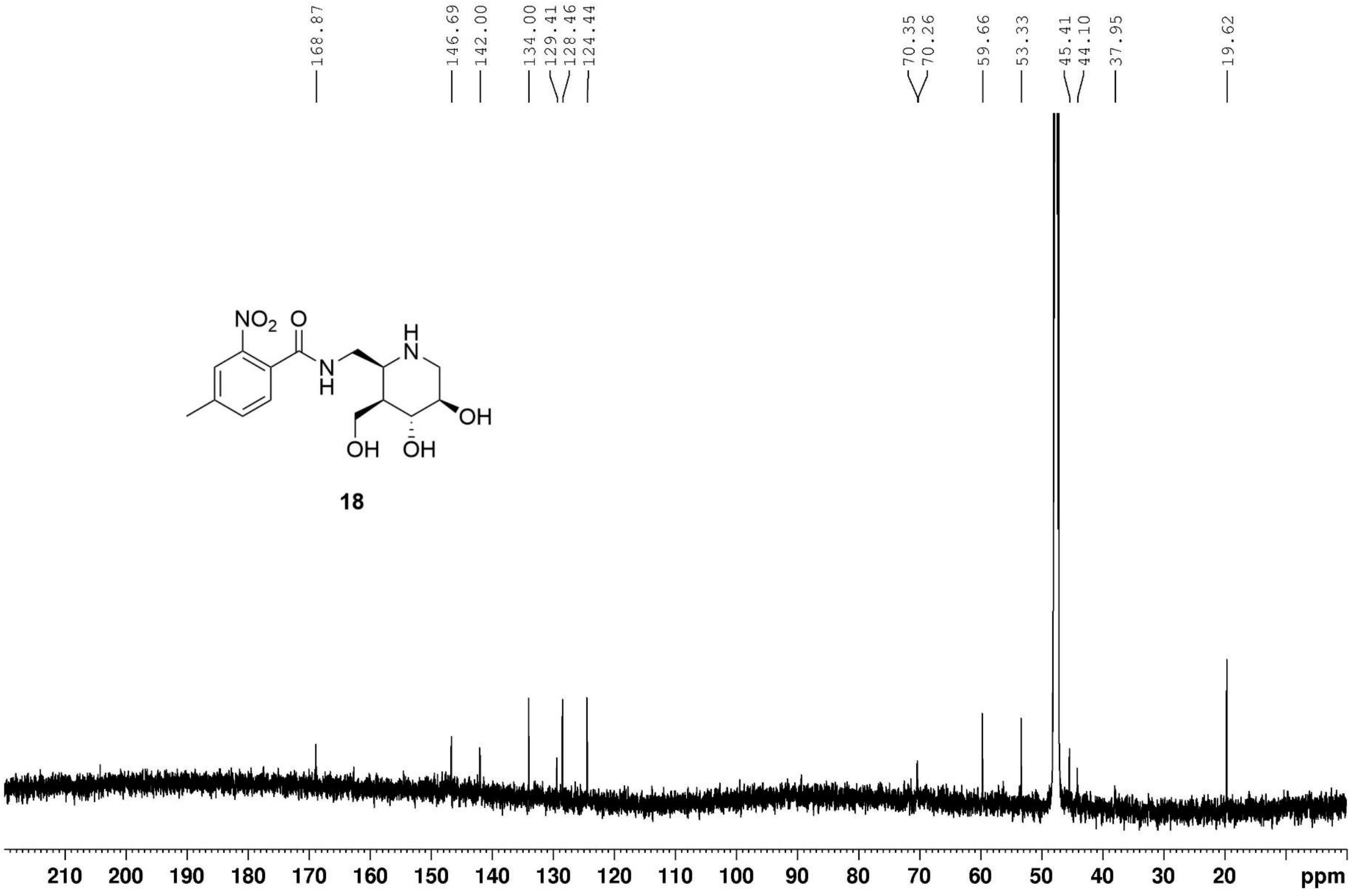

^{13}C spectrum of compound **18** (150 MHz, MeOD)

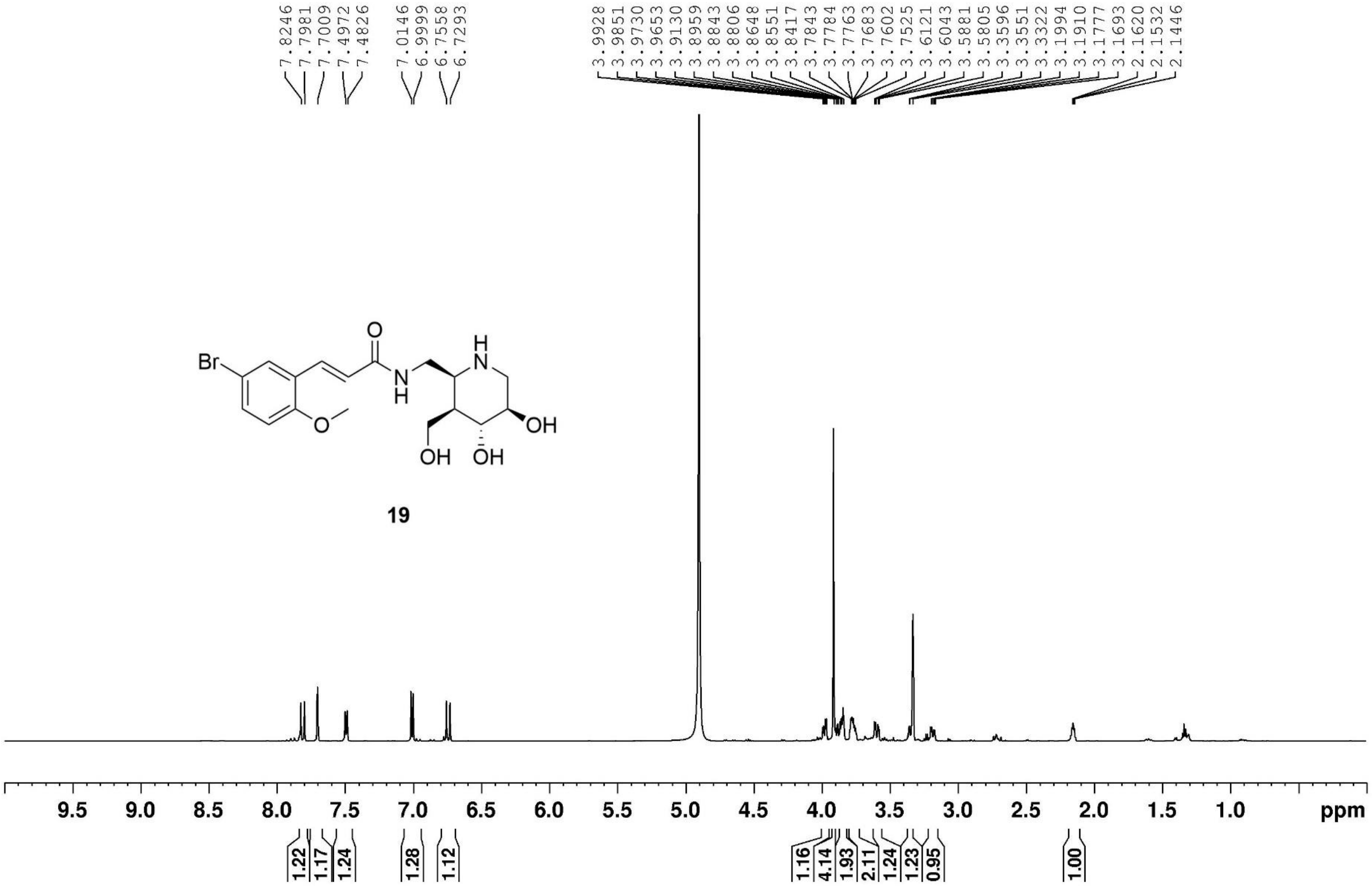
7.8246
7.7981
7.7009
7.4972
7.4826
7.0146
6.9999
6.7558
6.7293
3.9928
3.9851
3.9730
3.9653
3.9130
3.8959
3.8843
3.8806
3.8648
3.8551
3.8417
3.7843
3.7784
3.7763
3.7683
3.7602
3.7525
3.6121
3.6043
3.5881
3.5805
3.3596
3.3551
3.3322
3.1994
3.1910
3.1777
3.1693
2.1620
2.1532
2.1446
Br
O
N
H
H
N
O
OH
OH
OH
19
9.5
9.0
8.5
8.0
7.5
7.0
6.5
6.0
5.5
5.0
4.5
4.0
3.5
3.0
2.5
2.0
1.5
1.0
ppm
1.22
1.17
1.24
1.28
1.12
1.16
4.14
1.93
2.11
1.24
1.23
0.95
1.00

^{1}H spectrum of compound **19** (600 MHz, MeOD)

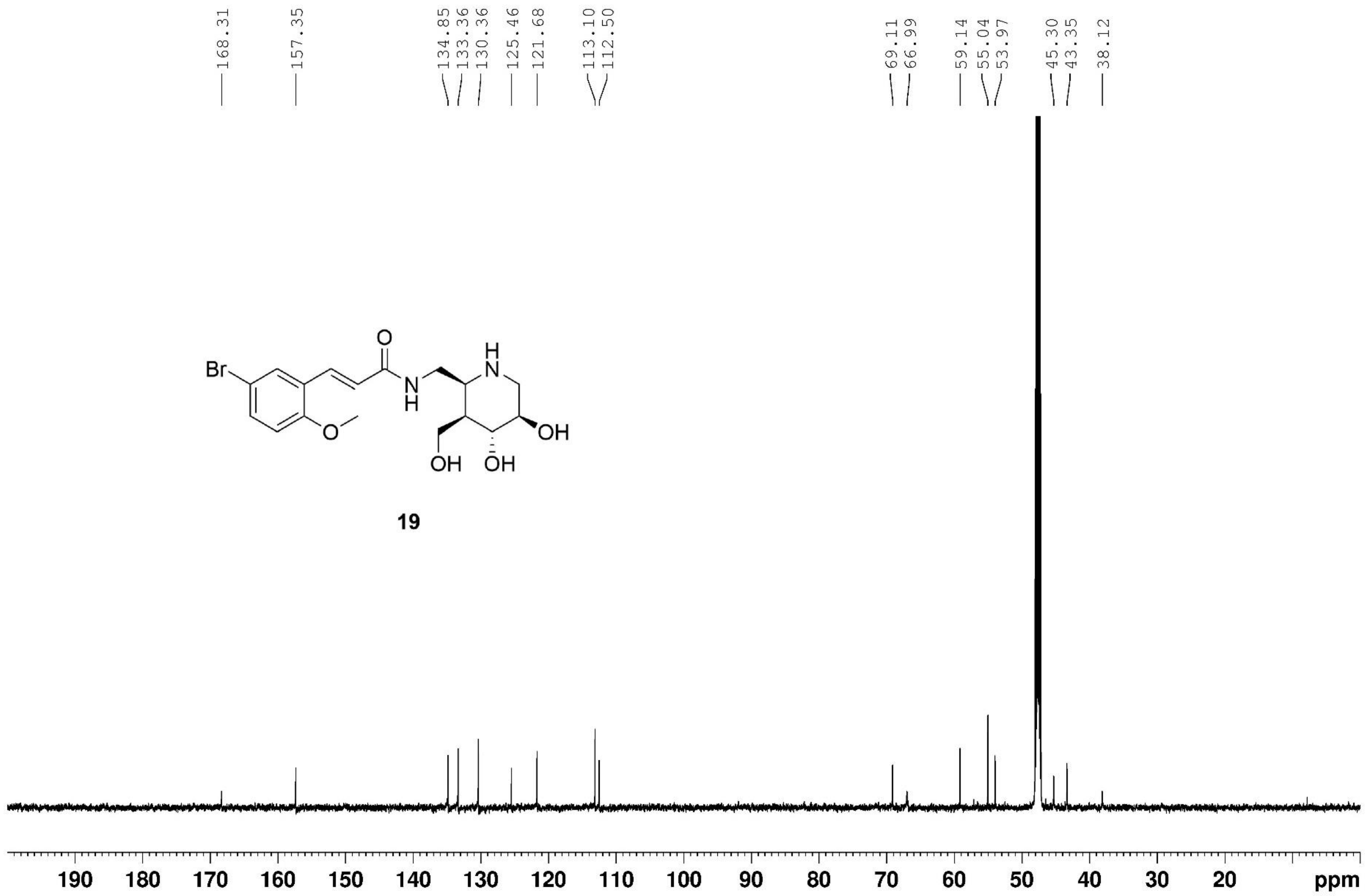

^{13}C spectrum of compound **19** (150 MHz, MeOD)

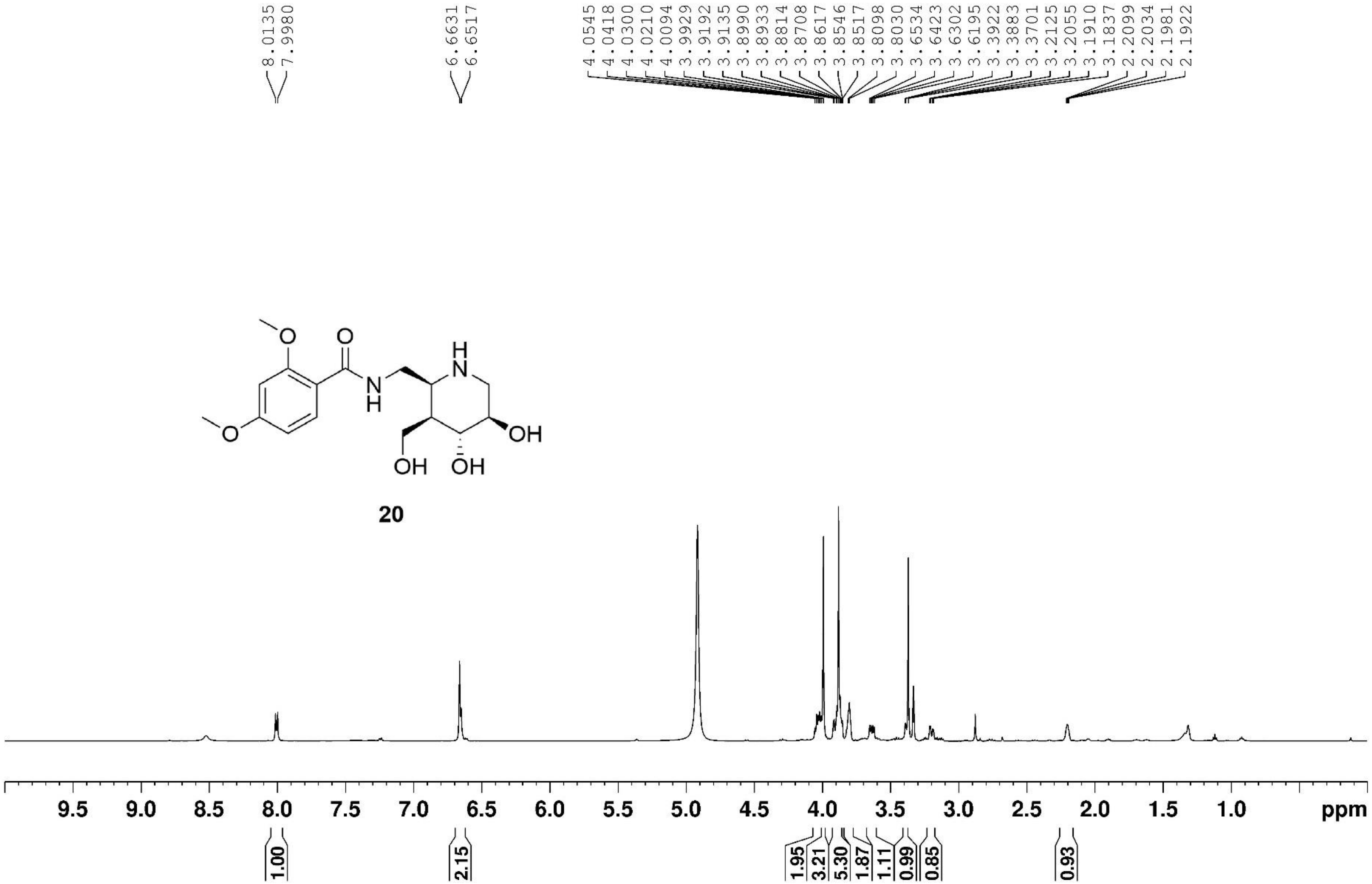

20

^{1}H spectrum of compound **20** (600 MHz, MeOD)

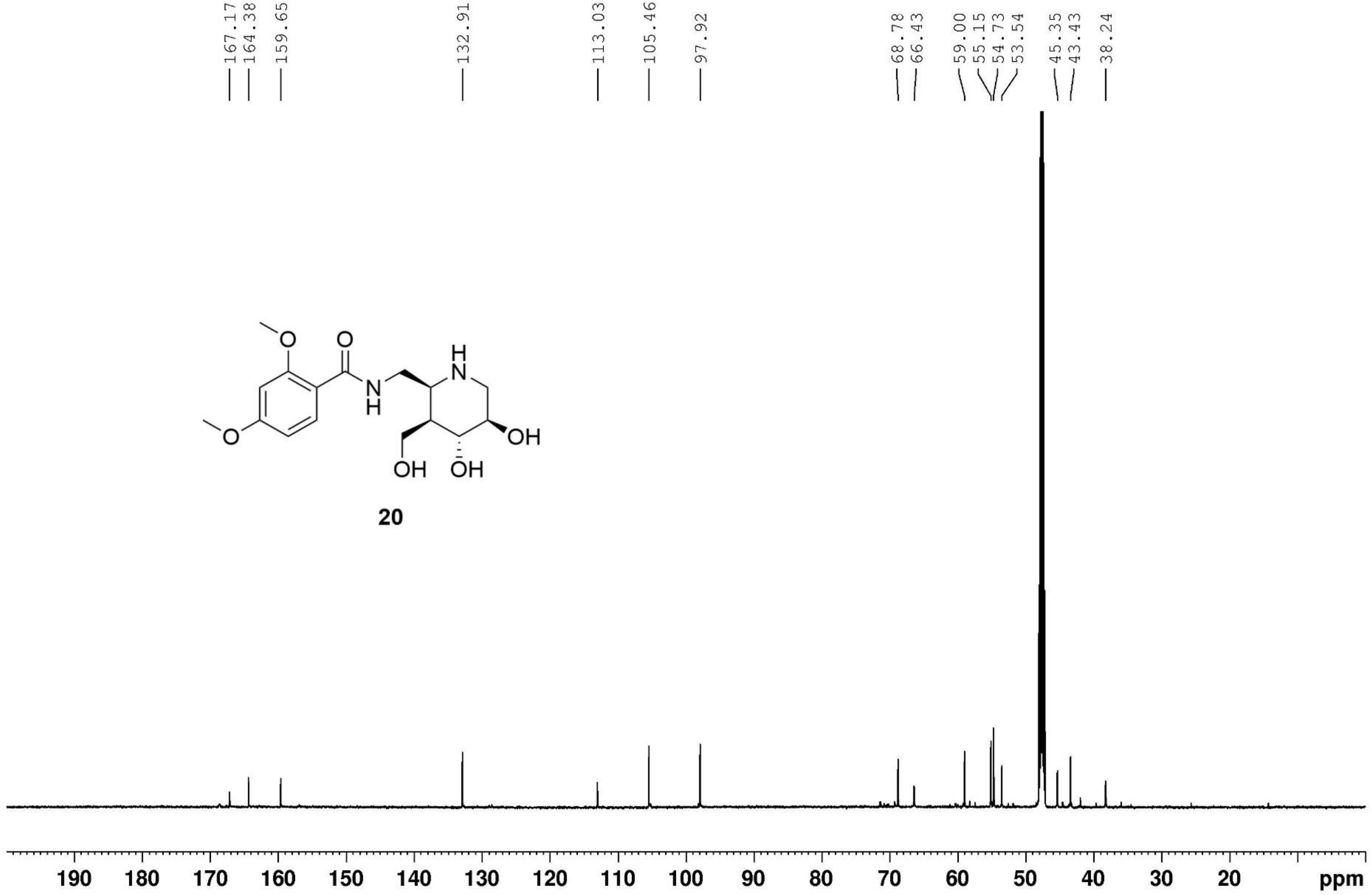

^{13}C spectrum of compound **20** (150 MHz, MeOD)

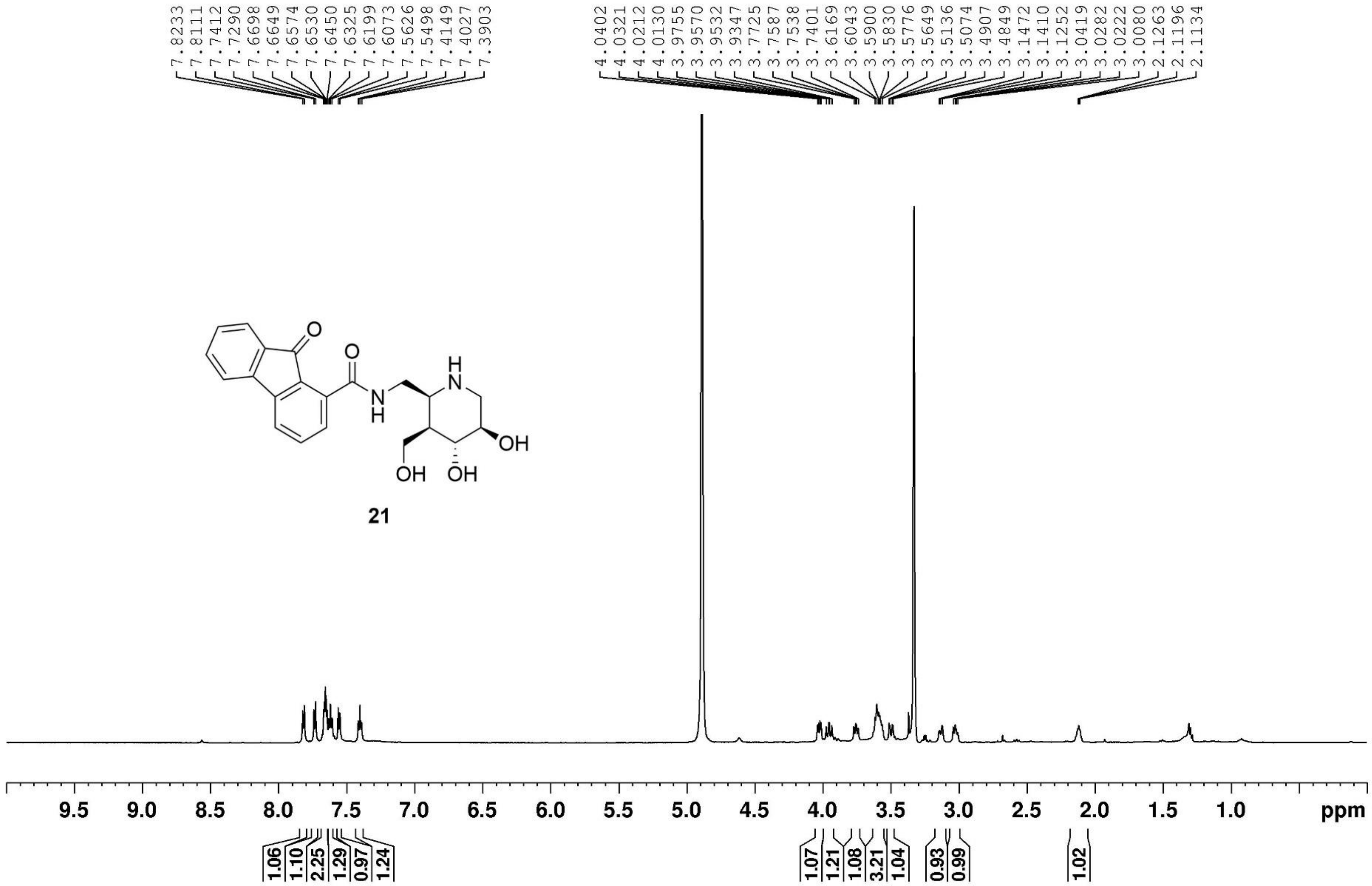

^{1}H spectrum of compound **21** (600 MHz, MeOD)

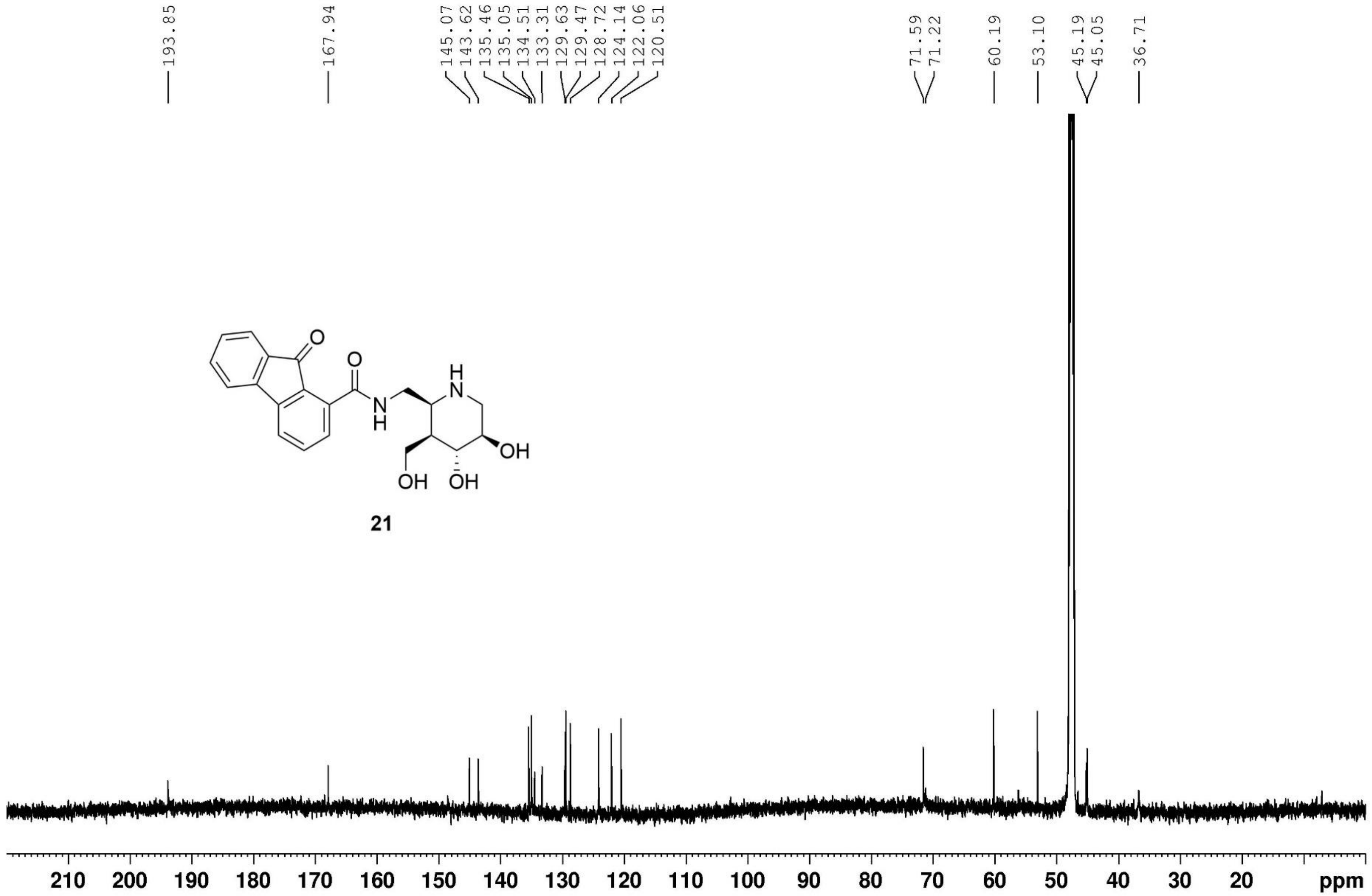

^{13}C spectrum of compound **21** (150 MHz, MeOD)

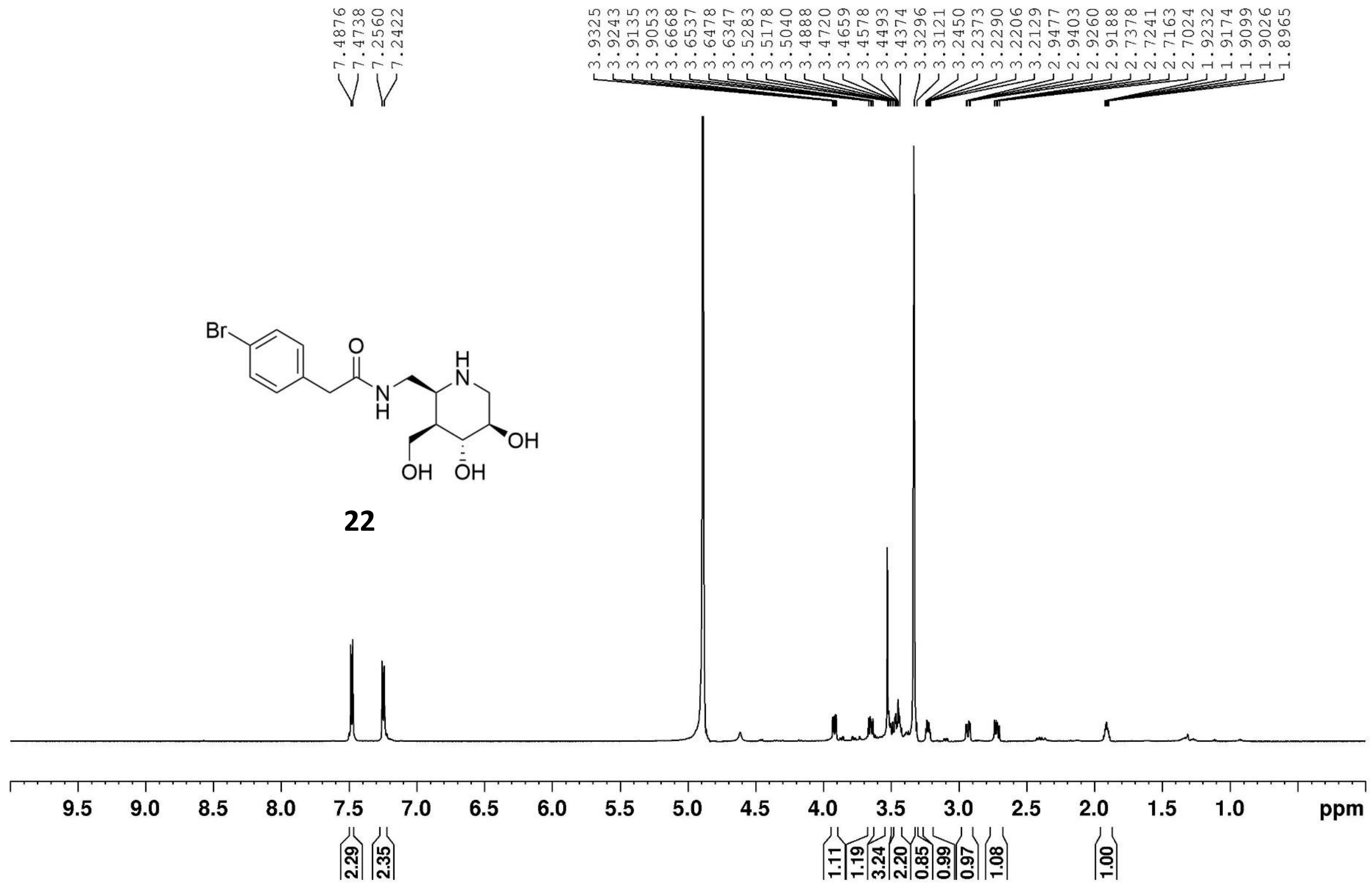

^{1}H spectrum of compound **22** (600 MHz, MeOD)

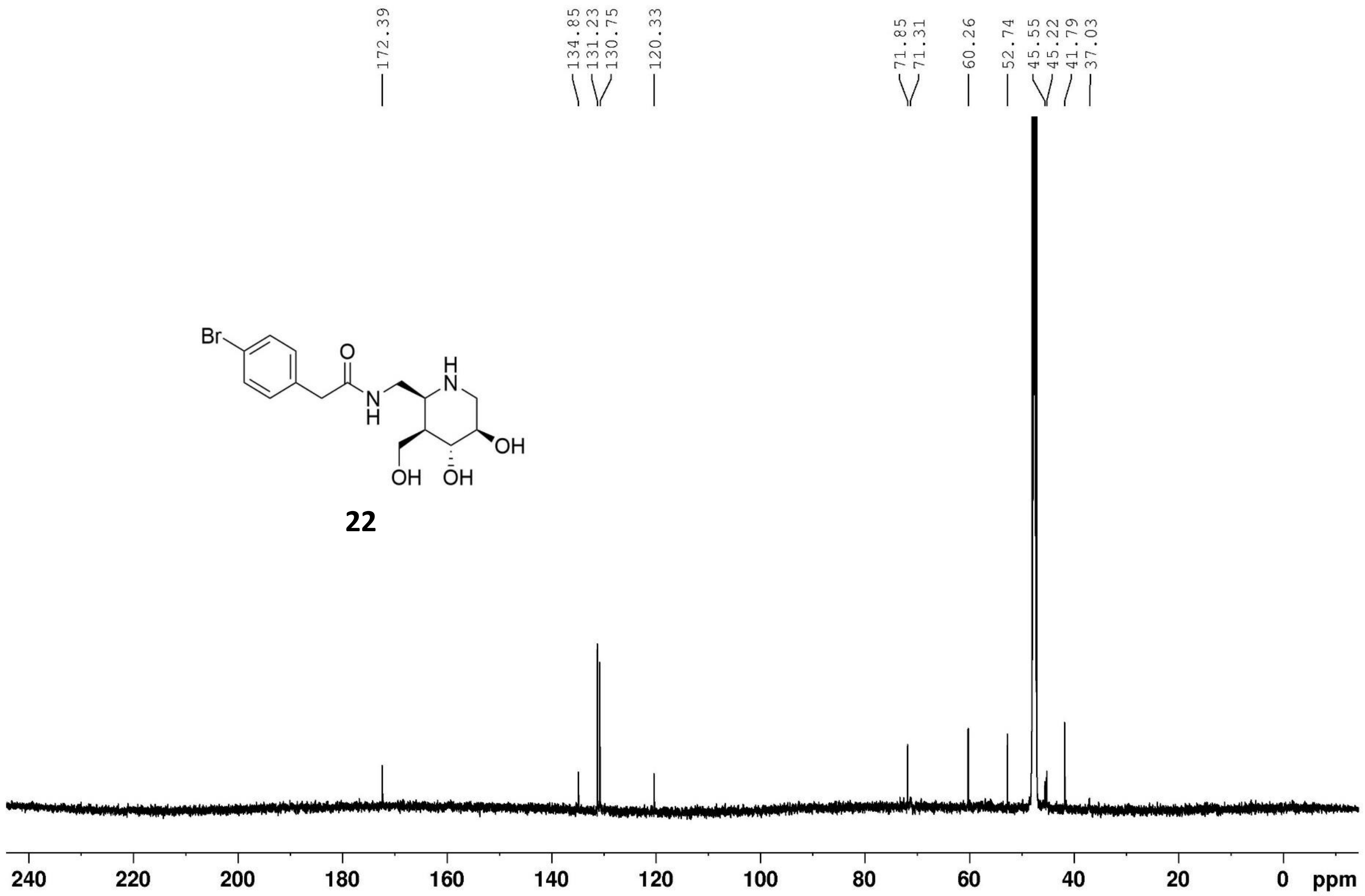

^{13}C spectrum of compound **22** (150 MHz, MeOD)

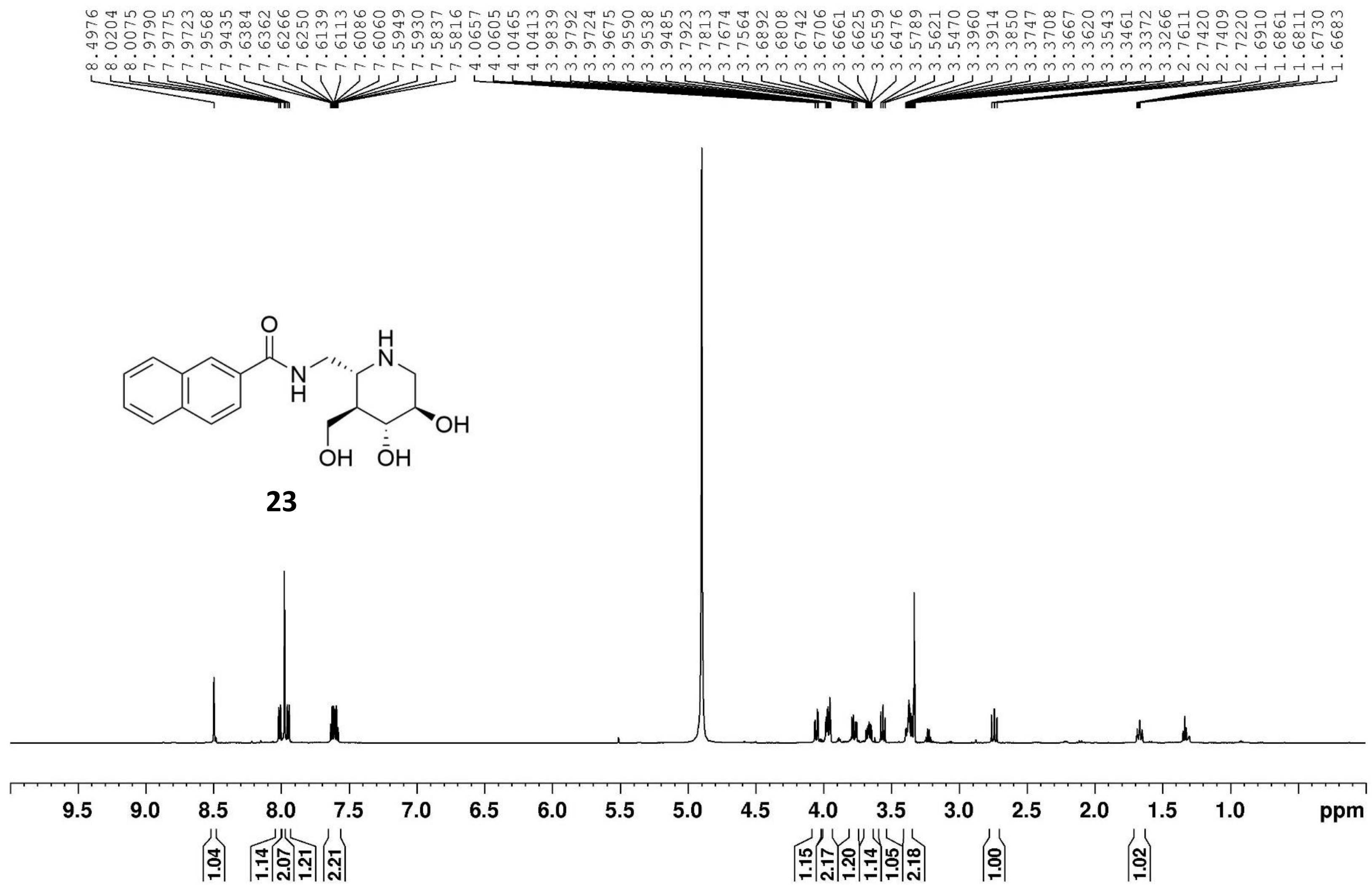

^{1}H spectrum of compound **23** (600 MHz, MeOD)

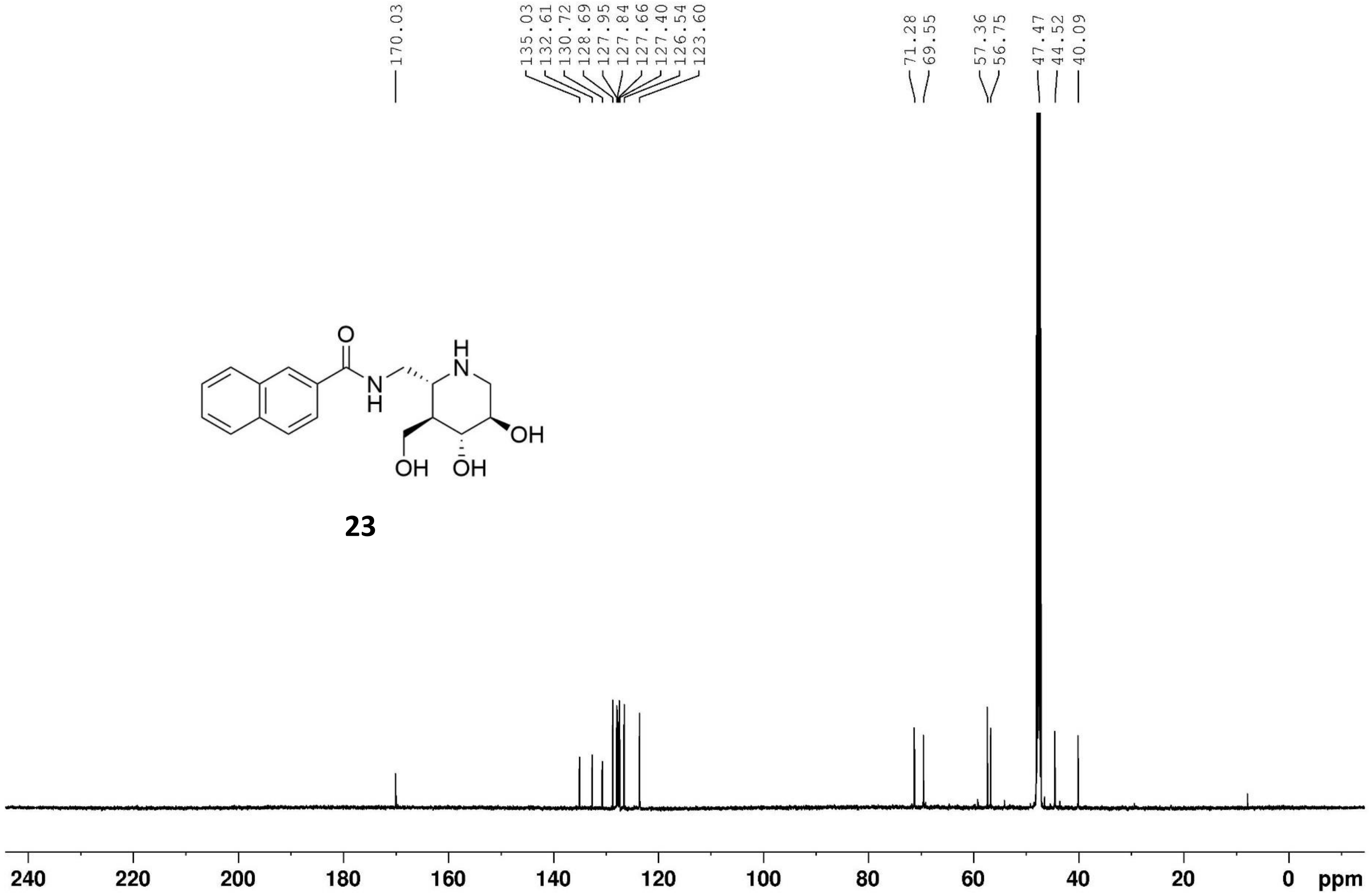

^{13}C spectrum of compound **23** (150 MHz, MeOD)

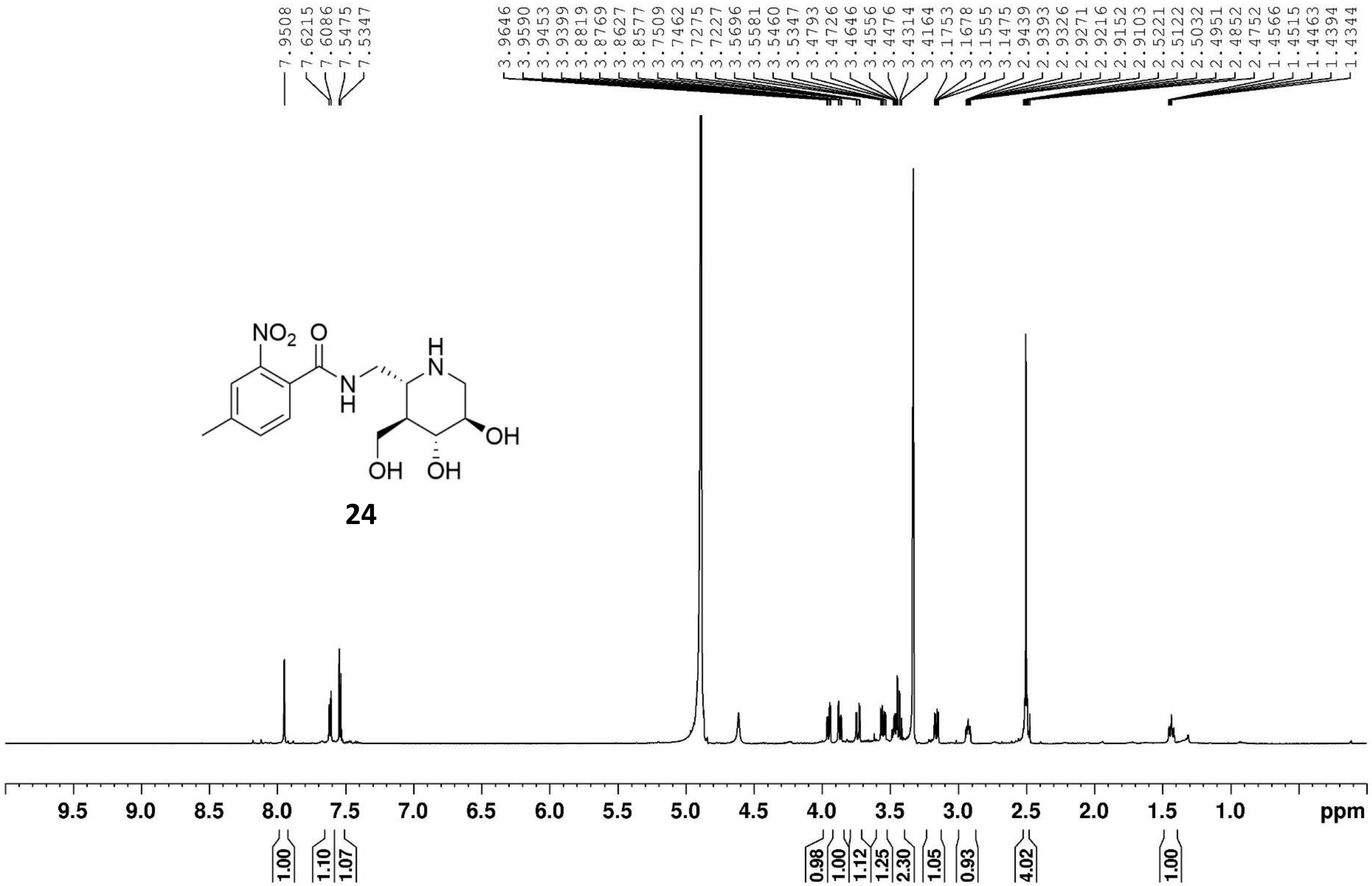

^{1}H spectrum of compound **24** (600 MHz, MeOD)

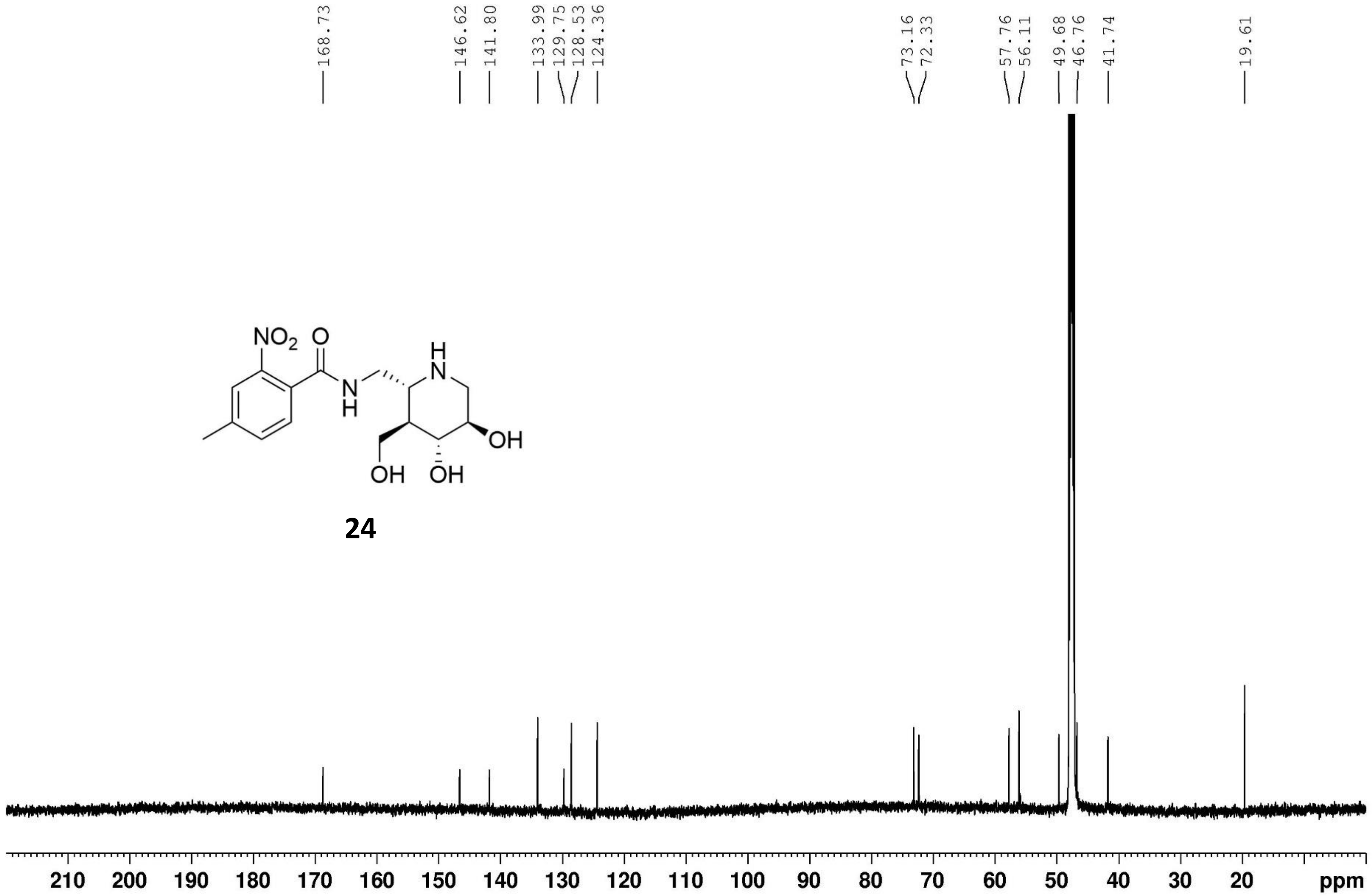

^{13}C spectrum of compound **24** (150 MHz, MeOD)

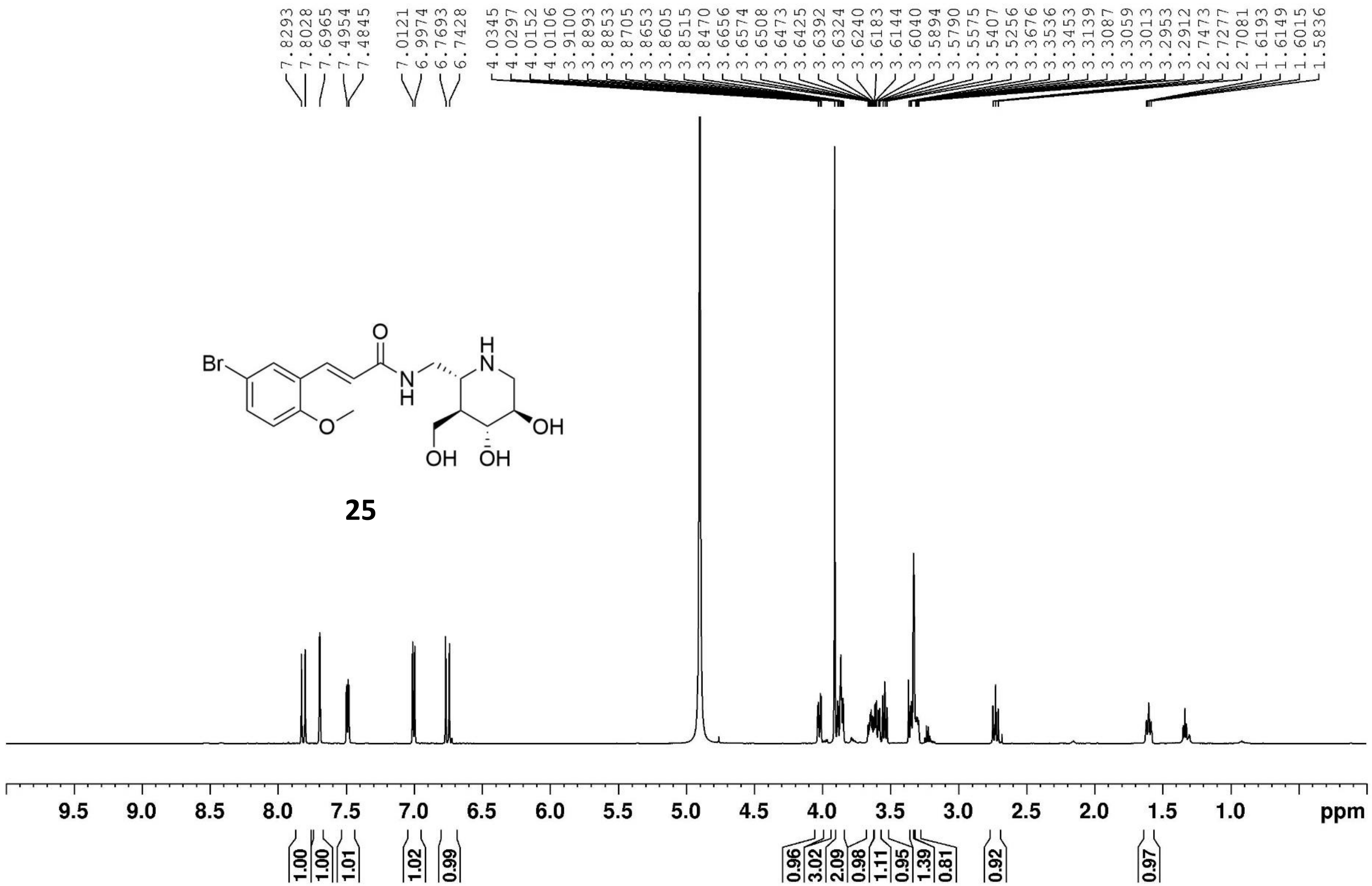

^{1}H spectrum of compound **25** (600 MHz, MeOD)

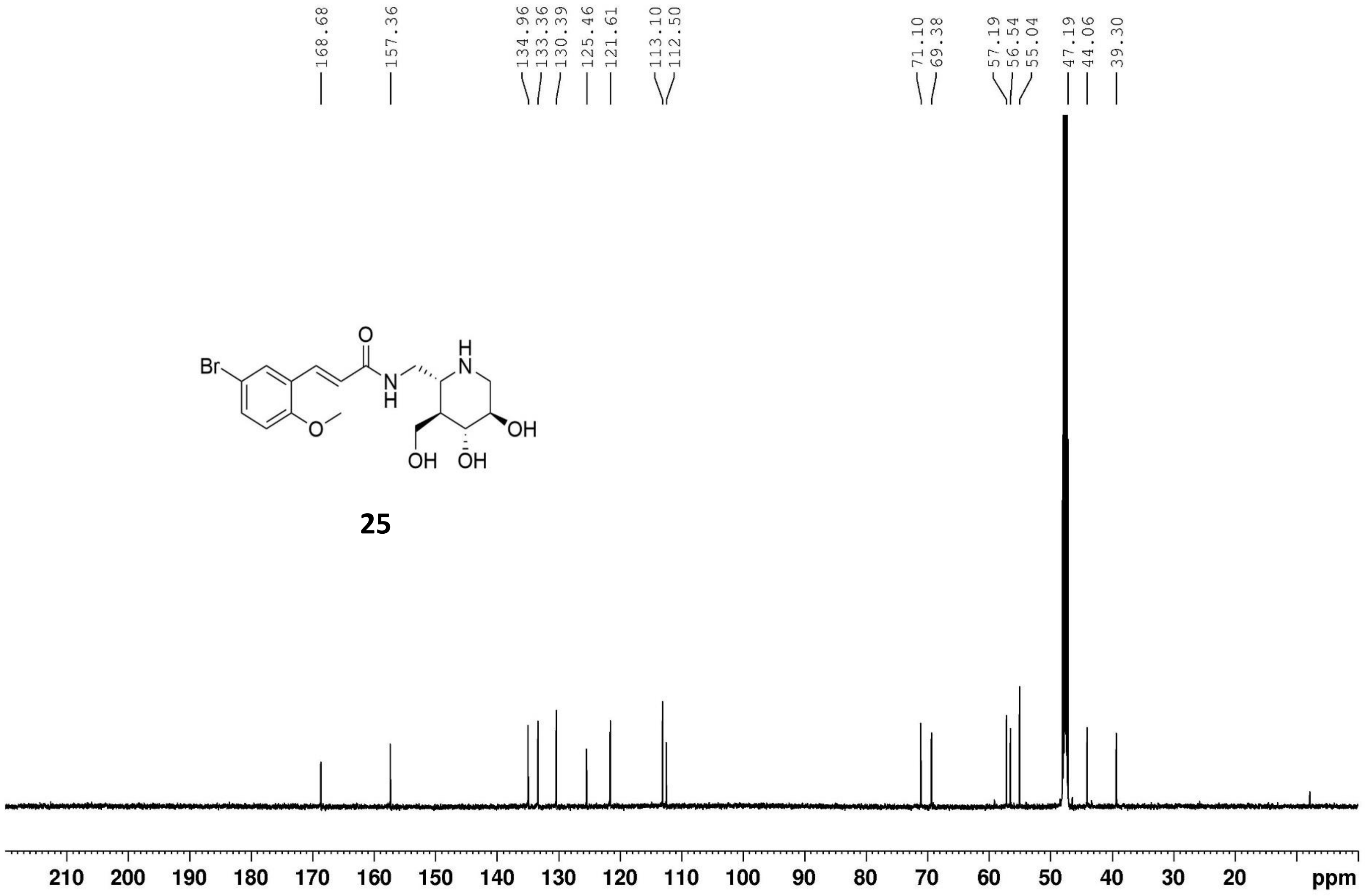

^{13}C spectrum of compound **25** (150 MHz, MeOD)

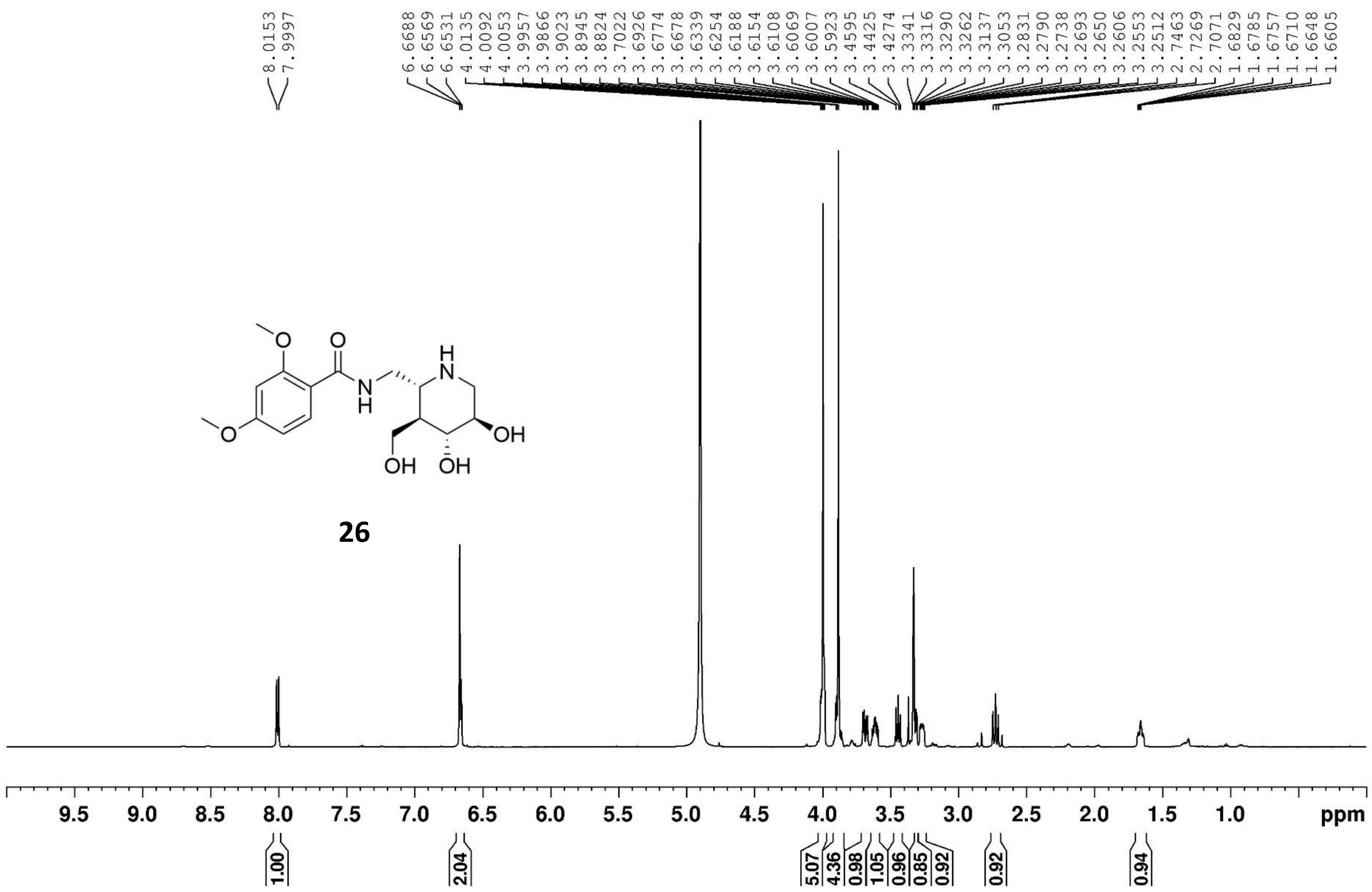

^{1}H spectrum of compound **26** (600 MHz, MeOD)

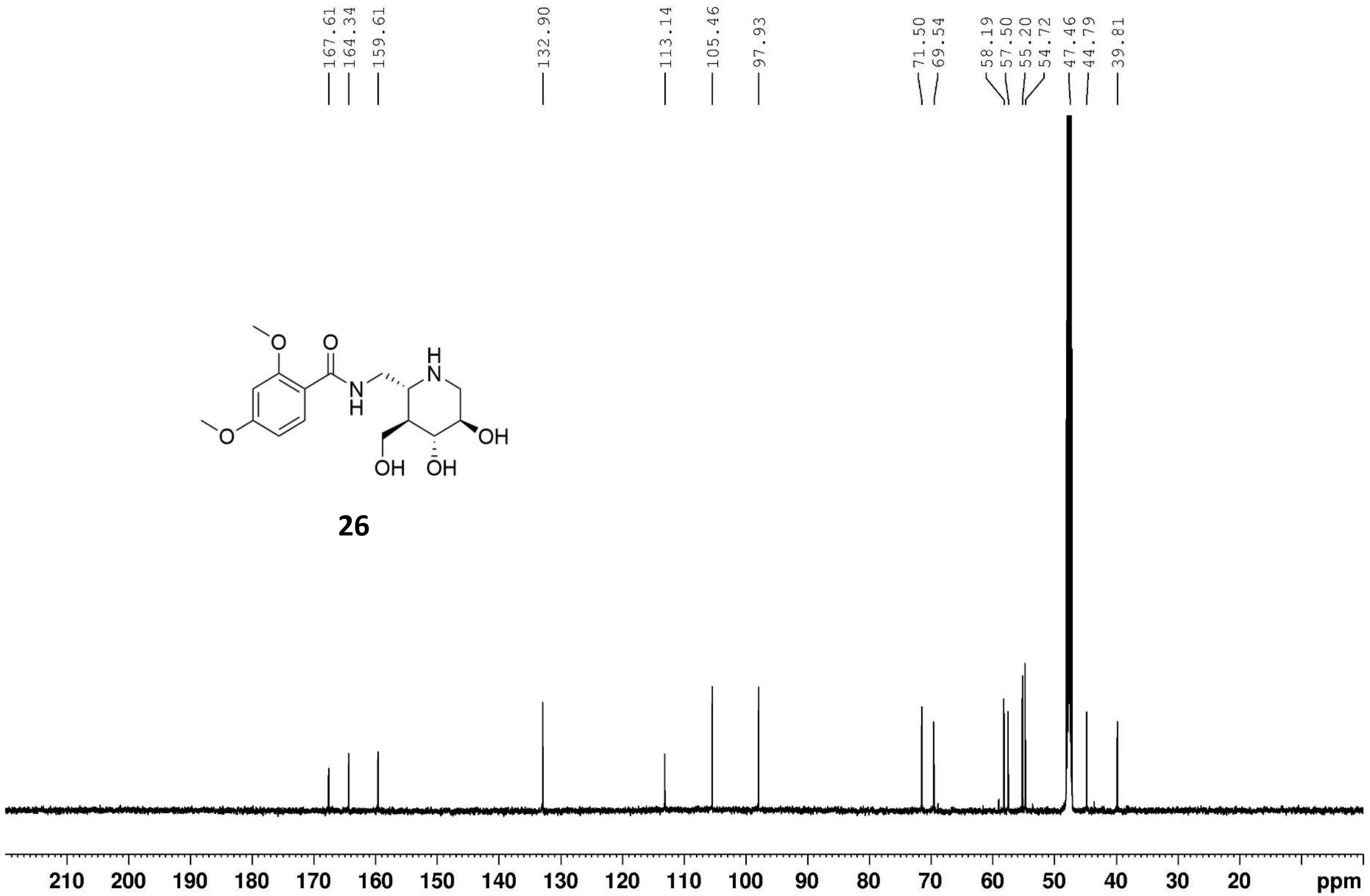

^{13}C spectrum of compound **26** (150 MHz, MeOD)

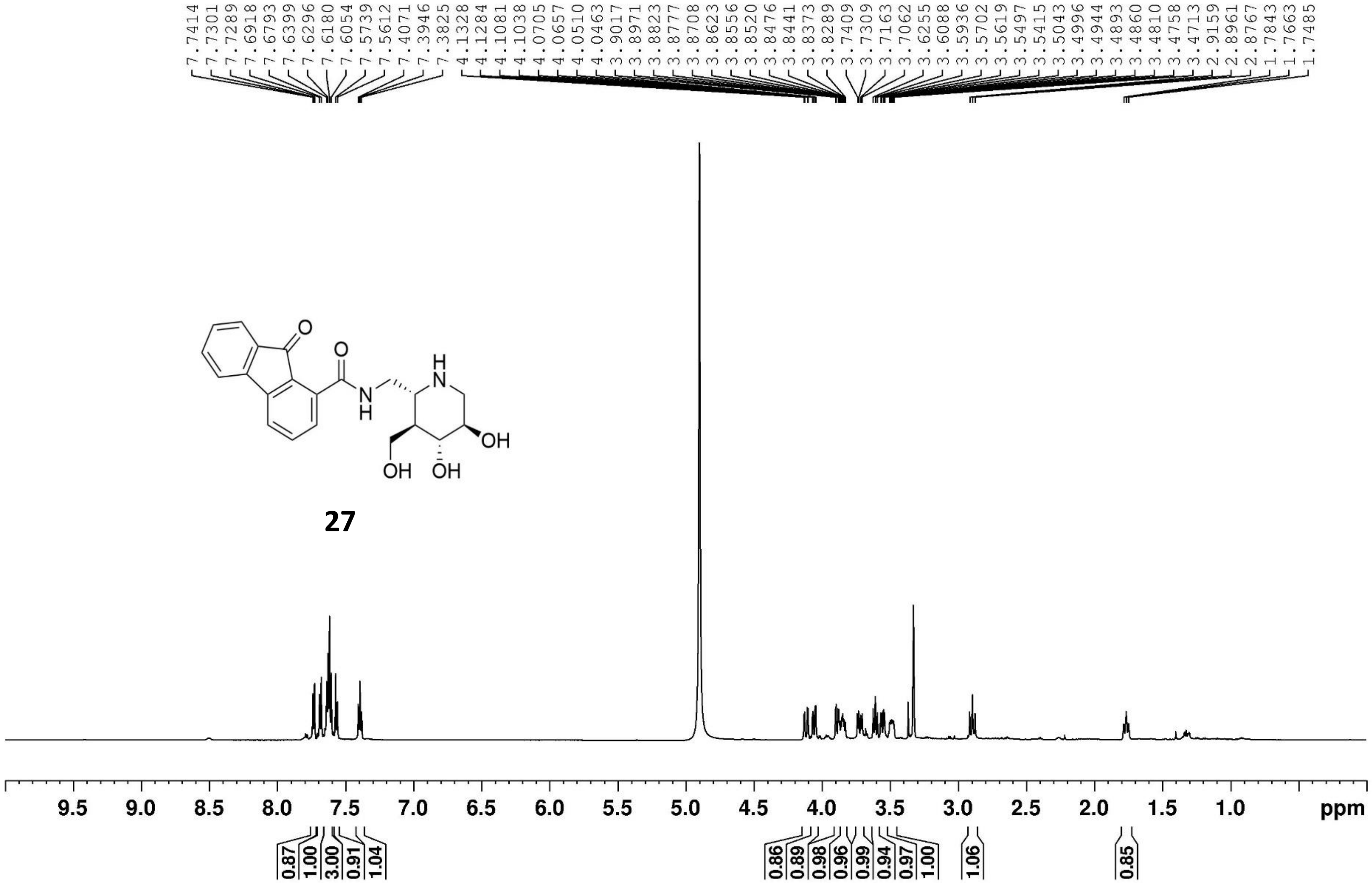

^{1}H spectrum of compound **27** (600 MHz, MeOD)

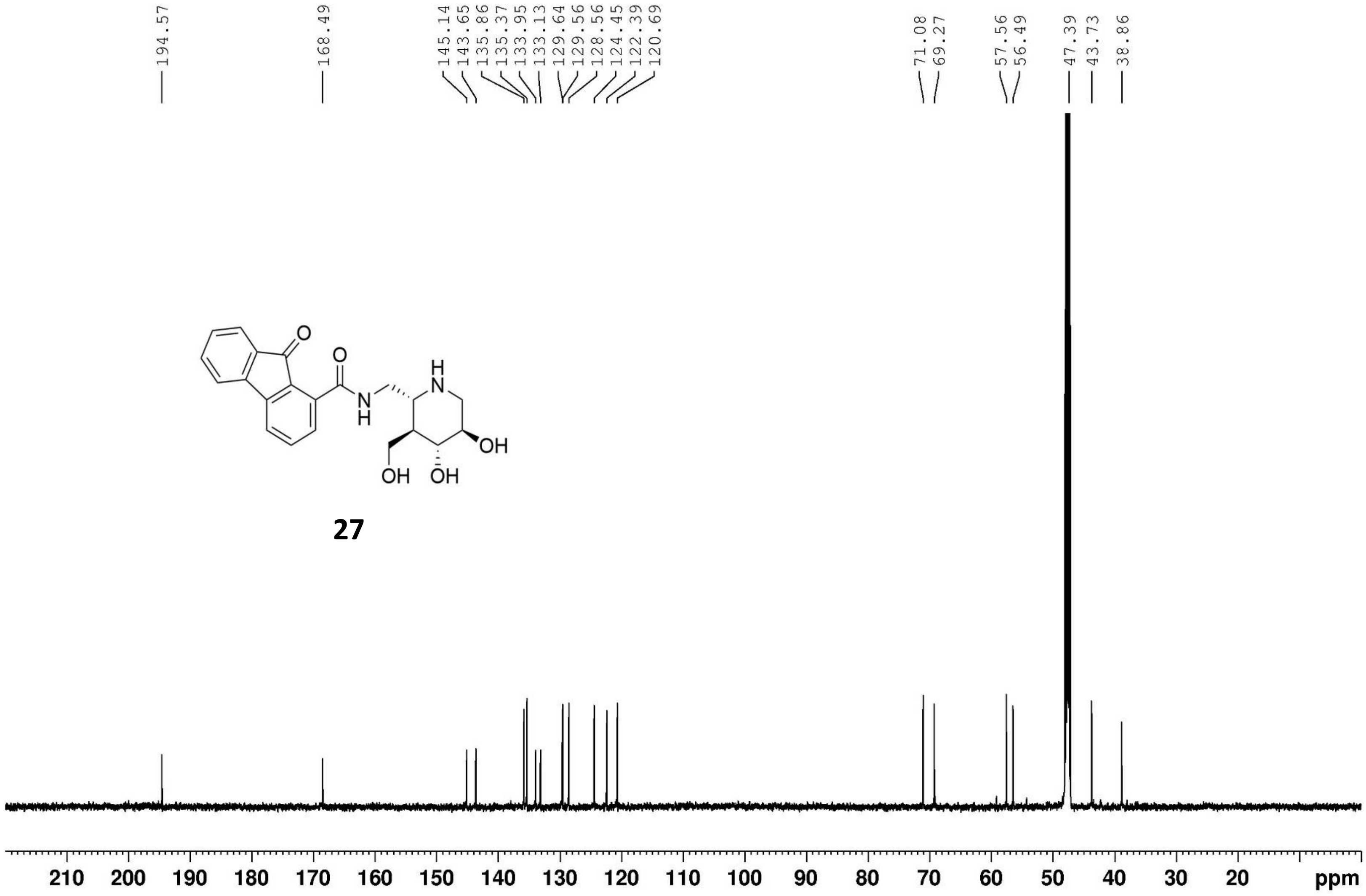

^{13}C spectrum of compound **27** (150 MHz, MeOD)
