# Supplementary material for: A practical synthesis of nitrone-derived C5a-functionalized isofagomines as protein stabilizers to treat Gaucher disease
Source: Commun Chem. 2024 Apr 20;7:91. doi: 10.1038/s42004-024-01164-9 (PMC11032326; doi:10.1038/s42004-024-01164-9)
Supplement: Supplementary file 4 — reporting summary [file 42004_2024_1164_MOESM4_ESM.pdf]

nature portfolio

Corresponding author(s): Wei-Chieh Cheng
Last updated by author(s): Mar 18, 2024

Reporting Summary

Nature Portfolio wishes to improve the reproducibility of the work that we publish. This form provides structure for consistency and transparency in reporting. For further information on Nature Portfolio policies, see our Editorial Policies and the Editorial Policy Checklist.

Statistics

For all statistical analyses, confirm that the following items are present in the figure legend, table legend, main text, or Methods section.

n/a	Confirmed	
☐	☒	The exact sample size (*n*) for each experimental group/condition, given as a discrete number and unit of measurement
☐	☒	A statement on whether measurements were taken from distinct samples or whether the same sample was measured repeatedly
☐	☒	The statistical test(s) used AND whether they are one- or two-sided<br>*Only common tests should be described solely by name; describe more complex techniques in the Methods section.*
☒	☐	A description of all covariates tested
☒	☐	A description of any assumptions or corrections, such as tests of normality and adjustment for multiple comparisons
☐	☒	A full description of the statistical parameters including central tendency (e.g. means) or other basic estimates (e.g. regression coefficient) AND variation (e.g. standard deviation) or associated estimates of uncertainty (e.g. confidence intervals)
☒	☐	For null hypothesis testing, the test statistic (e.g. *F*, *t*, *r*) with confidence intervals, effect sizes, degrees of freedom and *P* value noted<br>*Give P values as exact values whenever suitable.*
☒	☐	For Bayesian analysis, information on the choice of priors and Markov chain Monte Carlo settings
☒	☐	For hierarchical and complex designs, identification of the appropriate level for tests and full reporting of outcomes
☒	☐	Estimates of effect sizes (e.g. Cohen's *d*, Pearson's *r*), indicating how they were calculated

Our web collection on statistics for biologists contains articles on many of the points above.

Software and code

Policy information about availability of computer code

Data collection	Molecular Devices SpectraMax M5 Microplate Reader (enzyme activity and cell-based assays), Thermo Fisher Scientific QuantStudio® 5 Real-Time PCR System (Thermal stability shift assay)
Data analysis	GraphPad Prism 6.0 (enzyme activity and cell-based assays), Microsoft Excel (Thermal stability shift assay)

For manuscripts utilizing custom algorithms or software that are central to the research but not yet described in published literature, software must be made available to editors and reviewers. We strongly encourage code deposition in a community repository (e.g. GitHub). See the Nature Portfolio guidelines for submitting code & software for further information.

Data

Policy information about availability of data

All manuscripts must include a data availability statement. This statement should provide the following information, where applicable:

- Accession codes, unique identifiers, or web links for publicly available datasets
- A description of any restrictions on data availability
- For clinical datasets or third party data, please ensure that the statement adheres to our policy

Detailed experimental procedures and characterizations of new compounds are available in Supplementary Information. 1H and 13C NMR spectra can be found in the Supplementary Data 1. All other data supporting the key findings of this study are available within the article and its Supplementary information files.

Human research participants

Policy information about studies involving human research participants and Sex and Gender in Research.

Reporting on sex and gender	This study did not involve any human research participants
Population characteristics	This study did not involve any human research participants
Recruitment	This study did not involve any human research participants
Ethics oversight	This study did not involve any human research participants

Note that full information on the approval of the study protocol must also be provided in the manuscript.

Field-specific reporting

Please select the one below that is the best fit for your research. If you are not sure, read the appropriate sections before making your selection.

☒ Life sciences ☐ Behavioural & social sciences ☐ Ecological, evolutionary & environmental sciences

For a reference copy of the document with all sections, see nature.com/documents/nr-reporting-summary-flat.pdf

Life sciences study design

All studies must disclose on these points even when the disclosure is negative.

Sample size	For enzyme-based and cell-based assay, samples for each condition have 3 technical replicates and 3 independent biological repeats.
Data exclusions	No data was excluded.
Replication	The number of biological repeats were noted in the figure legends and the method section.
Randomization	Cells from the same dish were collected and the same number of cells were seeded randomly in the each well of different well-plates for compound treatments.
Blinding	Blinding was not relevant to this study. Data were derived from instrument-based measurement and software-based analysis.

Reporting for specific materials, systems and methods

We require information from authors about some types of materials, experimental systems and methods used in many studies. Here, indicate whether each material, system or method listed is relevant to your study. If you are not sure if a list item applies to your research, read the appropriate section before selecting a response.

Materials & experimental systems

n/a	Involved in the study	
☒	☐	Antibodies
☐	☒	Eukaryotic cell lines
☒	☐	Palaeontology and archaeology
☒	☐	Animals and other organisms
☒	☐	Clinical data
☒	☐	Dual use research of concern

Methods

n/a	Involved in the study	
☒	☐	ChIP-seq
☒	☐	Flow cytometry
☒	☐	MRI-based neuroimaging

Eukaryotic cell lines

Policy information about cell lines and Sex and Gender in Research

Cell line source(s)	Fibroblasts derived from Gaucher patients (GM00372 and GM00877) were purchased from Coriell Institute (Camden, NJ).
Authentication	No authentication was performed.
Mycoplasma contamination	Cells were confirmed to be free of mycoplasma by using Mycoplasma Detection Kit (InvivoGen).

March 2021

Commonly misidentified lines<br>(See ICLAC register)	No commonly misidentified cell lines were used in this study.
